# Widespread winners and narrow-ranged losers: Land use homogenizes biodiversity in local assemblages worldwide

**DOI:** 10.1371/journal.pbio.2006841

**Published:** 2018-12-04

**Authors:** Tim Newbold, Lawrence N. Hudson, Sara Contu, Samantha L. L. Hill, Jan Beck, Yunhui Liu, Carsten Meyer, Helen R. P. Phillips, Jörn P. W. Scharlemann, Andy Purvis

**Affiliations:** 1 Centre for Biodiversity and Environment Research, Department of Genetics, Evolution and Environment, University College London, London, United Kingdom; 2 Department of Life Sciences, Natural History Museum, London, United Kingdom; 3 UN Environment World Conservation Monitoring Centre, Cambridge, United Kingdom; 4 University of Colorado, Museum of Natural History, Boulder, Colorado, United States of America; 5 College of Agricultural Resources and Environmental Sciences, China Agricultural University, Beijing, China; 6 German Centre for Integrative Biodiversity Research (iDiv), Leipzig, Germany; 7 Faculty of Biosciences, Pharmacy and Psychology, University of Leipzig, Leipzig, Germany; 8 Department of Life Sciences, Imperial College London, London, United Kingdom; 9 School of Life Sciences, University of Sussex, Brighton, United Kingdom; Ecole Normale Superieure, France

## Abstract

Human use of the land (for agriculture and settlements) has a substantial negative effect on biodiversity globally. However, not all species are adversely affected by land use, and indeed, some benefit from the creation of novel habitat. Geographically rare species may be more negatively affected by land use than widespread species, but data limitations have so far prevented global multi-clade assessments of land-use effects on narrow-ranged and widespread species. We analyse a large, global database to show consistent differences in assemblage composition. Compared with natural habitat, assemblages in disturbed habitats have more widespread species on average, especially in urban areas and the tropics. All else being equal, this result means that human land use is homogenizing assemblage composition across space. Disturbed habitats show both reduced abundances of narrow-ranged species and increased abundances of widespread species. Our results are very important for biodiversity conservation because narrow-ranged species are typically at higher risk of extinction than widespread species. Furthermore, the shift to more widespread species may also affect ecosystem functioning by reducing both the contribution of rare species and the diversity of species’ responses to environmental changes among local assemblages.

## Author summary

Previous studies have shown that human use of the land, mainly for agriculture and settlements, causes a detectable but relatively small net loss of biodiversity. However, not all species are affected equally, and some species even benefit from the new habitats we create. One group of species of particular concern for biodiversity conservation are those that inhabit only a small area. These narrow-ranged species are at higher risk of extinction because it is more likely that any threats to the species (including human land use) will affect their entire range. Such species can also play a unique role in the healthy functioning of ecosystems. Here, we show that the observed small declines in biodiversity in human-disturbed land can be broken down into large declines in narrow-ranged species, offset by increases in wide-ranged species. All else being equal, this finding means that ecological communities are losing their distinctive, narrow-ranged species and are becoming dominated by the same species everywhere, leading to a reduction in global biodiversity. The divergent effects of human land use on narrow-ranged and widespread species are important for the conservation of already threatened, narrow-ranged species and may lead to a negative effect on the functioning of ecosystems.

## Introduction

Human impacts on the biosphere are substantially reducing the global number of species [1,2], and many scientists argue that we have entered a new, human-dominated geological era, the ‘Anthropocene’ [3]. Land use is currently among the predominant pressures on biodiversity globally [4], with substantial net losses of species in land-use types dominated by human activities [5]. However, not all species are equally impacted, and many species benefit from human creation of novel habitats [6]. This results in strong turnover in community composition between land-use types [5,7]. Understanding the characteristics of species that are most impacted by human land use is essential for guiding conservation actions and also for assessing the consequences of land-use change and biodiversity loss for the functioning of ecosystems.

Geographic range size is a fundamental property of species. Crucially, small range size is a key determinant of species’ extinction risk [8–10], and therefore a disproportionate impact of human land use on small-ranged species would have important consequences for biodiversity conservation. Furthermore, rare species (including geographically restricted species) tend to have unique combinations of functional traits and are therefore likely to make an important contribution to ecosystem functioning [11], meaning that a disproportionate loss of small-ranged species would have implications beyond their immediate conservation interest. A few geographically and taxonomically restricted studies have shown that narrow-ranged species are more likely than wide-ranged species to be absent in human land uses [12–15], leading—all else being equal—to a homogenization of assemblage composition across space [16]. A global, multi-clade analysis for terrestrial ecosystems has so far been prevented by a lack of suitable global datasets (but see [17] for a global freshwater analysis), and thus the generality of this pattern remains unclear.

We present the first global synthesis of the effect of land use on an abundance-weighted measure of the average range size of species in ecological assemblages of plants, invertebrates, and vertebrates. We refer to this measure as Relative Community Average Range Size (RCAR). Since samples of abundance are uncertain, and any patterns in measures of average range size that are weighted by species’ abundance could be driven by the few most abundant species, we also present results for a range-size measure unweighted by abundance, which we refer to as Relative Average Range Size (RAR). Range sizes were log_10_-transformed prior to averaging to reduce the influence on the measures of the few very widespread species. Increases in RCAR or RAR, which reflect an increased dominance of more widespread species within communities, would indicate that community composition is tending to become homogenized globally [16]. Our broad-scale synthesis enables us to assess geographic and taxonomic variation, as well as quantify overall patterns.

Homogenization of ecological assemblages has traditionally been assessed as a reduction in spatial turnover (beta diversity) in assemblage composition [18,19]. However, this approach has limitations because the effects of land conversion on beta diversity are scale dependent [20]. For example, partial conversion of a natural landscape can increase beta diversity at the landscape scale while decreasing it globally. In contrast, measures based on the average range size of species within a community, such as RCAR and RAR used here, are always expected to increase whenever homogenization occurs.

**Fig 1:**
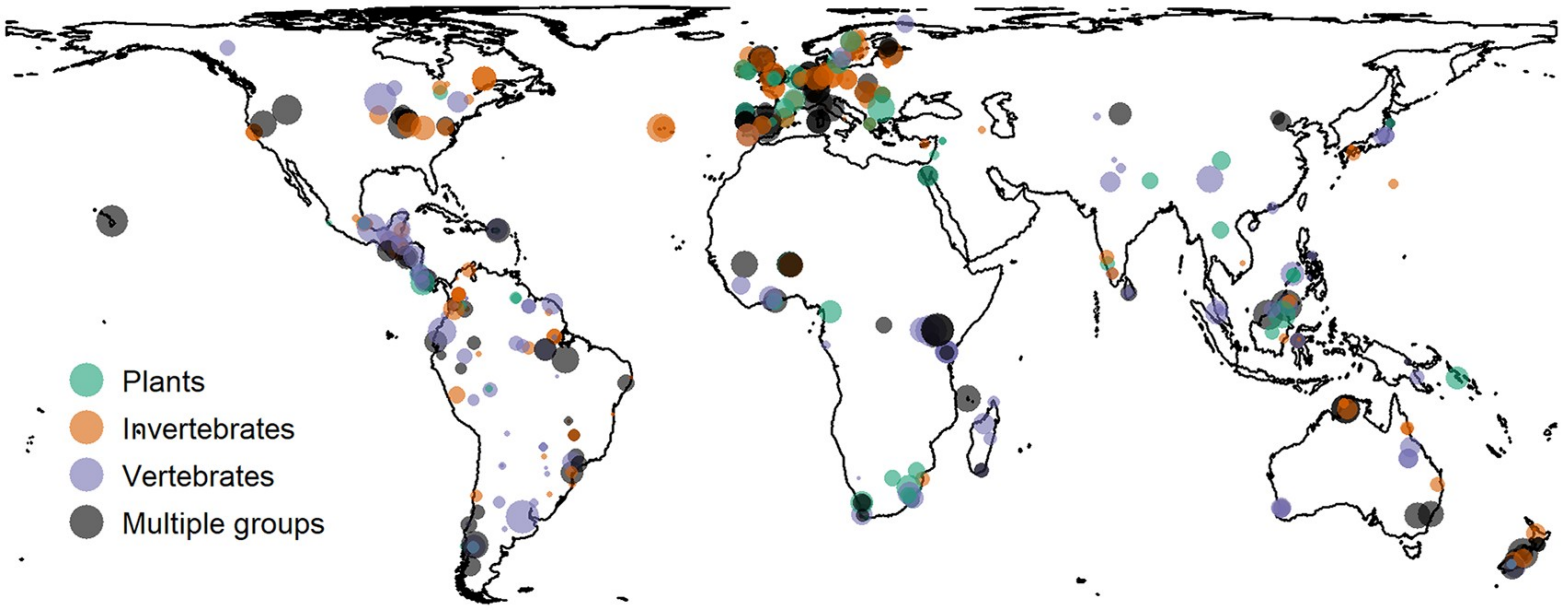
Locations of surveys whose data were used in the analysis, by major taxonomic group of study. Shown in the Lambert cylindrical equal area projection. Point diameters are proportional to the (log_e_) number of sites sampled by each survey, and are translucent so areas of opaque colour indicate overlapping points. Studies are coloured according to the taxa sampled within the study. The outline map is based on the World Bank map of river basins (https://bit.ly/2J86Kbq), which is published under a CC-BY 4.0 license. The site-level data underlying this figure are freely available (DOI: 10.6084/m9.figshare.7262732).

We combined a dataset of 1.1 million records from 445 surveys (Fig 1 and S1 Fig) that compared the abundance of 19,334 species of terrestrial plants (7,111 species), invertebrates (7,048 species), and vertebrates (5,175 species) among different land uses [21], with occupancy- or extent-based estimates of all species’ range sizes using records from the Global Biodiversity Information Facility (GBIF; https://www.gbif.org) or from expert-drawn extent-of-occurrence maps for vertebrates only, to calculate RCAR and RAR at 13,292 sites in different land uses (see Materials and methods for full details).

Estimates of global range size are uncertain for most terrestrial species [22,23], with both taxonomic and geographic patterns of underrecording hampering broad comparisons of absolute range-size estimates [22,24]. These problems are mitigated to some extent in our analyses because each of the individual biodiversity surveys—from which the abundance data were taken—sampled within relatively restricted taxonomic groups and geographic regions, and our hierarchically structured models account for differences in average range sizes between surveys. Therefore, our models only require good estimates of relative range size within the taxonomic groups and geographic regions of the individual studies. Nevertheless, to test the robustness of our results, we repeat all of our analyses for 3 different metrics of range size based on 2 fundamentally different underlying datasets: the occupancy of grid cells based on records in the GBIF database, the extent of land area encompassed by records in the GBIF database (excluding outlying records), and the total area within expert-drawn maps of species ranges for amphibians, mammals, and birds (see Materials and methods for full details). We also test the sensitivity of our results to variation in the resolution of the grid used to estimate range occupancy and extent, and to variation in the completeness of the underlying GBIF records.

RCAR and RAR were modelled as a function of land-use type and land-use intensity, human population density, distance to the nearest road, and coarse landscape-scale land-use history. Land-use type and land-use intensity were classified based on the description of the habitat where assemblages were sampled, as given in the underlying papers (see S1 Text). Responses may also be shaped by current land use in the surrounding landscape and by the detailed local history of land use, but reliable estimates of these factors were not available for the vast majority of sites included in our analyses. We used mixed-effects models to control for differences in sampling methods, sampling effort, average estimated range size, and climate among surveys, and for spatial patterns in the sampled sites within each survey. Average range sizes and the prevalence of different land uses are both likely to vary with elevation, so we also fitted elevation in the models as a fixed effect. In separate models, we tested whether responses differed geographically—as a function of the division between the tropical and temperate realms or as a function of climatic and topographic differences—and taxonomically, by comparing plants, invertebrates, amphibians, reptiles, mammals and birds.

Homogenization is one explanation that can reconcile large rates of global extinction of species with relatively small net changes in local species richness [25]. In this case, we would expect responses of species richness and of RCAR and RAR to be more or less decoupled from one another. To test this, we assessed whether local (per-survey) responses of RCAR and RAR to land-use type were correlated with responses of species richness to land use in the same locations (i.e., the same surveys; see Materials and methods for details).

An increase in RCAR or RAR (and thus homogenization) could be caused by the loss of species with narrow geographic ranges [26,27], which tend also to be ecologically specialized [28], and/or by the gain of widespread species [26,27], which are often either species introduced from elsewhere [29] or ruderal, pioneer, or generalist native species able to exploit anthropogenic habitats [30]. To test whether either or both of these processes explain observed land-use effects on RCAR and RAR, we split the species recorded within each taxonomic Class and within each survey into 3 equal groups based on their range sizes. We then modelled both the total abundance and species richness of each of these groups as a function of land-use type and land-use intensity.

## Results

### Overall effects of land use and related pressures

**Fig 2:**
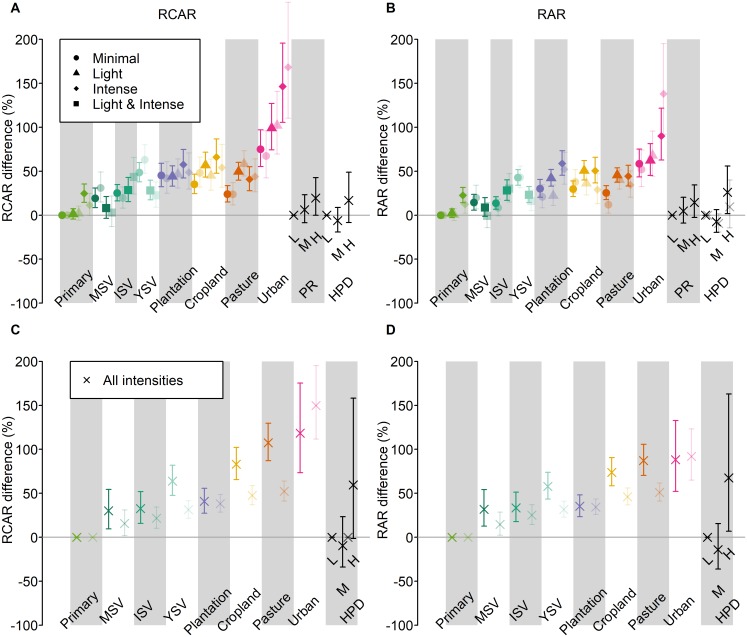
Modelled effects of human pressures on RCAR. Effects are shown as a percentage difference from the value in minimally used primary vegetation, and were taken from the best-fitting models among those having no interactions between explanatory variables (results including interactions are shown in S3 Fig). Human pressures considered were as follows: land-use type and intensity, proximity to roads, and human population density. Error bars show 95% confidence intervals. Land-use type was classified as: primary vegetation, mature secondary vegetation, intermediate secondary vegetation, young secondary vegetation, plantation forest, cropland, pasture, and urban. We considered 3 alternative approaches for estimating species’ range size: range occupancy based on GBIF records (opaque points in A and B), range extent based on GBIF records with outliers removed (translucent points in A and B), and, for vertebrates only, range extent based on expert-drawn range maps (opaque points in C and D). For comparison, we also show estimates based on range occupancy from GBIF records for vertebrates (translucent points in C and D). We considered community-average range sizes both weighted (RCAR; A and C) and unweighted (RAR; B and D) by species’ abundance. For the models of all taxonomic groups (A and B), each land-use class was subdivided into 3 levels of human intensity of use—minimal, light, and intense (see S4 Table)—as indicated by different plotting symbols (light and intense intensity levels were combined for the secondary-vegetation classes). Effects of proximity to roads, vegetation removal, and human population density are shown here at the lowest, median, and highest values in the modelled dataset. Proximity to roads as shown here is the reverse of the distance to nearest road measure that was fitted in the models. Minimally used primary vegetation furthest from a road, and at the lowest level of vegetation removal and human population density, was used as the baseline. The site-level data underlying the models shown here are freely available (DOI: 10.6084/m9.figshare.7262732). GBIF, Global Biodiversity Information Facility; H, highest values in the dataset for each continuous effect; HPD, human population density; ISV, intermediate secondary vegetation; L, lowest values in the dataset for each continuous effect; M, median values in the dataset for each continuous effect; MSV, mature secondary vegetation; Plantation, plantation forest; PR, proximity to roads; Primary, primary vegetation; RCAR, Relative Community Average Range Size; YSV, young secondary vegetation.

Overall, land use had a strong effect on both RCAR and RAR. Assemblages in all human-dominated land uses (plantation forests, croplands, pasture, and urban environments) have higher average RCAR than those in natural vegetation types (Fig 2) Even within natural vegetation, RCAR was higher in secondary vegetation (especially secondary vegetation in an early stage of recovery) than in primary vegetation (Fig 2) The effects of land use on RCAR were strong, significant, and very similar—qualitatively and quantitatively—regardless of which measure of range size was used. The results were also very similar whether average range size was weighted by abundance (RCAR) or not (RAR) (all likelihood ratio tests: χ^2^ > 153, *P* < 0.001; Fig 2), showing that the patterns were not driven by responses of a few very abundant species. The consistency in the modelled effects of land use across range-size metrics occurred despite the underlying site-level RCAR values not always correlating strongly (R^2^ values between 0.26 and 0.66; S2 Fig). The low correlations are unsurprising given that the metrics were chosen to reflect very different properties of species’ ranges and are based on completely different underlying datasets. Within land-use categories, RCAR increased significantly with increasing human land-use intensity (χ^2^ > 7.0; *P* < 0.03), to an extent that differed among land-use types (interaction between land-use type and intensity: χ^2^ > 249, *P* < 0.001; Fig 2). Additionally, RCAR also independently increased significantly with human population density in 7 of the 8 models (for which χ^2^ > 6.2; *P* < 0.012), although the relationships were much weaker (Fig 2) and differed among land-use types with strong increases only in cropland and pasture (interactions with land-use type: for all models, χ^2^ > 68; *P* < 0.001; S3 Fig). The effects of proximity to roads (in all models, χ^2^ < 1.4, *P* > 0.23) and in most cases duration of human use of a landscape (in 6 out of 8 models, χ^2^ < 3.1, *P* > 0.08) did not affect RCAR on their own. However, both effects often interacted significantly with land-use type (interaction between land-use type and proximity to roads: in 6 out of 8 models, χ^2^ > 26.6, *P* < 0.022; interaction between land-use type and duration of landscape use: for all models, χ^2^ > 15.3, *P* < 0.033; S3 Fig). Specifically, a longer history of human domination of landscapes was associated with a strong increase in RCAR in urban environments but with decreased RCAR in cropland, plantation forest, pasture, and mature secondary vegetation (S3 Fig); RCAR increased with increasing proximity to roads in most land-use types, except young and mature secondary vegetation (S3 Fig). The per-survey strength of response of species richness to land-use type was weakly negatively correlated with the response of RCAR to land-use type (F_1,426_ = 7.09, *P* = 0.008; R^2^ = 0.016; S4 Fig).

### Geographic and taxonomic variation in responses

**Fig 3:**
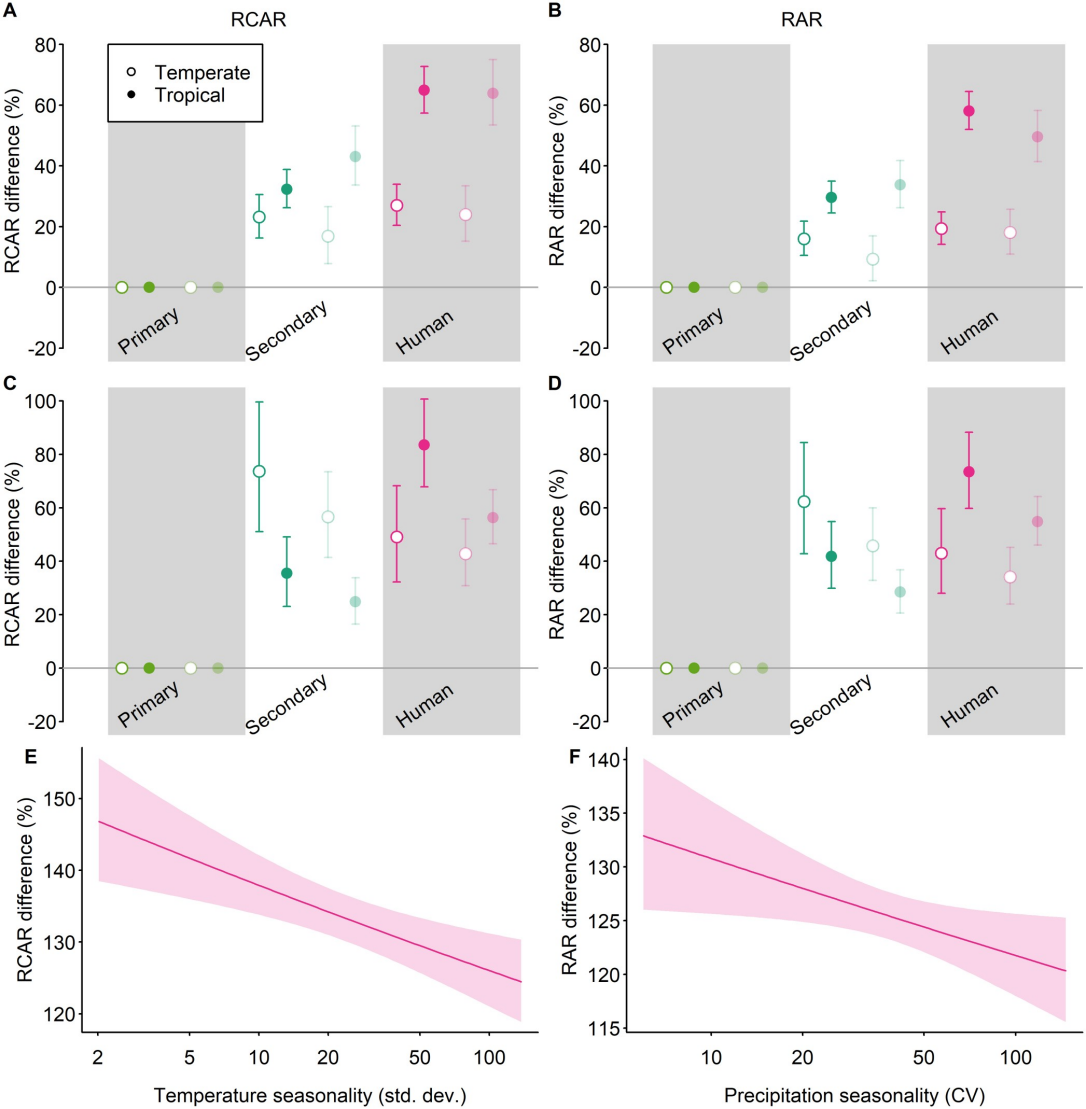
Geographical variation in the response of RCAR to land use. We first tested for tropical-temperate differences in observed responses to disturbed land uses (A–D), and then whether responses to human land use vary with climatic seasonality or topographic heterogeneity (significant results are shown in E–F). All effects are shown as a percentage difference from values in primary vegetation. Error bars (A–D) and shading around lines (E–F) show 95% confidence intervals. For the analyses shown here, the land-use classification was coarsened to 3 classes: primary vegetation, secondary vegetation (all stages of recovery), and human-dominated (plantation forest, cropland, pasture, and urban). Land-use intensity was not considered. For the tropical-temperate models (A–D), as in the main models, we considered 3 measures of range size, with community-average range size both weighted (RCAR; A, C, and E) and unweighted (RAR; B, D, and F) by species’ abundance; these different combinations are plotted as in Fig 2. The site-level data underlying the models shown here are freely available (DOI: 10.6084/m9.figshare.7262732). CV, Coefficient of variation; RAR, Relative Average Range Size; RCAR, Relative Community Average Range Size.

There was a significant difference in the responses of RCAR and RAR to land use between the tropical and temperate realms (χ^2^ > 196, *P* < 0.001). RCAR increased much more strongly in human-dominated land uses in the tropics than in the temperate realm (Fig 3). This result was qualitatively and quantitatively very similar across all range-size measures and for both RCAR and RAR. The tropical-temperate difference could be explained by the fact that increases in RCAR and RAR in human land uses were stronger in locations with lower climate seasonality: temperature seasonality in the case of RCAR (Fig 3; full statistical results in S1 Table) and precipitation seasonality in the case of RAR (Fig 3; full statistical results in S2 Table). Climate seasonality has previously been shown to be important in determining species’ range sizes [31] and responses to land-use change [7]. RCAR and RAR also increased more strongly in secondary vegetation in the tropics across all species in the dataset (Fig 3). However, for vertebrates, responses in secondary vegetation were more strongly positive in the temperate realm (Fig 3).

**Fig 4:**
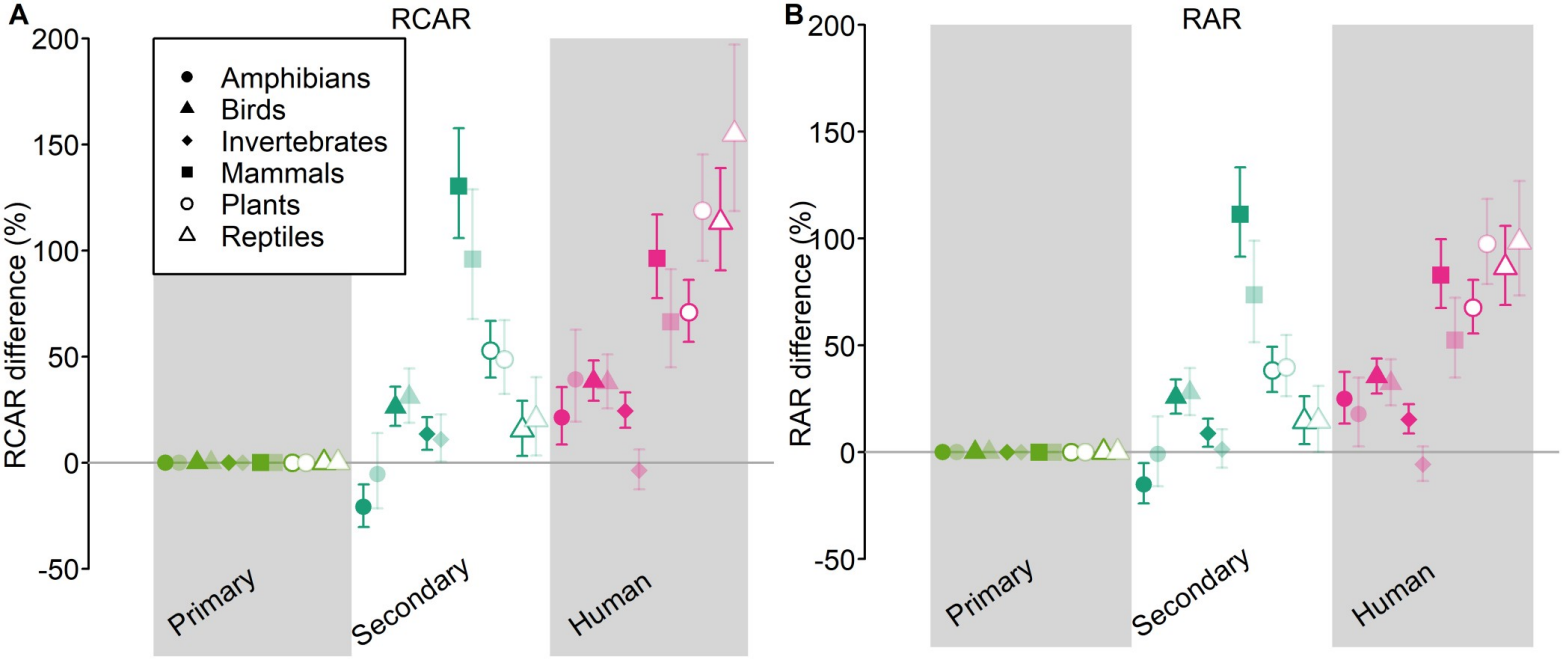
Differences among taxonomic groups in the response of RCAR to land use. Effects are shown as a percentage difference relative to primary vegetation. Error bars show 95% confidence intervals. For these analyses, the land-use classification was coarsened to 3 classes: primary vegetation, secondary vegetation (all stages of recovery), and human-dominated (plantation forest, cropland, pasture, and urban). Land-use intensity was not considered. Primary vegetation was used as the baseline. As in the main models, we considered community-average range size both weighted (RCAR; A and C) and unweighted (RAR; B and D) by species’ abundance; these different combinations are plotted as in Fig 2. The site-level data underlying the models shown here are freely available (DOI: 10.6084/m9.figshare.7262732). RAR, Relative Average Range Size; RCAR, Relative Community Average Range Size.

There were also significant differences in the responses of RCAR and RAR to land use among different taxonomic groups (χ^2^ > 219; *P* < 0.001). RCAR and RAR increased in human-dominated land uses most strongly for reptiles, plants, and mammals, and least for invertebrates (Fig 4). We caution that differences among taxonomic groups may partly reflect differences in the quality of range-size estimates (see Discussion).

### Responses of species with different relative range sizes

**Fig 5:**
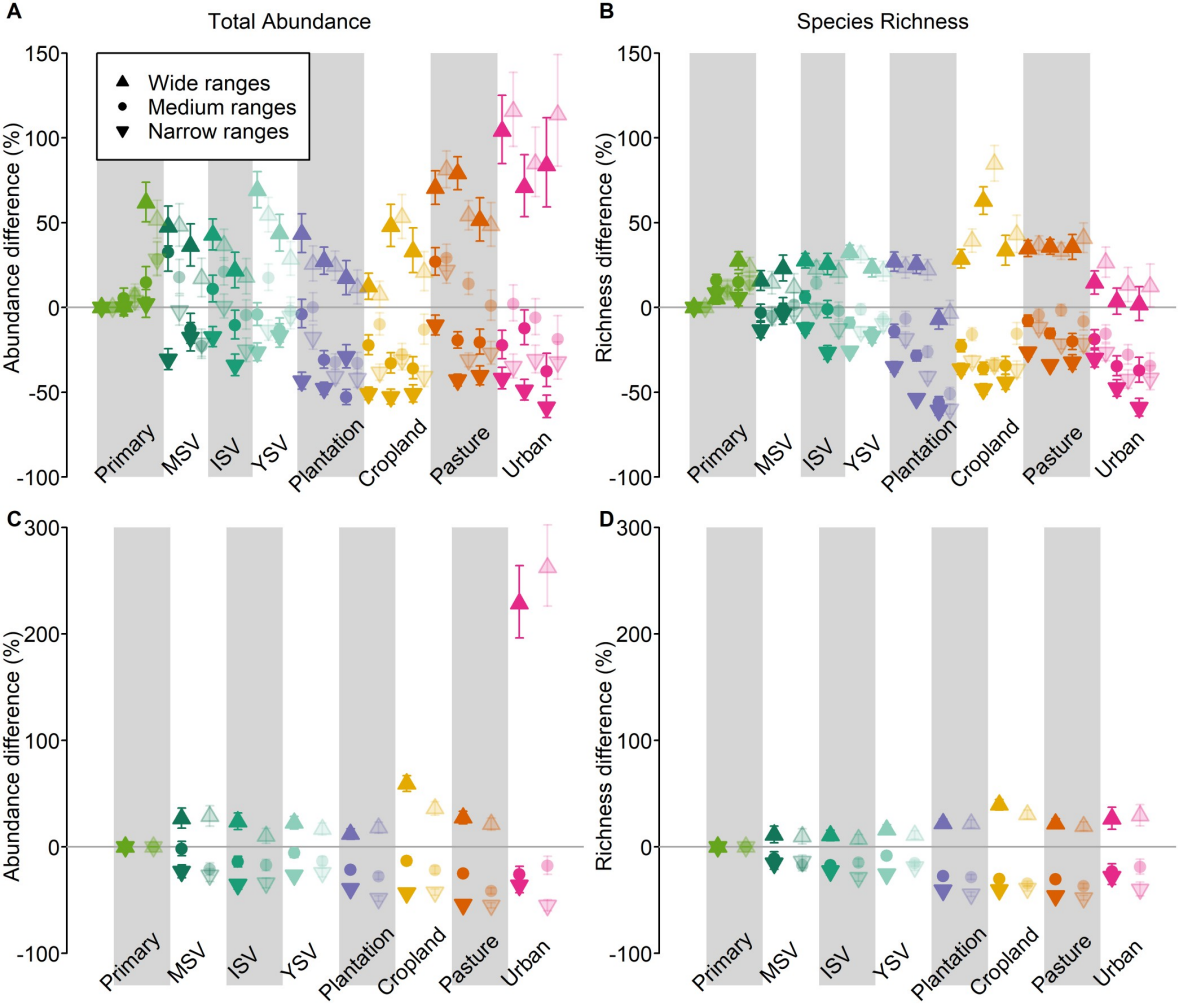
Effects of land-use type and land-use intensity on the total abundance or species richness of species with different range sizes. Effects are shown as a percentage difference relative to minimally used primary vegetation. Total abundance (A, C) and richness (B, D) are back-transformed to their original scales. For clarity, error bars here show ±1 standard error. The species sampled within each taxonomic Class within each survey were separated into 3 equal groups by range size into those with the widest ranges (upward-facing triangles), medium-sized ranges (circles), and narrowest ranges (downward-facing triangles). As in the main models, we either considered species’ abundance (i.e., modelling total abundance: A and C) or not (i.e., modelling species richness: B and D); we also considered 3 different metrics of range size for all species or for vertebrates only, with these combinations plotted as in Fig 2. The site-level data underlying the models shown here are freely available (DOI: 10.6084/m9.figshare.7262738). ISV, intermediate secondary vegetation; MSV, mature secondary vegetation; Plantation, plantation forest; Primary, primary vegetation; YSV, young secondary vegetation.

Homogenization implies that narrow-ranged species tend to decline in occurrence or abundance, that widespread species tend to increase, or both. Splitting each survey’s species into 3 equal groups based on their range sizes revealed that land use and related pressures cause both of these changes (Fig 5). Narrow-ranged species have much lower local abundances on average (often by 30%–50%) under nearly all land-use classes compared with primary vegetation, while already widespread species increased in abundance by a similar amount. Species with mid-sized ranges also tended to have lower abundances in nonprimary land-use classes, although not by as much as narrow-ranged species (Fig 5). As with the previous results, modelled patterns were qualitatively and quantitatively similar across all range-size metrics, and for both total abundance and species richness differences (Fig 5).

### Sensitivity analyses

In addition to being consistent across the different methods of calculating both RCAR and RAR, our results were also generally very robust in sensitivity tests, in which we used subsets of the vertebrate and plant data with range-size estimates restricted to taxa and regions with successively more complete GBIF data ([22]; see S5 and S6 Figs). The magnitude of land-use effects on RCAR was reduced—but still significant—in the most stringent subsets of the data (S5 Fig). However, the step at which this change occurred was associated with the loss of almost all tropical sites (S7 Fig), which we show above to be the most strongly affected by human land use. Nevertheless, if the large effects seen in the tropics were caused by poor estimates of range size, they entailed uncertainty only in the degree to which widespread species had higher abundance in disturbed habitats (S6 Fig); the reduced abundance of narrow-ranged species was consistent across all data subsets (S6 Fig).

The results were also very similar when we used different spatial resolutions to grid the GBIF data in order to estimate range sizes (S8 Fig). If anything, the effects of land use were stronger when we used a finer-resolution grid than that used for the results presented in the main text (S8 Fig). We tested for spatial autocorrelation in the residuals associated with each of the underlying surveys (as in [5]), finding significant spatial autocorrelation no more often than expected by chance (S9 Fig; one-sided equality of proportions test: χ^2^ < 1.52, *P* > 0.10). Furthermore, the residuals of the model showed no pattern with respect to latitude (S10 Fig), despite geographic gradients in average range size [32].

## Discussion

In most parts of the world, human populations are growing [33], human-dominated land uses and secondary vegetation are increasing at the expense of primary vegetation [34], and the road network is being expanded [35]. Our models show that each of these pressures leads to an increase in the average range size of species within ecological assemblages, confirming the global—and in most cases taxonomic—generality of a pattern previously demonstrated by smaller-scale, single-clade studies [12–15], consistent with a similar global synthesis for freshwater fish assemblages [17]. The results have important implications for the conservation of species, sustainable land-use practices, and likely also for ecosystem functioning.

The increases in RCAR and RAR in disturbed habitats also suggest a homogenization of local ecological assemblages worldwide. Previous detailed studies across well-studied regions have shown that human land use homogenizes biodiversity, increasing the similarity between neighbouring communities [19]. Our results imply that human land use is causing a similar homogenization of community composition for many different taxonomic groups globally. Although the surveys with the most negative responses of local species richness to human land use tended also to show the strongest increases of RCAR in human land uses (S4 Fig), the weakness of this relationship (R^2^ = 0.016) suggests that changes in richness and RCAR are largely decoupled. This combination of results is consistent with homogenization as a mechanism that reconciles the observed large global losses of biodiversity—as well as the regional and national declines in many species—with the relatively small average net change in local diversity [5,25]. Whereas the balance between species loss and species gain may depend on context, homogenization is occurring in all disturbed habitats.

Our results have important implications for species conservation. In general, species with narrow ranges are the most likely to be threatened with extinction because the drivers of threat are more likely to affect the entire range of these species [8]. More specifically, narrow ranges will tend to reflect more specific climatic requirements and thus greater sensitivity to climate change. Given this, our results suggest that the same species are likely to be sensitive to both land use and climate change. The contrasting effects of land use on the abundance of narrow- and wide-ranged species highlights the importance of primary vegetation for species with small ranges [36]. Even mature secondary vegetation—whose assemblages may approach those in primary vegetation in terms of species richness [5,37], total abundance [5], and biomass [37]—has a substantially reduced abundance of narrow-ranged species compared with primary vegetation (Fig 5). Our study adds new weight to calls for larger investments in the study and conservation of narrow-ranged species. It also highlights the additional insight available from using biodiversity metrics that reflect assemblage composition as well as numbers of taxa or individuals [38,39].

The changes in community-average range size also have implications at the ecosystem level. Because widespread species tend to be ecological generalists [28], the increases in RCAR and RAR that we show probably indicate functional homogenization at a global scale. Previous studies have suggested that narrow-ranged species may be functionally distinct, potentially making an important contribution to resilient ecosystem function [11]. The increased abundance of widespread species may also drive spatial synchrony among assemblages and populations within landscapes, increasing the risk of extreme dynamics [40].

Increases in RCAR and RAR were strongest in the tropical realm. There are three possible—likely interconnected—reasons for this difference. First, native tropical species tend to have smaller average ranges than temperate species [32], increasing the contrast with wide-ranging newcomers. Second, tropical species—especially narrow-ranged species—may be more specialized, on average, than temperate species [41]. Third, temperate regions have experienced a longer history of large-scale human disturbance than most tropical regions, which has likely already filtered out the most sensitive species from assemblages and expanded the range of disturbance-tolerant species [42]. The first two explanations may be associated with disproportionately strong responses in environments with low climatic seasonality or topographic heterogeneity because climatically stable environments are characterized by species with smaller average range sizes [31] and more specialized species [43]. Indeed, we showed that the stronger responses of average community range sizes in tropical areas could be better explained by the lower climatic seasonality of these environments than by the tropical-temperate division alone (Fig 3). The disproportionate increases in RCAR and RAR in the tropics have important implications for the future conservation of biodiversity, given that most species—and especially narrow-ranged species—are found in the tropics and that most future land conversion is predicted to occur here [44].

Responses of community-average range sizes varied strongly among taxonomic groups, with the largest increases in RCAR and RAR in human-modified land uses for reptiles, plants, and mammals, and the weakest for invertebrates. The reasons for these taxonomic differences are not clear, and we caution that they may to some extent reflect variation in the quality of range-size estimates. Varying quality of range-size estimates is especially likely to explain the weak responses for invertebrate species, given that invertebrate ranges are likely to be the most incompletely defined, and narrow-ranged invertebrates have been shown to be less likely to be captured in incomplete surveys [45]. However, the particularly strong responses of reptiles highlight the conservation concern of a group that—among vertebrates—has already been shown to respond most strongly to human land use and whose species are likely to have their ranges most reduced by future climate change [46]. The broadly consistent responses across most taxonomic groups, especially for those groups that likely have better-characterized ranges, provide further evidence that the overall results are not likely to be an artefact of poor range-size estimates.

Such a broad-scale analysis necessarily entails many uncertainties. The greatest challenge here was in the estimation of range sizes. For most species, highly accurate range-size estimates will not be available for the foreseeable future. Therefore, we selected estimates of range size that were as different as possible in nature (e.g., occupancy versus extent) and in the underlying data and methodology, which resulted—by design—in site-level RCAR and RAR measures that were not always very strongly correlated (S2 Fig). Nevertheless, the modelled effects of land use were generally very robust to (a) using different range-size metrics (including expert-drawn range maps, which suffer less from the biases that characterize GBIF data) (Figs 2–5), (b) the choice of whether or not to weight by recorded species abundances (Figs 2–5), (c) the removal of sites where the underlying range-size estimates may be less reliable (S5 and S6 Figs), and (d) the use of different grid resolutions to estimate range sizes (S8 Fig). Improved knowledge of species’ ranges will in time refine estimates of RCAR and RAR.

As with any correlative analysis, it is impossible to determine the mechanisms behind observed patterns with confidence. For example, the disproportionate sensitivity of narrow-ranged species to land use may reflect the fact that these species tend also to be rare in other ways, such as having low abundance or being habitat specialists [47]. How different forms of rarity interact to determine sensitivity to land use is an important question for future studies. Our analyses also ignore how responses to land-use change unfold over time, though these dynamics will often be important [48]. Spatial comparisons of RCAR and RAR among land uses do not provide a direct measure of the temporal homogenization of biodiversity.

All studies based on samples of biodiversity in different land uses are potentially biased by differences in detection probability among species and among locations. We expect numerically rare species to be undersampled relative to abundant species, especially in the more closed habitats typical of natural vegetation. If anything, numerically rare species are likely also to have narrow ranges ([49], but see e.g. [50]), in which case our modelled effects of land use on RCAR will be conservative. We have expressed changes in RCAR and abundance relative to values from assemblages in minimally used primary vegetation, but even such ‘pristine’ assemblages will have been influenced by humans in many cases [51,52]; any bias caused by unrecognised past human influence will—by introducing widespread species into sites treated as pristine—have diluted rather than strengthened the effects we see. Another potential issue is that range sizes show biogeographical patterns that are unrelated to land-use differences, in particular tending to increase with latitude [31]. However, sites within most of the surveys in our dataset span a very small latitudinal range (the sites sampled in more than 92% of surveys ranged over less than 1° of latitude), and the residuals from our main model showed no latitudinal (or longitudinal) pattern (S10 Fig).

Overall, our results provide strong and consistent evidence that land use and related pressures reduce the abundance of narrow-ranged species while increasing the abundance of widespread species. This will, on average, lead to a homogenization of terrestrial ecological assemblages worldwide. The disproportionate loss of narrow-ranged species in human land uses is of concern for biodiversity conservation and ecosystem functioning, given that these species tend to be at the greatest risk of extinction and may play unique functional roles.

## Materials and methods

### Assemblage composition data

We extracted data from the Projecting Responses of Ecological Diversity In Changing Terrestrial Systems (PREDICTS) database [21,53] on 28 April 2015. This database is a collation of individual studies that compared species presence/absence or abundance among different types of land use. The data used in this study consisted of a total of 1,127,401 records of abundance (presence/absence records were excluded) that could be matched to a corresponding estimate of range size (see below). These records were taken from 368 sources—published or in-press papers, or unpublished datasets with published methods (a full reference list is given in S1 Text)—that contained data from 445 surveys, collectively sampling 13,292 sites (S1 Fig), spread across all but one of the world’s terrestrial biomes (flooded grasslands/savannas were not sampled), approximately in proportion to total productivity (S1 Fig). The dataset contained records for 19,334 species in total, with no strong taxonomic bias, and records for over 1% of described species in many major taxonomic groups (S1 Fig). Abundance records were corrected for any variation in sampling effort within studies (which affected 16% of studies) by assuming that recorded abundance increases linearly with effort [54]. More sophisticated methods for correcting the abundance estimates could not be used because most of the underlying studies did not make repeat visits to each site. However, for uncertainty in the abundance estimates to bias our results would require that recorded abundances were biased with respect to both range size and land use. This type of bias seems unlikely, and so we expect any uncertainty in the recorded abundances simply to add noise to the modelled relationships. We obtained geographic coordinates for all sites from the source publications or their authors.

### Estimating range size

Estimates of the global range size of species always contain considerable uncertainty. In this study, we estimated range size using 3 different metrics and repeated the analyses with each metric. The measures used were as follows: (1) the area occupied by species based on records in the GBIF database—‘GBIF occupancy’ (the measure focused on in the main text); (2) an estimate of the extent of species’ ranges based on GBIF records—‘GBIF extent’; and (3) an estimate of range extent based on expert-drawn extent-of-occurrence maps (available only for vertebrate species)—‘Expert extent’. Metric 1 captures a different aspect of range size (occupancy) than that captured by metrics 2 and 3 (extent). All GBIF records for plants, arthropods, vertebrates, molluscs, and annelids were downloaded (http://www.gbif.org) on 25 June 2015. These raw extracts were then filtered to contain only species also found in the PREDICTS database.

GBIF occupancy was estimated by summing the total area of the grid cells containing a record in the GBIF database. This approach will underestimate ranges when recording is incomplete but avoids overestimating ranges of species with poor range filling and is much less affected by incorrectly located records than extent-based measures. Estimates obtained in this way will vary depending upon the spatial resolution of the grid used. Using a finer resolution should better represent the range sizes of narrow-ranged species but also increases the influence of biases and incompleteness in GBIF records. To ensure the robustness of our results to the choice of grid, we generated estimates using equal-area grids with 3 different grid sizes: 110 km × 110 km, 55 km × 55 km, and 11 km × 11 km. We focus in the main text on the results using the 110-km grid because, at finer resolution, the patchiness of the GBIF data is likely to create greater discrepancies across species.

To estimate GBIF extent, we first defined a rectangle bounded by the pair of longitudes that encompassed the central 95% of GBIF records in a longitudinal direction as well as the pair of latitudes that encompassed the central 95% of GBIF records in a latitudinal direction. This exclusion of outlying records mitigates to some extent the effect of the incorrectly located records present within GBIF. The rectangle was defined as a grid of cells of flexible resolution. We clipped the gridded rectangle using a gridded land-sea mask to represent only terrestrial areas. The land-sea mask grid was derived from the World Borders dataset (www.thematicmapping.org) using the rasterize function in the raster package Version 2.3–12 [55] in R Version 3.1.2 [56]. The total range extent was then calculated as the sum of the area of all of the land cells within the rectangle. Grid cell area was calculated as above. To test the robustness of the range-extent estimates to the use of different grids, we used 3 spatial resolutions (as above): 110 km, 55 km, and 11 km. We used this simple measure of range extent rather than more sophisticated extent measures based on, for example, minimum convex polygons because the latter measures would be sensitive to gaps and biases in the GBIF records to a much higher degree than both simple latitudinal and longitudinal extent and the main occupancy-based measure based on GBIF data. In a well-recorded arthropod group (the sphingid moths), range estimates based on simple latitudinal and longitudinal extent have been shown to correlate strongly (R^2^ = 0.94) with those based on minimum convex polygons [57].

Records in the GBIF database are biased geographically [22], biasing relative estimates of range size, and possibly also comparisons of average assemblage range sizes among land uses. For comparison with our GBIF occupancy and extent estimates, we also used expert-derived estimates of extent, based on expert-drawn extent-of-occurrence maps for amphibians [58], mammals [58], and birds [59]. Three steps were used to process the extent-of-occurrence maps, using the arcpy Python module associated with ArcMap Version 10.3 [60] (steps 1 and 2) and using R Version 3.1.2 [56] (step 3). First, the polygon maps were projected onto a Behrmann equal-area projection using the ‘Project_management’ function. Second, the area of each polygon within a species’ range map was calculated using the ‘CalculateAreas_stats’ function. Finally, for each species, the areas for every polygon were summed.

The average range size of species within assemblages was measured in two ways: first, as the community-weighted mean (log_10_-transformed) range size (the average log_10_-transformed range size of species in an assemblage, weighted by the species’ raw untransformed abundance within the assemblage), which we refer to as RCAR; and second, as the simple average of log_10_-transformed range size of species in an assemblage, unweighted by abundance, which we refer to as RAR. The second measure was used to test whether the observed patterns in the abundance-weighted metric were driven by a few very abundant species.

### Sensitivity analyses

In addition to using different measures of range size, we also tested the sensitivity of our main measure (GBIF-based occupancy, on a 110-km grid) to variation in estimated completeness of the underlying GBIF data. To estimate the completeness of range-size estimates for particular taxonomic groups across particular geographic regions, we took grid-based (110 km × 110 km grid) estimates of inventory completeness for trachaeophytes [61], amphibians, mammals, and birds [22]. These inventory completeness estimates indicate the percentage of those species estimated to be present within a grid cell—as inferred from overlaid extent-of-occurrence maps (vertebrates) or a co-kriged environment-richness model (trachaeophytes)—that are represented by GBIF records. The grid-based estimates for each taxonomic group were spatially averaged across broad biogeographical regions (combinations of biogeographic realm and biome). Note that, even with perfect sampling, the completeness estimates based on overlaid range maps are not always expected to reach 100% because, even at the coarse grid resolution used, a species’ area of occupancy will regularly be overestimated by its extent of occurrence [62]. Nevertheless, we anticipate that they will provide a reasonable, broad-scale approximation of the relative completeness of GBIF data.

For each study in our dataset that focused on one of the 4 taxonomic groups with estimates of inventory completeness, we matched each site to the mean inventory completeness of all grid cells within the combination of biogeographical biome and realm where that site’s location is situated. We then constructed models, first based on sites with any nonzero estimate of inventory completeness, and then with successively reduced sets of sites with inventory completeness exceeding 5%, 10%, 15%, 20%, and 25% (this last value was exceeded by only 18% of the sites in our dataset).

For the sensitivity analyses, the reduced dataset for trachaeophytes and vertebrates, and then the successive reductions in the size of the dataset for increasingly stringent estimates of completeness, meant that we had to employ a coarser land-use classification than used for the main results: secondary vegetation in all stages of recovery was combined into a single class, and all human land-use types (plantation forest, cropland, pasture, and urban) were aggregated.

### Human pressure data

We used 5 pressure variables related to human land use and hypothesized as potentially influencing the average range size of species within ecological assemblages: land-use type, land-use intensity, land-use history, human population density, and distance to the nearest road. Land-use type was classified into one of 8 categories: primary vegetation, mature secondary vegetation, intermediate secondary vegetation, young secondary vegetation, plantation forest, cropland, pasture, and urban (see S3 Table and ref. [21] for full definitions). Within each of these classes, each site was assigned a 3-level ordinal measure of intensity, depending on the extent and magnitude of human use of the site (S4 Table and ref. [21]). Criteria for classifying land-use intensity varied according to land-use type. For example, increased intensity in natural (primary and secondary) vegetation could reflect logging or bushmeat hunting, whereas increased intensity in agricultural sites reflected, for example, reduced crop diversity, increased chemical inputs, and increased livestock density (see S4 Table for full criteria).

Land-use history was measured as the number of years since 30% of the land surface within the 5-arc-minute grid cell containing the site became converted to human land uses (cropland, pasture, or urban). We used this threshold because the effects of loss of natural habitat on biodiversity appear to accelerate above 30% [63]. We inferred historic land use from the HYDE model, Version 3.1, at 5-arc-minute spatial resolution [64]. Therefore, the available estimates of land-use history were at much coarser spatial resolution than the local-scale classification of land-use type, which was based on habitat information in the papers that sampled biodiversity. Human population density, at 30-arc-second resolution, was derived from the Global Rural-Urban Mapping Project Version 1 dataset [65]. Distance to the nearest road was calculated from the Global Roads Open Access Data Set Version 1 [66], using the arcpy Python module for ArcMap Version 10.3 [60]: the roads maps was first projected onto an equal-area (Behrmann) projection using the ‘Project_management’ function; then, distance to nearest road (in metres) for each site was calculated using the ‘Near_analysis’ function.

### Statistical analysis

We conducted all statistical analyses in R Version 3.3.2 [67]. Each measure of mean range size (RCAR and RAR) was modelled as a function of the pressure variables using linear mixed-effects models in the lme4 package Version 1.1–14 [68]. We included random intercepts for study identity (to account for among-study differences in sampling methods, sampling effort, measure of abundance used, climatic conditions, and average range-size estimates) and spatial block within study (to account for the spatial structure of sites). It was not feasible to include random slopes in this analysis because of model-convergence issues. Fixed effects considered were each of the 5 pressure variables described above, the two-way interaction between land-use type and land-use intensity, all two-way interactions among the 3 continuous pressure variables (land-use history, human population density, and distance to nearest road), and all two-way interactions between land-use type and each of the continuous pressure variables. Range sizes might show elevational patterns, and it is likely that land use is nonrandom with respect to elevation in some of the surveys. To account for any biases caused by elevational effects, we fitted elevation as an additional fixed effect in the models. The variables describing human population density, distance to nearest road, land-use history, and elevation were log_e_-transformed prior to analysis. The best fixed-effects structure for explaining variation in community-weighted mean range size was obtained by backward stepwise model selection based on likelihood-ratio tests.

To assess whether spatial autocorrelation might bias modelled estimates, we used Moran’s I tests for spatial autocorrelation—implemented in the ‘spdep’ package Version 0.7–4 [69]—on the model residuals associated with each study from which data were obtained. By chance, we expect significant (*P* < 0.05) spatial autocorrelation in the residuals for 5% of studies. To test whether the observed percentage of studies with significant spatial autocorrelation was significantly greater than 5%, we used a one-sided test for equality of proportions, using the ‘prop.test’ function in R. Average range sizes vary geographically for reasons unrelated to land use. This biogeographical variation should be accounted for by the random intercept of study identity in our models, coupled with the fact that most individual studies span only a small geographic area. To check that this was the case, we conducted a spatial analysis of all of the residuals from the main model of RCAR. We tested for an effect of both longitude and latitude (both fitted as cubic polynomials) and their interaction.

To test whether the responses of RCAR and RAR to land use varied geographically or taxonomically, we conducted separate analyses dividing sites between the tropical and temperate realms and dividing species into major taxonomic groups. Tropical sites were defined as those falling between the tropics of Cancer and Capricorn (23.5°N and 23.5°S, respectively), with all other sites considered ‘temperate’ (21 sites fell north of the Arctic Circle at 66.5°N). Taxonomic groups were defined as follows: ‘plants’ for all species in the Kingdom Plantae, ‘invertebrates’ for all annelids, arthropods, and molluscs; and separate divisions for each of the vertebrate Classes, which have more complete range-size estimates (‘amphibians’, ‘birds’, ‘mammals’, and ‘reptiles’).

Any tropical-temperate differences in responses of RCAR and RAR to land use may reflect differences in average climatic variability and topographic heterogeneity between tropical and temperate sites. A previous study using the PREDICTS data showed that turnover in species composition between natural and human land uses is stronger in areas with lower climatic seasonality [7], and other studies have shown that climatic seasonality and topographic heterogeneity are important in determining the range sizes of species [31]. Therefore, we tested whether the strength of the responses of RCAR and RAR to human land use are determined by climatic seasonality and topographic heterogeneity. To do so, we first estimated response strength by fitting mixed-effects models of RCAR and RAR as a function of land-use type, where land-use type was divided simply into natural (primary and secondary vegetation) or human-dominated (plantations, cropland, pasture, and urban sites). As in the main models (see above), we fitted study identity as a random intercept, but in this case also with a random slope of land-use type nested within study identity. These random slopes for human land use were used as the estimate of response strength and were modelled (using simple multiple regression) as a function of the same variables considered in [7]: temperature and precipitation seasonality, derived from WorldClim Version 1.4 [70]; and topographic heterogeneity as the topographic ruggedness index [71], using elevation data from WorldClim Version 1.4 [70]. Both linear and quadratic terms were considered for each of these continuous variables. The simple classification of sites as tropical or temperate was also considered in this analysis but was found to be nonsignificant alongside the more refined variables for both RCAR and RAR (see Results).

To test whether any changes in the average range size of ecological communities (RCAR and RAR) were driven by increases in the abundance of wide-ranging species or by decreases in the abundance of narrow-ranged species (or both), we divided the sampled species within each underlying study—and within taxonomic Classes where studies sampled more than one Class—into 3 equally sized groups based on their range sizes. We then constructed separate generalized linear mixed-effects models of the total (log_e_-transformed) sampled abundance (with a Gaussian error distribution) or species richness (with a Poisson error distribution) of each of these 3 groups as a function of land-use type and land-use intensity. As with the models of RCAR, random intercepts of study identity and spatial block within study were included. We used within-study and within-Class divisions of range size to control, to some extent, for geographic and taxonomic variation in the quality of range-size estimates, and also because the distribution of actual range sizes will vary geographically and taxonomically, and we were interested in whether relatively more widespread species in a given locality respond more positively than more narrow-ranged related species. Only 10% of species in the analysis appear in different range-size categories across studies, and the results were very robust to the exclusion of these species (results not shown).

Spatial homogenization of biodiversity, which will manifest in our models as an increase in the average range size of species within ecological communities, is one possible explanation that reconciles the large observed declines in global biodiversity despite relatively small (or even no) changes in local species richness [5,25]. If this were the case, we would expect species richness changes and changes in average range size not to be strongly coupled. To test this, we fitted a model with only land-use type as a fixed effect, using a coarser classification of land-use type into natural (primary and secondary vegetation) or human (plantation forests, croplands, pastures, and urban habitats). We fitted in this model a random slope of land-use type nested within study identity to obtain an estimate of how strongly average range size responds to human land use. We also fitted a separate model with the same structure but with species richness as a response variable. We then correlated, across studies, the strength of the response of RCAR in human land uses with the strength of the response of species richness.

## Supporting information

S1 Fig Locations of surveys, and taxonomic and geographic representativeness of the data. The location of each survey whose data were included in the analysis (A), shown in the Lambert cylindrical equal area projection. Point diameters are proportional to the (log_e_) number of sites sampled by each survey and are translucent so areas of opaque color indicate overlapping points. The outline map is based on the World Bank map of river basins (https://bit.ly/2J86Kbq), which is published under a CC-BY 4.0 license. The total number of species in the analysed dataset in major taxonomic groups (B), in relation to estimated numbers of described species in the same groups (422). The distribution of sites among terrestrial biomes in relation to the distribution of total terrestrial Net Primary Production (C). The grey line in panel C shows the 1:1 relationship. Letters in this plot indicate the biomes as follows: A, tundra; B, boreal forests and taiga; C, temperate conifer forests; D, temperate broadleaf and mixed forests; E, montane grasslands and shrublands; F, temperate grasslands, savannahs, and shrublands; G, Mediterranean forests, woodlands, and scrub; H, deserts and xeric shrublands; J, tropical and subtropical grasslands, savannahs, and shrublands; K, tropical and subtropical coniferous forests; M, tropical and subtropical dry broadleaf forests; N, tropical and subtropical moist broadleaf forests; P, mangroves. The site-level data underlying this figure are freely available (DOI: 10.6084/m9.figshare.7262732).
(TIF)

S2 Fig Correspondence between estimates of RCAR based on different estimates of species’ range size. Estimates of range occupancy derived from records in the GBIF database were gridded at spatial resolutions of 110 km × 110 km, 55 km × 55 km, and 11 km × 11 km (A–C). A measure of range extent (a conceptually different measure of range size compared with the occupancy measure featured in the main text) was also calculated using GBIF records gridded at the same spatial resolutions (D–F). Finally, we extracted range-size estimates from expert-drawn extent-of-occurrence maps for birds, mammals, and amphibians (G–I), the groups whose ranges are best known. The red lines show 1:1 relationships.
(TIF)

S3 Fig Effects of proximity to roads, human population density, and length of landscape use by humans on RCAR. Separate effects are shown for each land use because interaction terms were significant (all *P* < 0.05). For clarity, shading shows ±0.5 × standard error rather than the 95% confidence interval. Distance to the nearest road is shown here as the raw values rather than the inverse ‘proximity to road’ shown in Fig 1 The site-level data underlying the models shown here are freely available (DOI: 10.6084/m9.figshare.7262732).
(TIF)

S4 Fig Comparison of the effects of human land use on RCAR and on species richness, for individual underlying studies. Separate models were fitted for species richness and RCAR as a function of land use. For these models, land use was classified coarsely as either natural (primary or secondary vegetation) or human (plantation forests, croplands, pastures, and urban environments). A random slope of land use nested within study identity was fitted for each model. The random slope coefficients for human land use were extracted as an estimate of the relative strength of effect of human land use on species richness and RCAR within each individual study. The relationship between the estimates for species richness and RCAR are shown here (black points). A linear model was fitted to test the correspondence, showing a significant but weak negative relationship (R^2^ = 0.014; *P* = 0.008; solid red line = mean fitted relationship; dashed red lines = 95% confidence intervals). The site-level data underlying the models shown here are freely available (DOI: 10.6084/m9.figshare.7262732).
(TIF)

S5 Fig Sensitivity of the estimated effect of land use on RCAR to variation in quality of underlying range-size estimates. Because sample size was much reduced in the most stringent subsets of the data, land use in these models was classified more coarsely than in the main models into primary vegetation, secondary vegetation, and human land uses (combining plantation forests, croplands, pastures, and urban environments). Open triangles show the results based on the complete dataset. Solid triangles of increasing size show results from increasingly stringent subsets of the data, with increasing data quality. Data quality reflected variation in the quality of species’ range-size estimates, and was measured as the estimated inventory completeness of GBIF records for each of 4 taxonomic groups (trachaeophytes, amphibians, mammals, and birds) across different biogeographic regions (combinations of biogeographic realm and biome). Note that inventory completeness is not expected to reach 100% (see Materials and methods, above). Inventory completeness exceeded 25% for only 18% of sites in our dataset. Error bars show 95% confidence intervals. The site-level data underlying the models shown here are freely available (DOI: 10.6084/m9.figshare.7262732).
(TIF)

S6 Fig Sensitivity of the estimated effect of land use on the abundance of widely and narrowly distributed species to variation in quality of underlying range-size estimates. Because sample size was much reduced in the most stringent subsets of the data, land use in these models was classified more coarsely than in the main models, into primary vegetation, secondary vegetation, and human land uses (combining plantation forests, croplands, pastures, and urban environments). Open triangles show the results based on the complete dataset. Solid triangles of increasing size show results from increasingly stringent subsets of the data, with increasing data quality. Data quality reflected variation in the quality of species’ range-size estimates, and was measured as the estimated inventory completeness of GBIF records for each of 4 taxonomic groups (trachaeophytes, amphibians, mammals, and birds) across different biogeographic regions (combinations of biogeographic realm and biome). Note that inventory completeness is not expected to reach 100% (see Materials and methods, above). Inventory completeness exceeded 25% for only 18% of sites in our dataset. Error bars show 95% confidence intervals. The site-level data underlying the models shown here are freely available (DOI: 10.6084/m9.figshare.7262738).
(TIF)

S7 Fig Locations of sites for subsets of the data of increasing stringency in terms of the quality of underlying range-size estimates. Data quality reflected variation in the quality of species’ range-size estimates and was measured as the estimated inventory completeness of GBIF records for each of 4 taxonomic groups (trachaeophytes, amphibians, mammals, and birds) across different biogeographic regions (combinations of biogeographic realm and biome). Note that inventory completeness is not expected to reach 100% (see Materials and methods, above). Inventory completeness exceeded 25% for only 18% of sites in our dataset. The outline maps are based on the World Bank map of river basins (https://bit.ly/2J86Kbq), which is published under a CC-BY 4.0 license. The site-level data underlying the models shown here are freely available (DOI: 10.6084/m9.figshare.7262732).
(TIF)

S8 Fig Effects of land use on RCAR, estimated based on GBIF-based measures of range size gridded at different resolutions. Effects of land use and land-use intensity using RCAR based on range occupancy using GBIF records, gridded at a spatial resolution of 110 km × 110 km (A), 55 km × 55 km (B), and 11 km × 11 km (C). Based on range extent using GBIF records at 110 km × 110 km (D), 55 km × 55 km (E), and 11 km × 11 km (F) resolution. Error bars show 95% confidence intervals. Land use was classified into primary vegetation, mature secondary vegetation, intermediate secondary vegetation, young secondary vegetation, plantation forest, cropland, pasture, and urban. Each land-use class was subdivided into 3 levels of human intensity of use—minimal, light, and intense. The plots show the interaction between land use and land-use intensity, with increasing intensity shown toward the right-hand side for each land use. The site-level data underlying the models shown here are freely available (DOI: 10.6084/m9.figshare.7262732). ISV, intermediate secondary vegetation; MSV, mature secondary vegetation; Plantation, plantation forest; Primary, primary vegetation; YSV, young secondary vegetation.
(TIF)

S9 Fig Spatial autocorrelation in the model residuals. A Moran’s I test was applied to the residuals from the final models, dividing the residuals into the individual underlying surveys. The distribution of *P* values across the tests for each of the surveys is shown here, for measures of RCAR and RAR based on different underlying estimates of range size—range occupancy based on GBIF records (A and B), range extent based on GBIF records (C and D), range occupancy based on GBIF records for vertebrates (E and F), and range extent based on expert-drawn range maps for vertebrates (G and H); and for measures of average range size both weighted (i.e., RCAR; A, C, E, and G) and unweighted (i.e., RAR; B, D, F, and H) by species’ recorded abundances. The red line shows *P* = 0.05. The site-level data underlying the models shown here are freely available (DOI: 10.6084/m9.figshare.7262732).
(TIF)

S10 Fig Spatial patterns in the residuals of the final model of RCAR as a function of human pressures. A map of each site included in the final model, where point colours represent the value of the model residuals for each site (blue = low; red = high), showing no discernible spatial pattern (A). The outline map is based on the World Bank map of river basins (https://bit.ly/2J86Kbq), which is published under a CC-BY 4.0 license. Model residuals for each site as a function of latitude (B). There was no significant relationship between the value of the model residual for a site and either longitude, latitude, or their interaction. The site-level data underlying the models shown here are freely available (DOI: 10.6084/m9.figshare.7262732).
(TIF)

S1 Table Statistics explaining geographical variation in the strength of the response of RCAR to human land use. Linear models were used to explain the strength of the response of RCAR as a function of variables hypothesized to drive observed tropical-temperate differences. Variables considered were geographic zone (tropical versus temperate) itself, and 3 more refined measures of climatic or topographic variability: temperature seasonality, precipitation seasonality, and the topographic ruggedness index. Both linear and quadratic terms were considered for all of the continuous variables (i.e., all variables except geographic zone). Quadratic terms are denoted in this table by a superscript 2. The final model was obtained by backward stepwise model selection, with the significance of terms assessed using analysis of variance. PS, precipitation seasonality; TRI, topographic ruggedness index; TS, temperature seasonality.
(DOCX)

S2 Table Statistics explaining geographical variation in the strength of the response of RAR to human land use. Linear models were used to explain the strength of the response of RAR as a function of variables hypothesized to drive observed tropical-temperate differences. Variables considered were geographic zone (tropical versus temperate) itself, and 3 more refined measures of climatic or topographic variability: temperature seasonality, precipitation seasonality, and the topographic ruggedness index. Both linear and quadratic terms were considered for all of the continuous variables (i.e., all variables except geographic zone). Quadratic terms are denoted in this table by a superscript 2. The final model was obtained by backward stepwise model selection, with the significance of terms assessed using analysis of variance. PS, precipitation seasonality; TRI, topographic ruggedness index; TS, temperature seasonality.
(DOCX)

S3 Table Definitions of the major land-use classes. Each site was classified into one of these classes based on the description of the habitat where the biodiversity sample was taken, as given in the underlying papers from which the biodiversity data were obtained (see S1 Text).
(DOCX)

S4 Table Criteria used to classify land use and land-use intensity. The classification was made based on the description of the habitat given in the underlying papers from which the data were obtained (see S1 Text).
(DOCX)

S1 Text List of all references for underlying community data. These references are a subset of those in the PREDICTS database [21].
(DOCX)

## Abbreviations

CV: coefficient of variation
GBIF: Global Biodiversity Information Facility
H: highest values in the dataset for each continuous effect
PREDICTS: Projecting Responses of Ecological Diversity In Changing Terrestrial Systems
RAR: Relative Average Range Size
RCAR: Relative Community Average Range Size
