# Supplementary figures and images for: Widespread winners and narrow-ranged losers: Land use homogenizes biodiversity in local assemblages worldwide
Source: PLoS Biol. 2018 Dec 4;16(12):e2006841. doi: 10.1371/journal.pbio.2006841 (PMC6279023; doi:10.1371/journal.pbio.2006841)

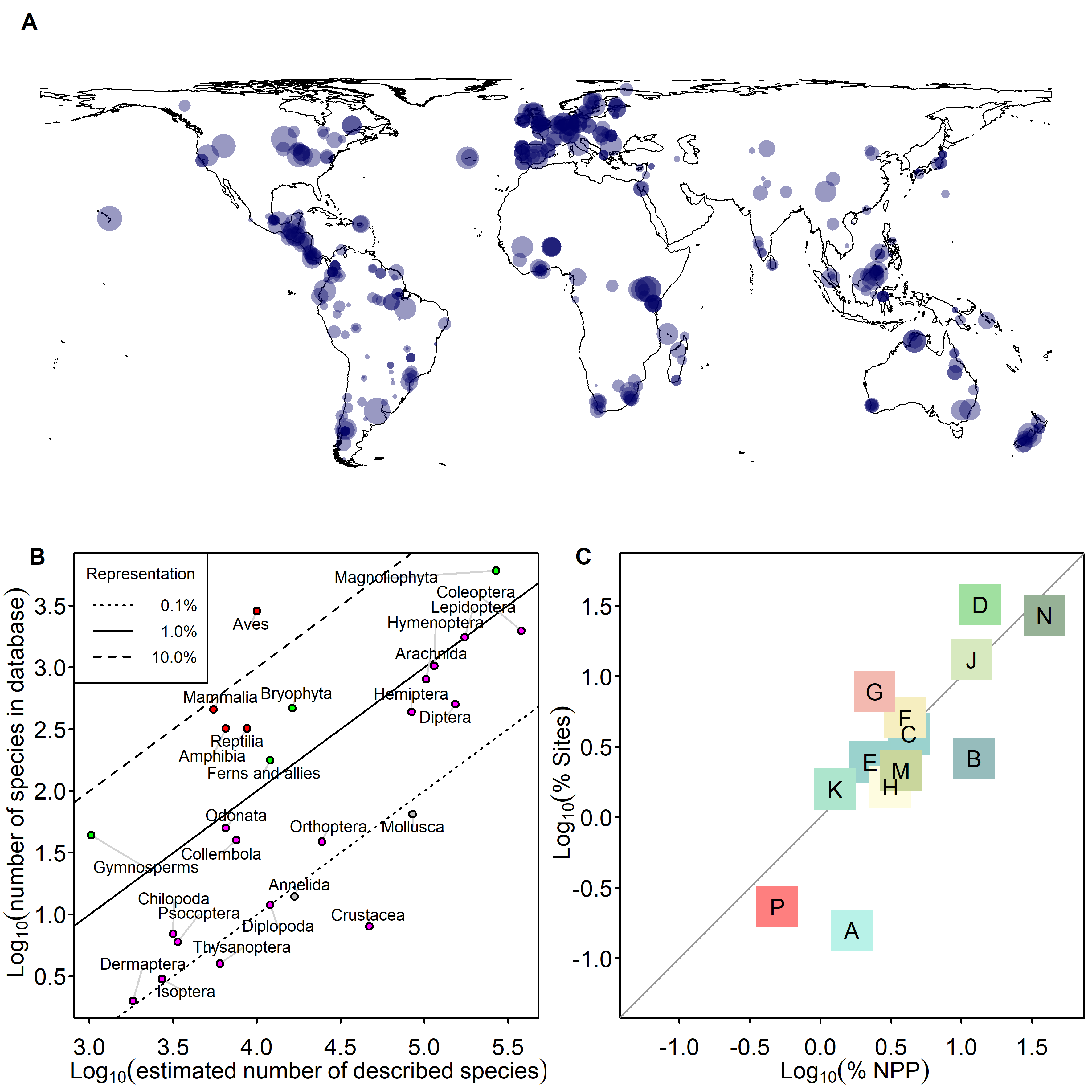

Supplement: S1 Fig — The location of each survey whose data were included in the analysis (A), shown in the Lambert cylindrical equal area projection. Point diameters are proportional to the (loge) number of sites sampled by each survey and are translucent so areas of opaque color indicate overlapping points. The outline map is based on the World Bank map of river basins (https://bit.ly/2J86Kbq), which is published under a CC-BY 4.0 license. The total number of species in the analysed dataset in major taxonomic groups (B), in relation to estimated numbers of described species in the same groups (422). The distribution of sites among terrestrial biomes in relation to the distribution of total terrestrial Net Primary Production (C). The grey line in panel C shows the 1:1 relationship. Letters in this plot indicate the biomes as follows: A, tundra; B, boreal forests and taiga; C, temperate conifer forests; D, temperate broadleaf and mixed forests; E, montane grasslands and shrublands; F, temperate grasslands, savannahs, and shrublands; G, Mediterranean forests, woodlands, and scrub; H, deserts and xeric shrublands; J, tropical and subtropical grasslands, savannahs, and shrublands; K, tropical and subtropical coniferous forests; M, tropical and subtropical dry broadleaf forests; N, tropical and subtropical moist broadleaf forests; P, mangroves. The site-level data underlying this figure are freely available (DOI: 10.6084/m9.figshare.7262732). (TIF) [file pbio.2006841.s001.tif]

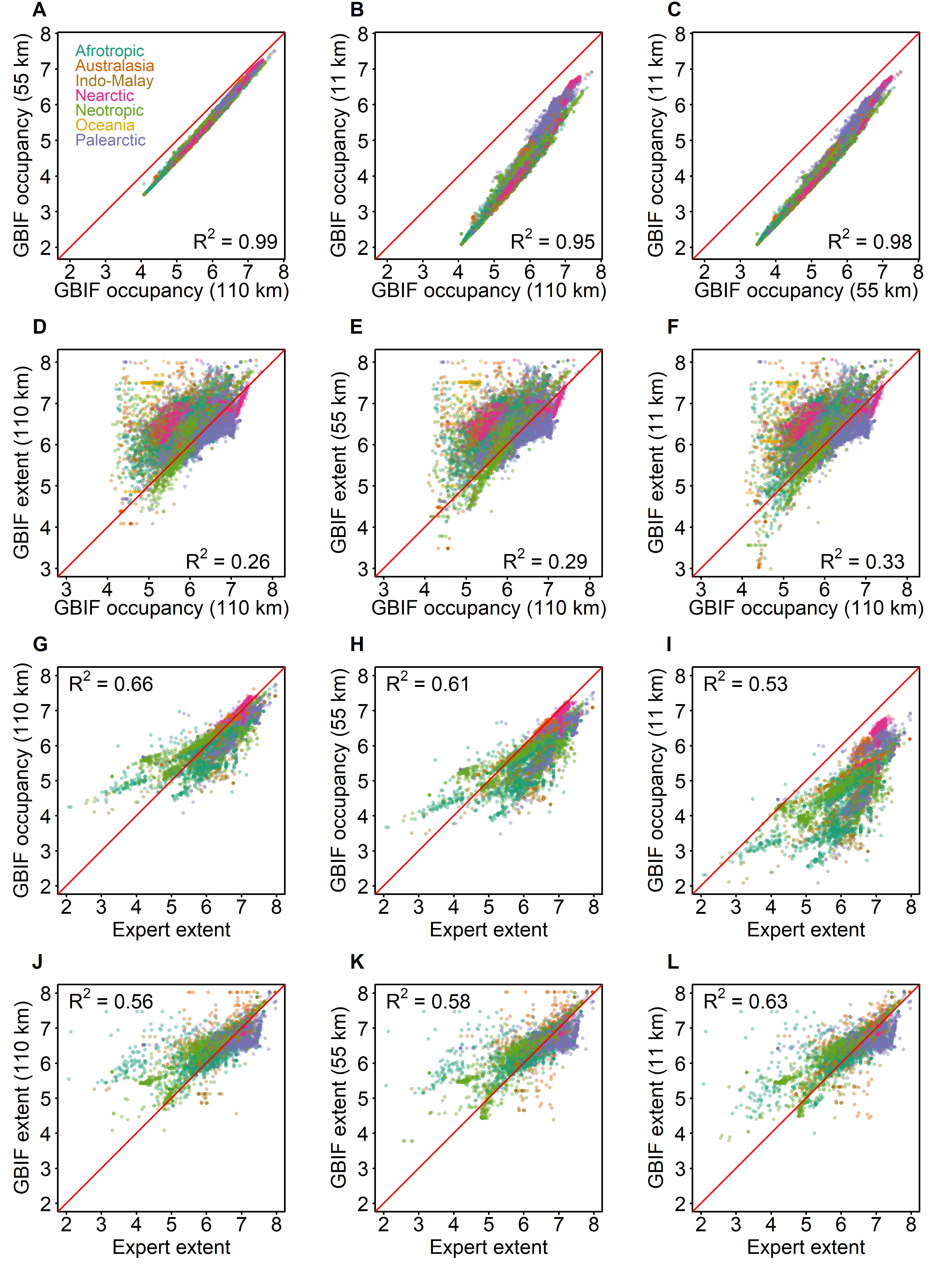

Supplement: S2 Fig — Estimates of range occupancy derived from records in the GBIF database were gridded at spatial resolutions of 110 km × 110 km, 55 km × 55 km, and 11 km × 11 km (A–C). A measure of range extent (a conceptually different measure of range size compared with the occupancy measure featured in the main text) was also calculated using GBIF records gridded at the same spatial resolutions (D–F). Finally, we extracted range-size estimates from expert-drawn extent-of-occurrence maps for birds, mammals, and amphibians (G–I), the groups whose ranges are best known. The red lines show 1:1 relationships. (TIF) [file pbio.2006841.s002.tif]

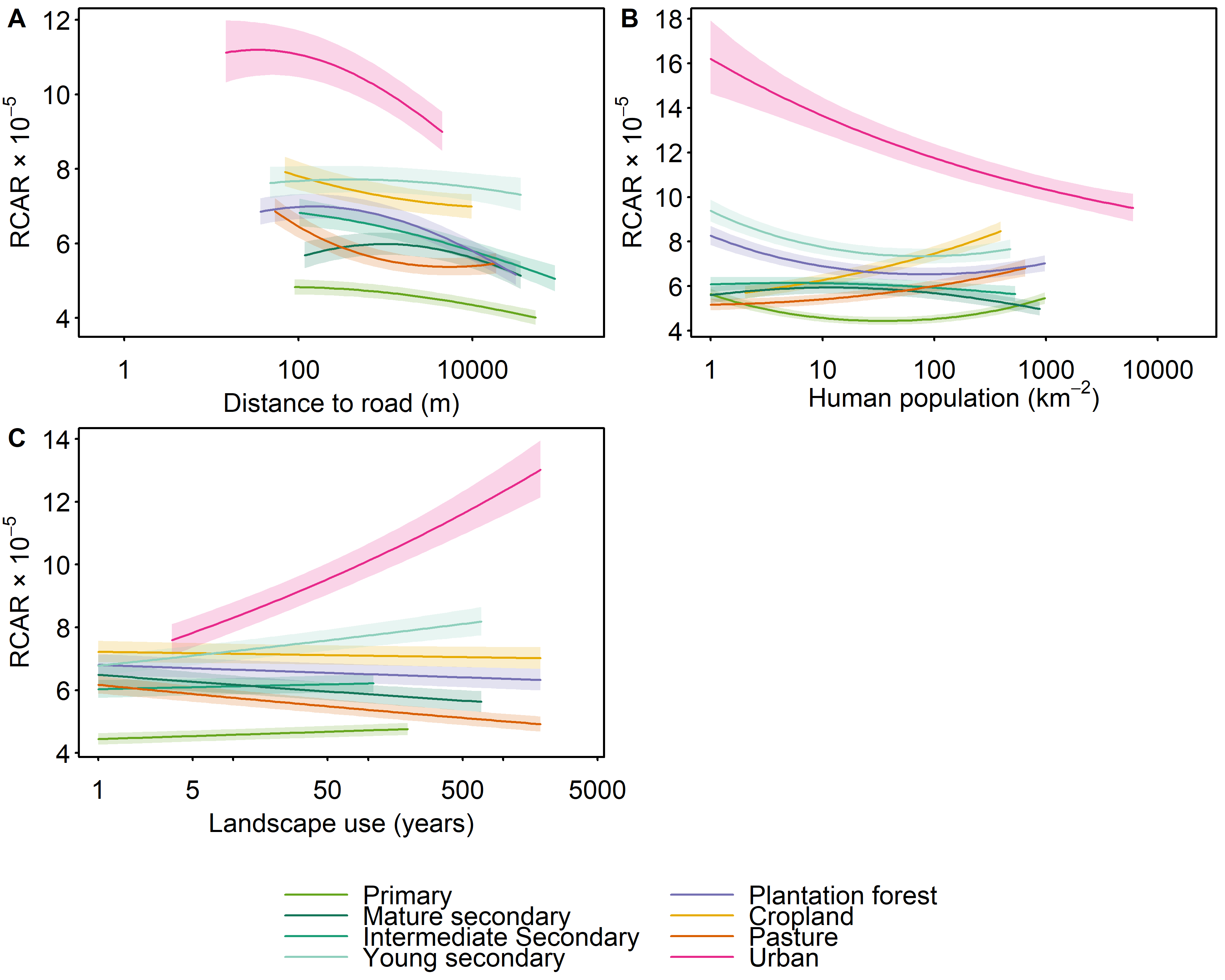

Supplement: S3 Fig — Separate effects are shown for each land use because interaction terms were significant (all P < 0.05). For clarity, shading shows ±0.5 × standard error rather than the 95% confidence interval. Distance to the nearest road is shown here as the raw values rather than the inverse ‘proximity to road’ shown in Fig 1 The site-level data underlying the models shown here are freely available (DOI: 10.6084/m9.figshare.7262732). (TIF) [file pbio.2006841.s003.tif]

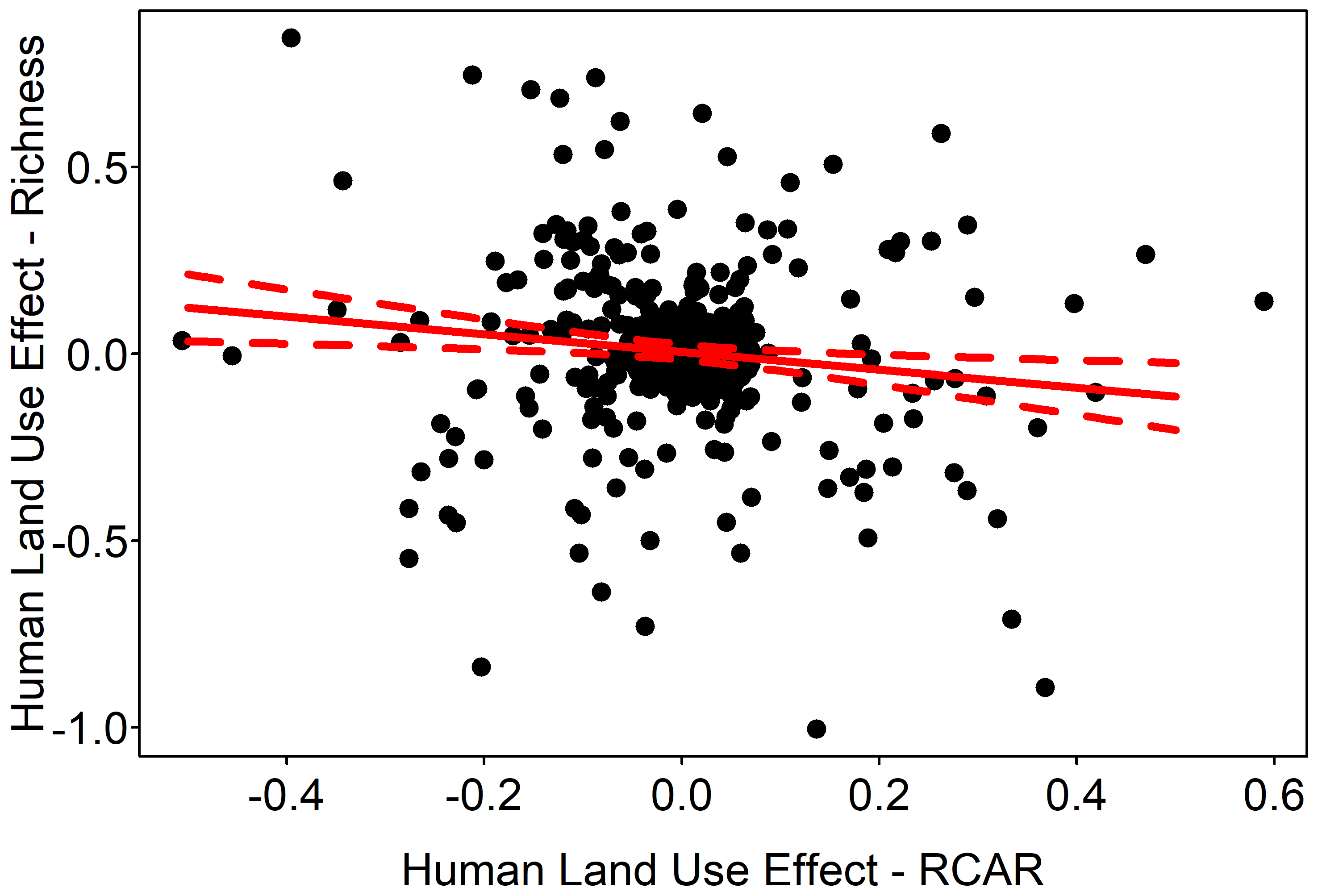

Supplement: S4 Fig — Separate models were fitted for species richness and RCAR as a function of land use. For these models, land use was classified coarsely as either natural (primary or secondary vegetation) or human (plantation forests, croplands, pastures, and urban environments). A random slope of land use nested within study identity was fitted for each model. The random slope coefficients for human land use were extracted as an estimate of the relative strength of effect of human land use on species richness and RCAR within each individual study. The relationship between the estimates for species richness and RCAR are shown here (black points). A linear model was fitted to test the correspondence, showing a significant but weak negative relationship (R2 = 0.014; P = 0.008; solid red line = mean fitted relationship; dashed red lines = 95% confidence intervals). The site-level data underlying the models shown here are freely available (DOI: 10.6084/m9.figshare.7262732). (TIF) [file pbio.2006841.s004.tif]

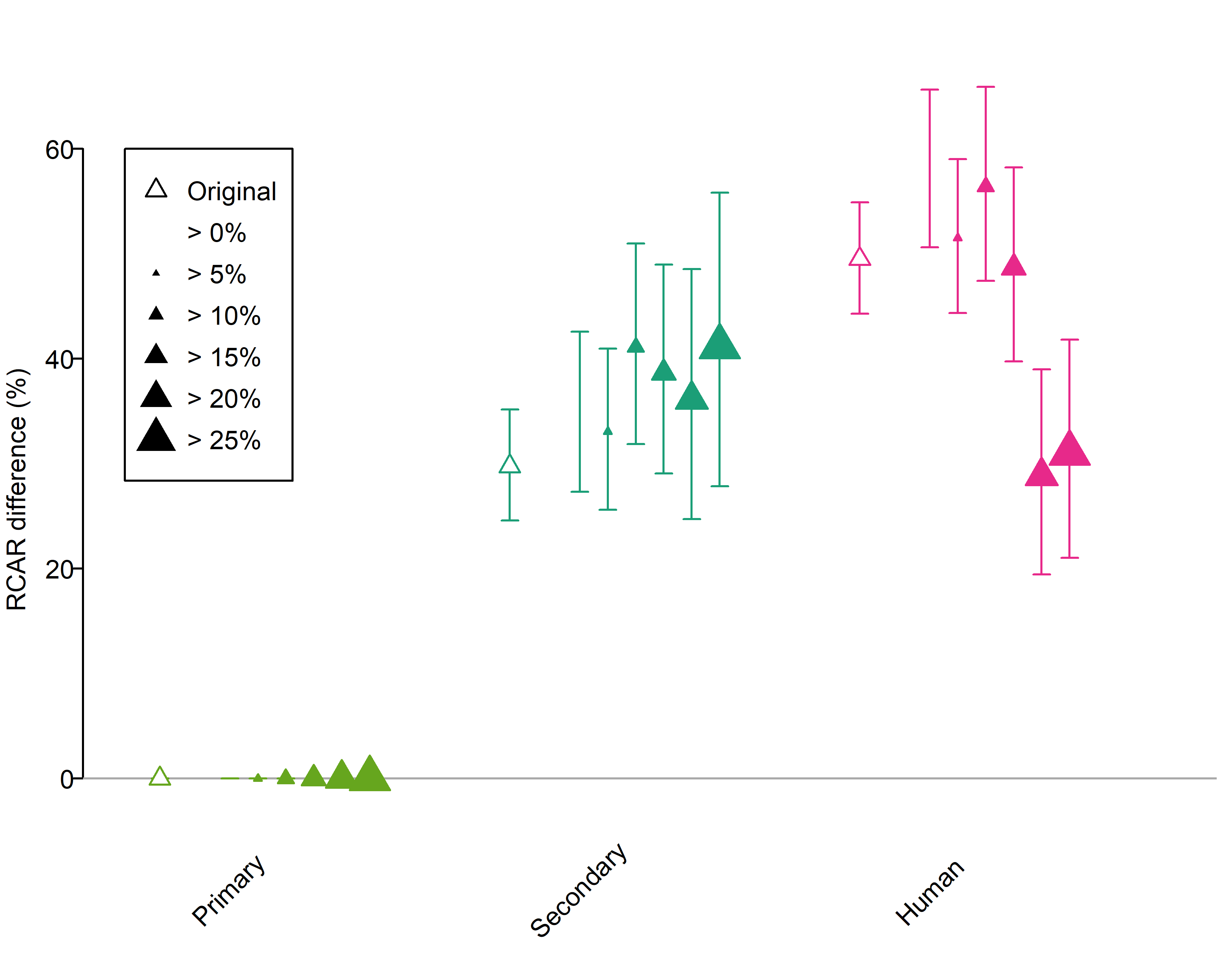

Supplement: S5 Fig — Because sample size was much reduced in the most stringent subsets of the data, land use in these models was classified more coarsely than in the main models into primary vegetation, secondary vegetation, and human land uses (combining plantation forests, croplands, pastures, and urban environments). Open triangles show the results based on the complete dataset. Solid triangles of increasing size show results from increasingly stringent subsets of the data, with increasing data quality. Data quality reflected variation in the quality of species’ range-size estimates, and was measured as the estimated inventory completeness of GBIF records for each of 4 taxonomic groups (trachaeophytes, amphibians, mammals, and birds) across different biogeographic regions (combinations of biogeographic realm and biome). Note that inventory completeness is not expected to reach 100% (see Materials and methods, above). Inventory completeness exceeded 25% for only 18% of sites in our dataset. Error bars show 95% confidence intervals. The site-level data underlying the models shown here are freely available (DOI: 10.6084/m9.figshare.7262732). (TIF) [file pbio.2006841.s005.tif]

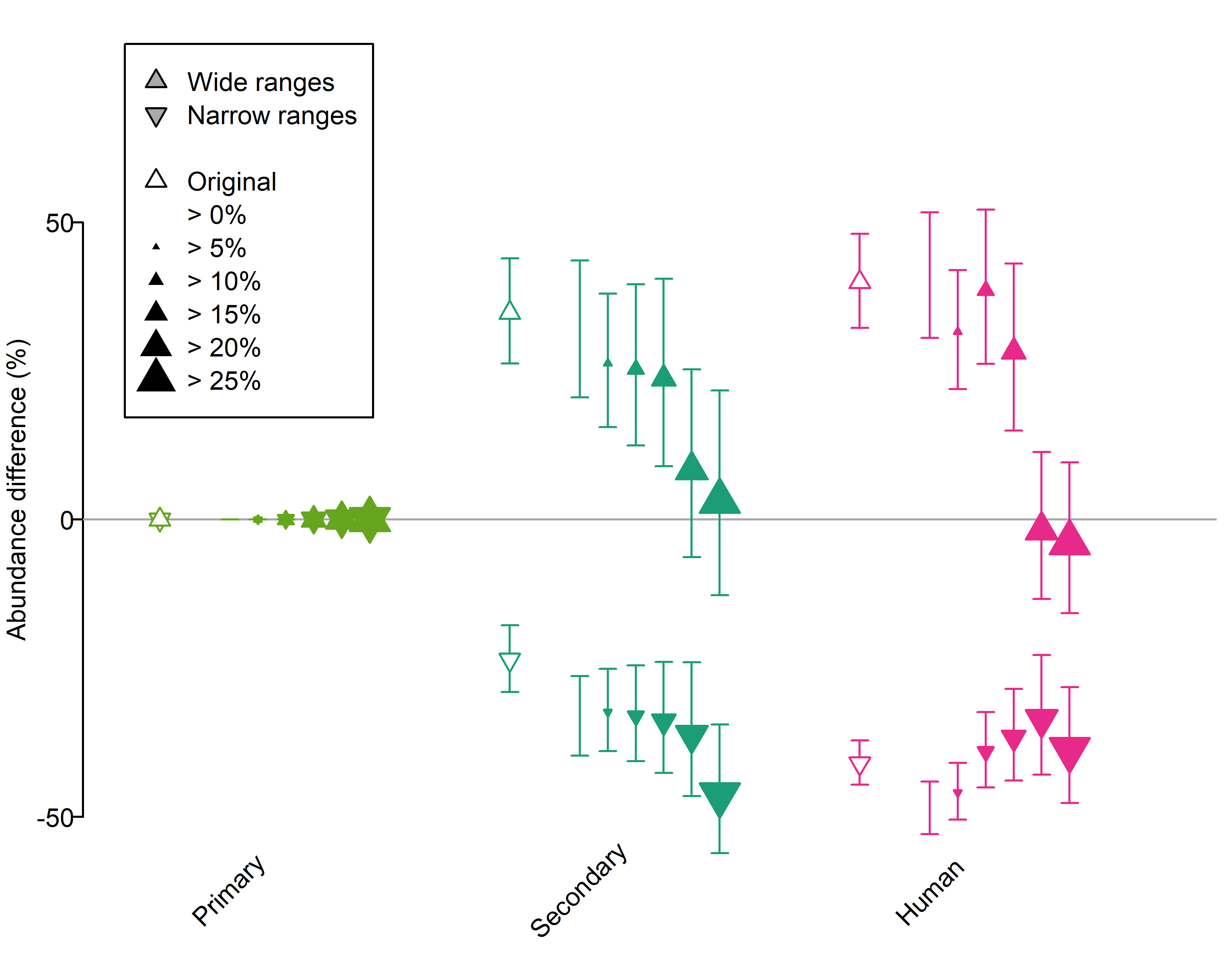

Supplement: S6 Fig — Because sample size was much reduced in the most stringent subsets of the data, land use in these models was classified more coarsely than in the main models, into primary vegetation, secondary vegetation, and human land uses (combining plantation forests, croplands, pastures, and urban environments). Open triangles show the results based on the complete dataset. Solid triangles of increasing size show results from increasingly stringent subsets of the data, with increasing data quality. Data quality reflected variation in the quality of species’ range-size estimates, and was measured as the estimated inventory completeness of GBIF records for each of 4 taxonomic groups (trachaeophytes, amphibians, mammals, and birds) across different biogeographic regions (combinations of biogeographic realm and biome). Note that inventory completeness is not expected to reach 100% (see Materials and methods, above). Inventory completeness exceeded 25% for only 18% of sites in our dataset. Error bars show 95% confidence intervals. The site-level data underlying the models shown here are freely available (DOI: 10.6084/m9.figshare.7262738). (TIF) [file pbio.2006841.s006.tif]

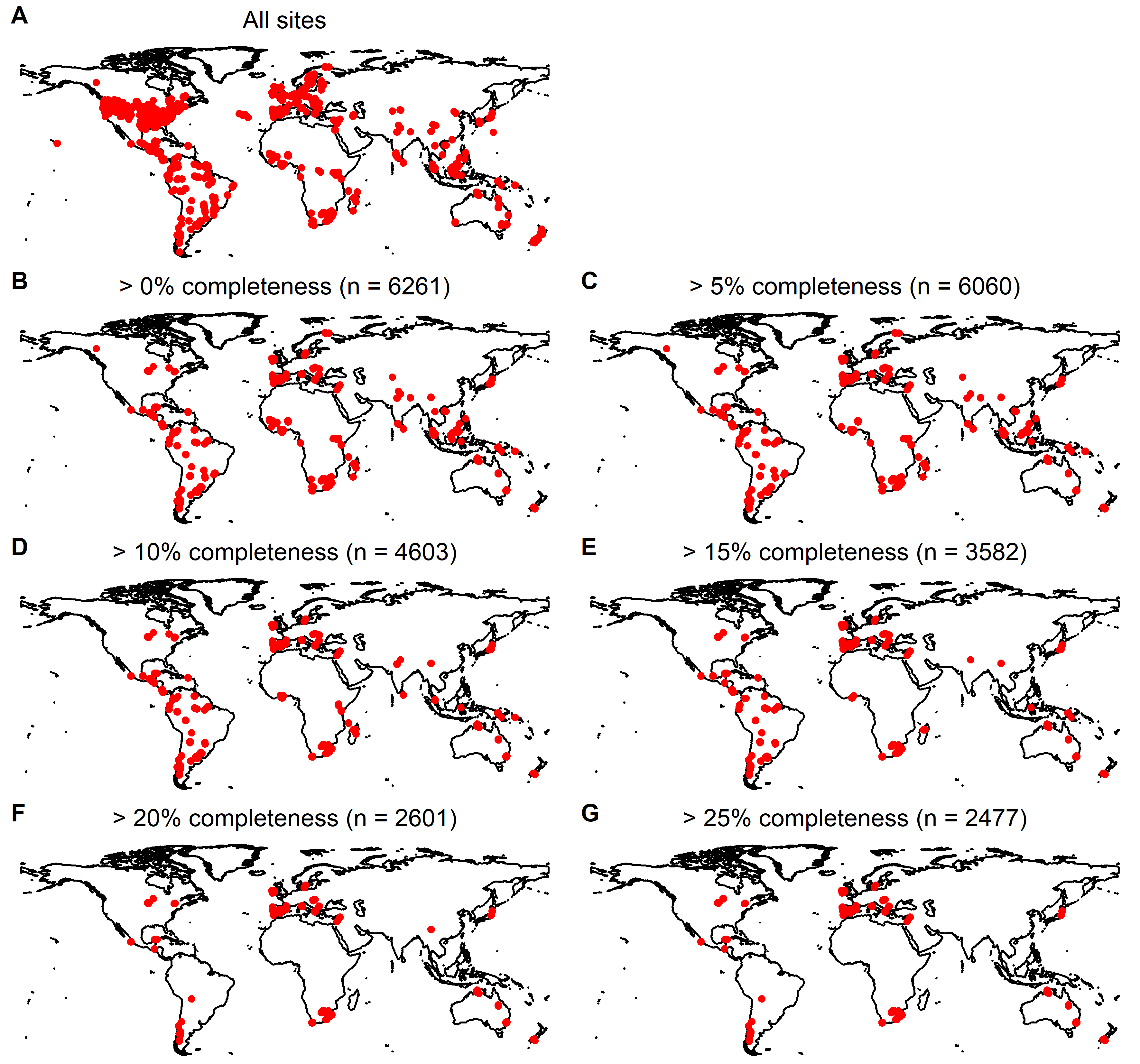

Supplement: S7 Fig — Data quality reflected variation in the quality of species’ range-size estimates and was measured as the estimated inventory completeness of GBIF records for each of 4 taxonomic groups (trachaeophytes, amphibians, mammals, and birds) across different biogeographic regions (combinations of biogeographic realm and biome). Note that inventory completeness is not expected to reach 100% (see Materials and methods, above). Inventory completeness exceeded 25% for only 18% of sites in our dataset. The outline maps are based on the World Bank map of river basins (https://bit.ly/2J86Kbq), which is published under a CC-BY 4.0 license. The site-level data underlying the models shown here are freely available (DOI: 10.6084/m9.figshare.7262732). (TIF) [file pbio.2006841.s007.tif]

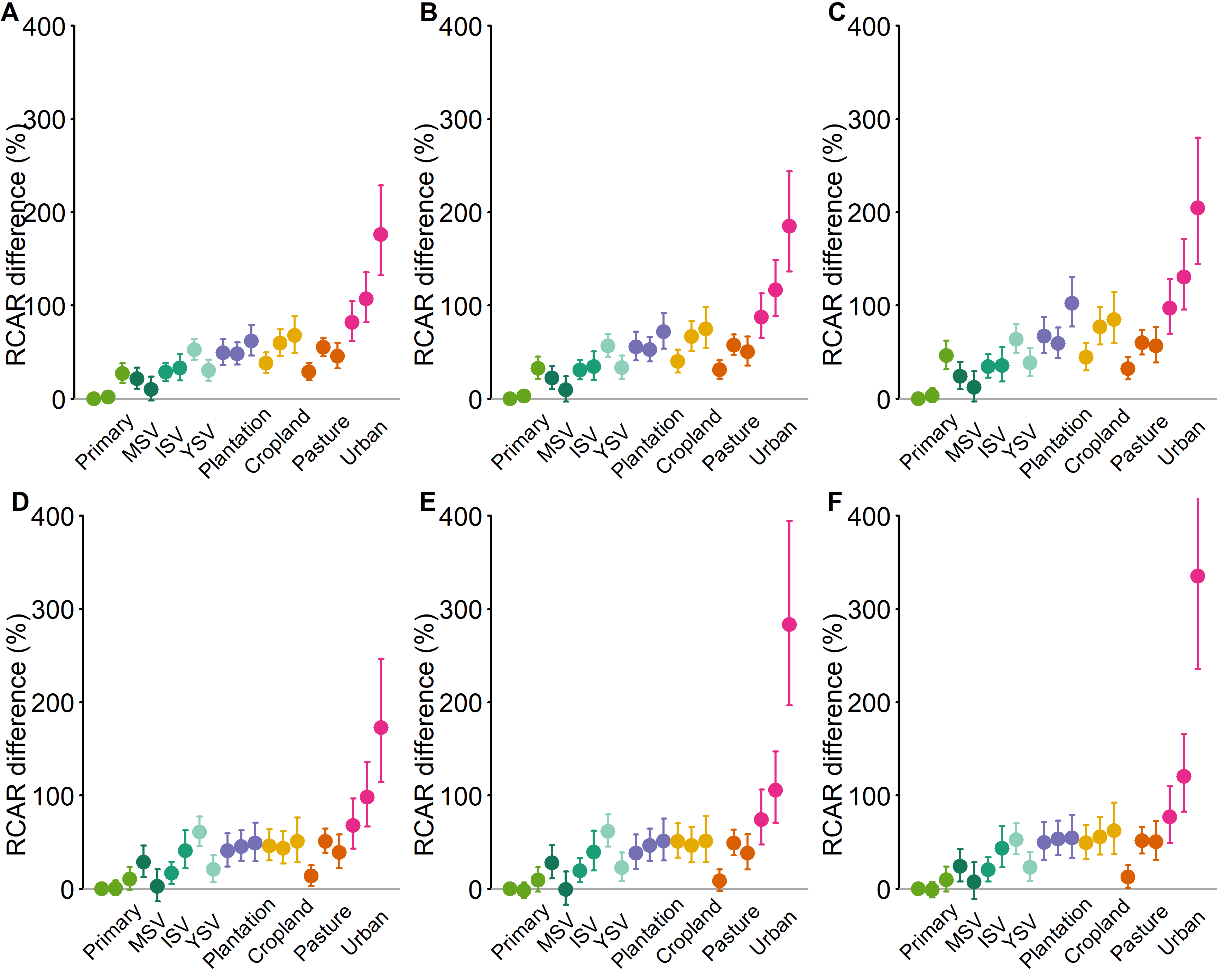

Supplement: S8 Fig — Effects of land use and land-use intensity using RCAR based on range occupancy using GBIF records, gridded at a spatial resolution of 110 km × 110 km (A), 55 km × 55 km (B), and 11 km × 11 km (C). Based on range extent using GBIF records at 110 km × 110 km (D), 55 km × 55 km (E), and 11 km × 11 km (F) resolution. Error bars show 95% confidence intervals. Land use was classified into primary vegetation, mature secondary vegetation, intermediate secondary vegetation, young secondary vegetation, plantation forest, cropland, pasture, and urban. Each land-use class was subdivided into 3 levels of human intensity of use—minimal, light, and intense. The plots show the interaction between land use and land-use intensity, with increasing intensity shown toward the right-hand side for each land use. The site-level data underlying the models shown here are freely available (DOI: 10.6084/m9.figshare.7262732). ISV, intermediate secondary vegetation; MSV, mature secondary vegetation; Plantation, plantation forest; Primary, primary vegetation; YSV, young secondary vegetation. (TIF) [file pbio.2006841.s008.tif]

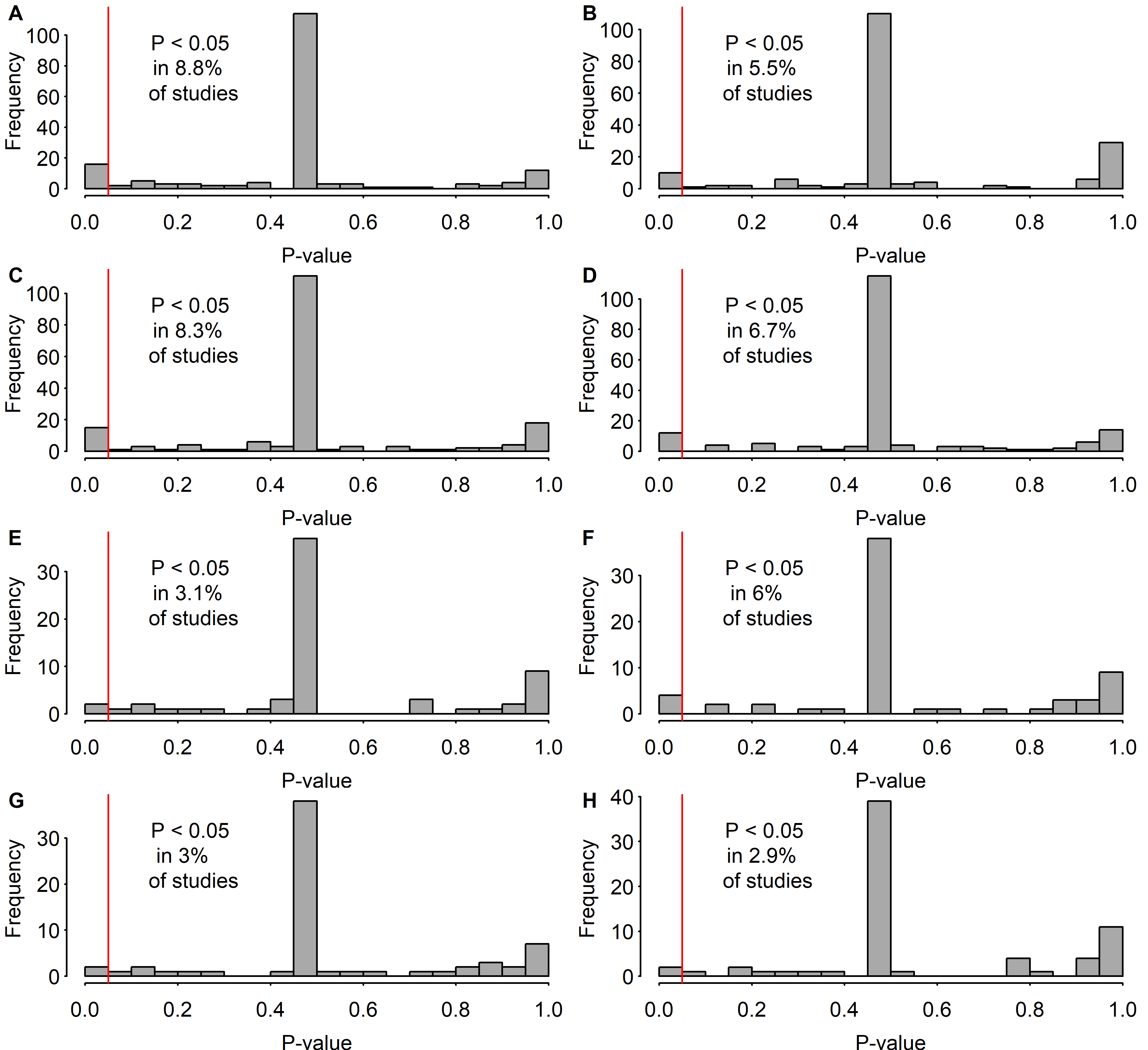

Supplement: S9 Fig — A Moran’s I test was applied to the residuals from the final models, dividing the residuals into the individual underlying surveys. The distribution of P values across the tests for each of the surveys is shown here, for measures of RCAR and RAR based on different underlying estimates of range size—range occupancy based on GBIF records (A and B), range extent based on GBIF records (C and D), range occupancy based on GBIF records for vertebrates (E and F), and range extent based on expert-drawn range maps for vertebrates (G and H); and for measures of average range size both weighted (i.e., RCAR; A, C, E, and G) and unweighted (i.e., RAR; B, D, F, and H) by species’ recorded abundances. The red line shows P = 0.05. The site-level data underlying the models shown here are freely available (DOI: 10.6084/m9.figshare.7262732). (TIF) [file pbio.2006841.s009.tif]

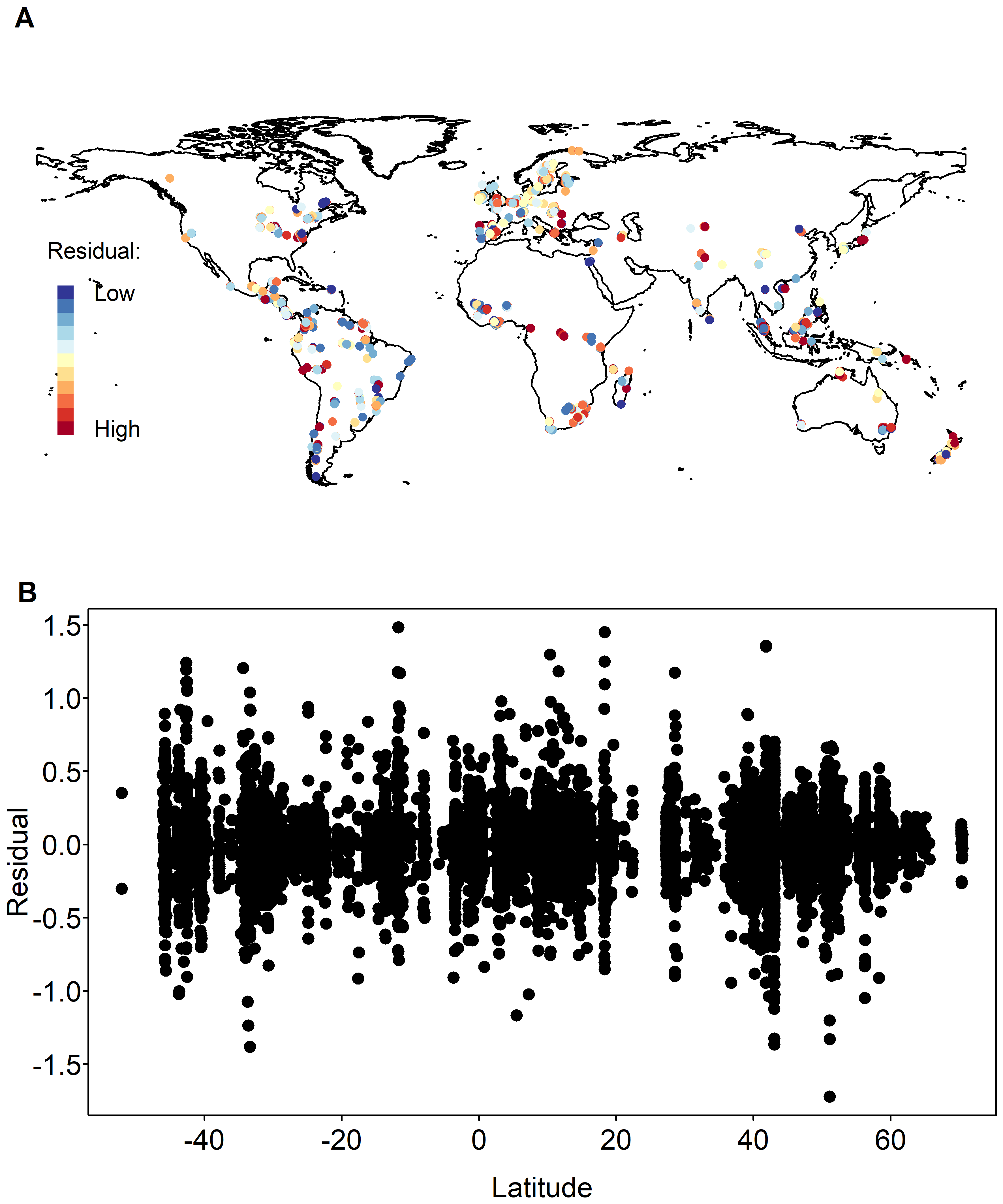

Supplement: S10 Fig — A map of each site included in the final model, where point colours represent the value of the model residuals for each site (blue = low; red = high), showing no discernible spatial pattern (A). The outline map is based on the World Bank map of river basins (https://bit.ly/2J86Kbq), which is published under a CC-BY 4.0 license. Model residuals for each site as a function of latitude (B). There was no significant relationship between the value of the model residual for a site and either longitude, latitude, or their interaction. The site-level data underlying the models shown here are freely available (DOI: 10.6084/m9.figshare.7262732). (TIF) [file pbio.2006841.s010.tif]
