# Supplementary material for: Widespread winners and narrow-ranged losers: Land use homogenizes biodiversity in local assemblages worldwide
Source: PLoS Biol. 2018 Dec 4;16(12):e2006841. doi: 10.1371/journal.pbio.2006841 (PMC6279023; doi:10.1371/journal.pbio.2006841)
Supplement: S2 Table — Linear models were used to explain the strength of the response of RAR as a function of variables hypothesized to drive observed tropical-temperate differences. Variables considered were geographic zone (tropical versus temperate) itself, and 3 more refined measures of climatic or topographic variability: temperature seasonality, precipitation seasonality, and the topographic ruggedness index. Both linear and quadratic terms were considered for all of the continuous variables (i.e., all variables except geographic zone). Quadratic terms are denoted in this table by a superscript 2. The final model was obtained by backward stepwise model selection, with the significance of terms assessed using analysis of variance. PS, precipitation seasonality; TRI, topographic ruggedness index; TS, temperature seasonality. (DOCX) [file pbio.2006841.s012.docx]

| **Explanatory variable** | **F** | **P** |
| --- | --- | --- |
| Geographic zone | 0.39 | 0.53 |
| TS | 2.18 | 0.14 |
| TS^2^ | 2.17 | 0.14 |
| PS | 11.16 | 0.0009 |
| PS^2^ | 0.0003 | 0.99 |
| TRI | 2.04 | 0.15 |
| TRI^2^ | 0.52 | 0.47 |
