# Supplementary material for: Widespread winners and narrow-ranged losers: Land use homogenizes biodiversity in local assemblages worldwide
Source: PLoS Biol. 2018 Dec 4;16(12):e2006841. doi: 10.1371/journal.pbio.2006841 (PMC6279023; doi:10.1371/journal.pbio.2006841)
Supplement: S3 Table — Each site was classified into one of these classes based on the description of the habitat where the biodiversity sample was taken, as given in the underlying papers from which the biodiversity data were obtained (see S1 Text). (DOCX) [file pbio.2006841.s013.docx]

| **Land use** | **Definition** |
| --- | --- |
| Primary vegetation | Native vegetation that is not known or inferred to have ever been completely destroyed, before the year in which the biodiversity was sampled, by human actions or by extreme natural events that do not normally play a role in ecosystem dynamics. |
| Secondary vegetation | Areas where the original primary vegetation was completely destroyed prior to biodiversity sampling. This could have been by human actions (including fire), and includes where sites are recovering to a natural state following a period of human-dominated land use (cropland, plantation forest, pasture or urban). Also counted as secondary are places where natural events (fires, storms etc.) have destroyed the vegetation, but not where the vegetation is naturally maintained by fire (such as climatically Mediterranean systems), which would be primary. Secondary vegetation includes areas where humans have made an active attempt (through planting etc.) to return an area where the natural vegetation was previously destroyed to a more natural state. |
| Plantation forest | Previously cleared areas that people have planted with crop trees or crop shrubs for commercial or subsistence harvesting of wood and/or fruit. |
| Cropland | Land that people have planted with herbaceous crops, even if these crops will be fed to livestock once harvested |
| Pasture | Land where livestock is known to be grazed regularly or permanently |
| Urban | Areas with human habitation and/or buildings, where the primary vegetation has been removed, and where such vegetation as is present is predominantly managed for civic or personal amenity. |
