# Supplementary material for: Widespread winners and narrow-ranged losers: Land use homogenizes biodiversity in local assemblages worldwide
Source: PLoS Biol. 2018 Dec 4;16(12):e2006841. doi: 10.1371/journal.pbio.2006841 (PMC6279023; doi:10.1371/journal.pbio.2006841)
Supplement: S4 Table — The classification was made based on the description of the habitat given in the underlying papers from which the data were obtained (see S1 Text). (DOCX) [file pbio.2006841.s014.docx]

| **Level 1 Land Use** | **Predominant Land Use** | **Minimal use** | **Light use** | **Intense use** |
| --- | --- | --- | --- | --- |
| **No evidence of prior destruction of the vegetation** | **Primary vegetation** | Any disturbances identified are very minor (e.g., a trail or path) or very limited in the scope of their effect (e.g., hunting of a particular species of limited ecological importance). | One or more disturbances of moderate intensity (e.g., selective logging) or breadth of impact (e.g., bushmeat extraction), which are not severe enough to markedly change the nature of the ecosystem. Primary sites in suburban settings are at least Light use. | One or more disturbances that is severe enough to markedly change the nature of the ecosystem; this includes clear-felling of part of the site too recently for much recovery to have occurred. Primary sites in fully urban settings should be classed as Intense use. |
| **Recovering after destruction of the vegetation** | **Mature Secondary Vegetation** | As for Primary Vegetation-Minimal use | As for Primary Vegetation-Light use | As for Primary Vegetation-Intense use |
|  | **Intermediate Secondary Vegetation** | As for Primary Vegetation-Minimal use | As for Primary Vegetation-Light use | As for Primary Vegetation-Intense use |
|  | **Young Secondary Vegetation** | As for Primary Vegetation-Minimal use | As for Primary Vegetation-Light use | As for Primary Vegetation-Intense use |
|  | **Secondary Vegetation (indeterminate age)** | As for Primary Vegetation-Minimal use | As for Primary Vegetation-Light use | As for Primary Vegetation-Intense use |
| **Human use (agricultural)** | **Plantation forest** | Extensively managed or mixed timber, fruit/coffee, oil-palm or rubber plantations in which native understorey and/or other native tree species are tolerated, which are not treated with pesticide or fertiliser, and which have not been recently (< 20 years) clear-felled. | Monoculture fruit/coffee/rubber plantations with limited pesticide input, or mixed species plantations with significant inputs. Monoculture timber plantations of mixed age with no recent (< 20 years) clear-felling. Monoculture oil-palm plantations with no recent (< 20 years) clear-felling. | Monoculture fruit/coffee/rubber plantations with significant pesticide input.  Monoculture timber plantations with similarly aged trees or timber/oil-palm plantations with extensive recent (< 20 years) clear-felling. |
| **Human use (agricultural)** | **Cropland** | Low-intensity farms, typically with small fields, mixed crops, crop rotation, little or no inorganic fertiliser use, little or no pesticide use, little or no ploughing, little or no irrigation, little or no mechanisation. | Medium intensity farming, typically showing some but not many of the following: large fields, annual ploughing, inorganic fertiliser application, pesticide application, irrigation, no crop rotation, mechanisation, monoculture crop. Organic farms in developed countries often fall within this category, as may high-intensity farming in developing countries. | High-intensity monoculture farming, typically showing many of the following features: large fields, annual ploughing, inorganic fertiliser application, pesticide application, irrigation, mechanisation, no crop rotation. |
|  | **Pasture** | Pasture with minimal input of fertiliser and pesticide, and with low stock density (*not* high enough to cause significant disturbance or to stop regeneration of vegetation). | Pasture either with significant input of fertiliser or pesticide, or with high stock density (high enough to cause significant disturbance or to stop regeneration of vegetation). | Pasture with significant input of fertiliser or pesticide, *and* with high stock density (high enough to cause significant disturbance or to stop regeneration of vegetation). |
| **Human use (urban)** | **Urban** | Extensive managed green spaces; villages. | Suburban (e.g. gardens), or small managed or unmanaged green spaces in cities. | Fully urban with no significant green spaces. |
