# Supplementary material for: Widespread winners and narrow-ranged losers: Land use homogenizes biodiversity in local assemblages worldwide
Source: PLoS Biol. 2018 Dec 4;16(12):e2006841. doi: 10.1371/journal.pbio.2006841 (PMC6279023; doi:10.1371/journal.pbio.2006841)
Supplement: S1 Text — These references are a subset of those in the PREDICTS database [21]. (DOCX) [file pbio.2006841.s015.docx]

# References to PREDICTS Data Used

1. Phalan B, Onial M, Balmford A, Green RE. Reconciling food production and biodiversity conservation: land sharing and land sparing compared. Science 2011; 333: 1289–1291. doi: 10.1126/science.1208742

2. Gardner TA, Hernández MIM, Barlow J, Peres CA. Understanding the biodiversity consequences of habitat change: the value of secondary and plantation forests for neotropical dung beetles. J Appl Ecol 2008; 45: 883–893. doi: 10.1111/j.1365-2664.2008.01454.x

3. Centre for International Forestry Research. Multidisciplinary Landscape Assessment ‒ Cameroon. 2013. http://www.cifor.org/mla/_ref/method/index.htm

4. Centro Agronómico Tropical de Investigación y Enseñanza. Unpublished data of reptilian and amphibian diversity in six countries in Central America. 2010.

5. Saldaña-Vázquez RA, Sosa VJ, Hernández-Montero JR, López-Barrera F. Abundance responses of frugivorous bats (Stenodermatinae) to coffee cultivation and selective logging practices in mountainous central Veracruz, Mexico. Biodivers Conserv 2010; 19: 2111–2124. doi: 10.1007/s10531-010-9829-6

6. Center for International Forestry Research. Multidisciplinary Landscape Assessment ‒ Philippines. 2013. http://www.cifor.org/mla/_ref/method/index.htm

7. García-R JC, Cárdenas-H H, Castro-H F. Relationship between anurans diversity and successional stages of a very humid low montane forest in Valle del Cauca, southwestern of Colombsia. Caldasia 2007; 29: 363–374.

8. Presley SJ, Willig MR, Wunderle Jr. JM, Saldanha LN. Effects of reduced-impact logging and forest physiognomy on bat populations of lowland Amazonian forest. J Appl Ecol 2008; 45: 14–25. doi: 10.1111/j.1365-2664.2007.01373.x

9. Castro H, Lehsten V, Lavorel S, Freitas H. Functional response traits in relation to land use change in the Montado. Agric Ecosyst Environ 2010; 137: 183–191. doi: 10.1016/j.agee.2010.02.002

10. Willig MR, Presley SJ, Bloch CP, Hice CL, Yanoviak SP, Diaz MM, et al. Phyllostomid bats of lowland Amazonia: effects of habitat alteration on abundance. Biotropica 2007; 39: 737–746. doi: 10.1111/j.1744-7429.2007.00322.x

11. Uehara-Prado M, Brown Jr. KS, Lucci Freitas AV. Species richness, composition and abundance of fruit-feeding butterflies in the Brazilian Atlantic Forest: comparison between a fragmented and a continuous landscape. Glob Ecol Biogeogr 2007; 16: 43–54. doi: 10.1111/j.1466-822x.2006.00267.x

12. Katovai E, Burley AL, Mayfield MM. Understory plant species and functional diversity in the degraded wet tropical forests of Kolombangara Island, Solomon Islands. Biol Conserv 2012; 145: 214–224. doi: 10.1016/j.biocon.2011.11.008

13. García KP, Ortiz Zapata JC, Aguayo M, D’Elia G. Assessing rodent community responses in disturbed environments of the Chilean Patagonia. Mammalia 2013; 77: 195–204. doi: 10.1515/mammalia-2011-0134

14. Proença VM, Pereira HM, Guilherme J, Vicente L. Plant and bird diversity in natural forests and in native and exotic plantations in NW Portugal. Acta Oecologica 2010; 36: 219–226. doi: 10.1016/j.actao.2010.01.002

15. Carvalho AL de, Ferreira EJL, Lima JMT, de Carvalho AL. Floristic and structural comparisons among palm communities in primary and secondary forest fragments of the Raimundo Irineu Serra Environmental Protection Area - Rio Branco, Acre, Brazil. 2010; 40: 657–666. doi: 10.1590/s0044-59672010000400004

16. Gaigher R, Samways MJ. Surface-active arthropods in organic vineyards, integrated vineyards and natural habitat in the Cape Floristic Region. J Insect Conserv 2010; 14: 595–605.

17. Naidoo R. Species richness and community composition of songbirds in a tropical forest-agricultural landscape. Anim Conserv 2004; 7: 93–105.

18. Zhang JN, Li Q, Liang WJ. Effect of acetochlor and carbofuran on soil nematode communities in a Chinese soybean field. Afr J Agric Res 2010; 5: 2787–2794.

19. Smith-Pardo A, Gonzalez VH. Diversidad de abejas (Hymenoptera: Apoidea) en estados sucesionales del bosque humedo tropical. Acta Biológica Colomb 2007; 12: 43–55.

20. Verdú JR, Moreno CE, Sánchez-Rojas G, Numa C, Galante E, Halffter G. Grazing promotes dung beetle diversity in the xeric landscape of a Mexican Biosphere Reserve. Biol Conserv 2007; 140: 308–317.

21. Tonietto R, Fant J, Ascher J, Ellis K, Larkin D. A comparison of bee communities of Chicago green roofs, parks and prairies. Landsc Urban Plan 2011; 103: 102–108. doi: 10.1016/j.landurbplan.2011.07.004

22. Yamaura Y, Royle JA, Shimada N, Asanuma S, Sato T, Taki H, et al. Biodiversity of man-made open habitats in an underused country: a class of multispecies abundance models for count data. Biodivers Conserv 2012; 21: 1365–1380. doi: 10.1007/s10531-012-0244-z

23. Berg A, Ahrne K, Ockinger E, Svensson R, Soderstrom B. Butterfly distribution and abundance is affected by variation in the Swedish forest-farmland landscape. Biol Conserv 2011; 144: 2819–2831. doi: 10.1016/j.biocon.2011.07.035

24. Rey-Velasco JC, Miranda-Esquivel DR. Unpublished data of the response of ground beetles (Coleoptera: Carabidae) in the northeastern Colombian Andes to habitat modification. 2012.

25. Littlewood NA. Grazing impacts on moth diversity and abundance on a Scottish upland estate. Insect Conserv Divers 2008; 1: 151–160. doi: 10.1111/j.1752-4598.2008.00021.x

26. Gaublomme E, Hendrickx F, Dhuyvetter H, Desender K. The effects of forest patch size and matrix type on changes in carabid beetle assemblages in an urbanized landscape. Biol Conserv 2008; 141: 2585–2596. doi: 10.1016/j.biocon.2008.07.022

27. Wronski T, Gilbert K, Long E, Micha B, Quinn R, Hausdorf B. Species richness and meta-community structure of land snails along an altitudinal gradient on Bioko Island, Equatorial Guinea. J Molluscan Stud 2014; 80: 161–168. doi: 10.1093/mollus/eyu008

28. Clark RJ, Gerard PJ, Mellsop JM. Spider biodiversity and density following cultivation in pastures in the Waikato, New Zealand. New Zeal J Agric Res 2004; 47: 247–259. doi: 10.1080/00288233.2004.9513592

29. Vanbergen AJ, Woodcock BA, Watt AD, Niemela J. Effect of land-use heterogeneity on carabid communities at the landscape scale. Ecography 2005; 28: 3–16. doi: 10.1111/j.0906-7590.2005.03991.x

30. Miranda MV, Politi N, Rivera LO. Unexpected changes in the bird assemblage in areas under selective logging in piedmont forest in northwestern Argentina. Ornitol Neotrop 2010; 21: 323–337.

31. Aben J, Dorenbosch M, Herzog SK, Smolders AJP, Van Der Velde G. Human disturbance affects a deciduous forest bird community in the Andean foothills of central Bolivia. Bird Conserv Int 2008; 18: 363–380. doi: 10.1017/s0959270908007326

32. Parra-H A, Nates-Parra G. Variation of the orchid bees community (Hymenoptera : Apidae) in three altered habitats of the Colombian “llano” piedmont. Rev Biol Trop 2007; 55: 931–941.

33. Littlewood NA, Pakeman RJ, Pozsgai G. Grazing impacts on Auchenorrhyncha diversity and abundance on a Scottish upland estate. Insect Conserv Divers 2012; 5: 67–74. doi: 10.1111/j.1752-4598.2011.00135.x

34. Hylander K, Weibull H. Do time-lagged extinctions and colonizations change the interpretation of buffer strip effectiveness? A study of riparian bryophytes in the first decade after logging. J Appl Ecol 2012; 49: 1316–1324. doi: 10.1111/j.1365-2664.2012.02218.x

35. Macip-Ríos R, Muñoz–Alonso A. Diversidad de lagartijas en cafetales y bosque primario en el Soconusco chiapaneco. Rev Mex Biodivers 2008; 79: 185–195.

36. Grass I, Berens DG, Peter F, Farwig N. Additive effects of exotic plant abundance and land-use intensity on plant-pollinator interactions. Oecologia 2013; 173: 913–923. doi: 10.1007/s00442-013-2688-6

37. Ims RA, Henden JA. Collapse of an arctic bird community resulting from ungulate-induced loss of erect shrubs. Biol Conserv 2012; 149: 2–5. doi: 10.1016/j.biocon.2012.02.008

38. Aguilar-Barquero V, Jiménez-Hernández F. Diversidad y distribución de palmas (Arecaceae) en tres fragmentos de bosque muy húmedo en Costa Rica. Rev Biol Trop 2009; 57: 83–92.

39. Meijer SS, Whittaker RJ, Borges PA V. The effects of land-use change on arthropod richness and abundance on Santa Maria Island (Azores): unmanaged plantations favour endemic beetles. J Insect Conserv 2011; 15: 505–522. doi: 10.1007/s10841-010-9330-2

40. Giordani P. Assessing the effects of forest management on epiphytic lichens in coppiced forests using different indicators. Plant Biosyst 2012; 146: 628–637. doi: 10.1080/11263504.2011.654136

41. Sedlock JL, Weyandt SE, Cororan L, Damerow M, Hwa S-H, Pauli B. Bat diversity in tropical forest and agro-pastoral habitats within a protected area in the Philippines. Acta Chiropterologica 2008; 10: 349–358. doi: 10.3161/150811008x414926

42. Hornung E, Tothmeresz B, Magura T, Vilisics F. Changes of isopod assemblages along an urban-suburban-rural gradient in Hungary. Eur J Soil Biol 2007; 43: 158–165. doi: 10.1016/j.ejsobi.2007.01.001

43. Norfolk O, Abdel-Dayem M, Gilbert F. Rainwater harvesting and arthropod biodiversity within an arid agro-ecosystem. Agric Ecosyst Environ 2012; 162: 8–14. doi: 10.1016/j.agee.2012.08.007

44. Poveda K, Martinez E, Kersch-Becker MF, Bonilla MA, Tscharntke T. Landscape simplification and altitude affect biodiversity, herbivory and Andean potato yield. J Appl Ecol 2012; 49: 513–522. doi: 10.1111/j.1365-2664.2012.02120.x

45. Sugiura S, Tsuru T, Yamaura Y, Makihara H. Small off-shore islands can serve as important refuges for endemic beetle conservation. J Insect Conserv 2009; 13: 377–385. doi: 10.1007/s10841-008-9185-y

46. Koivula M, Hyyrylainen V, Soininen E. Carabid beetles (Coleoptera: Carabidae) at forest-farmland edges in southern Finland. J Insect Conserv 2004; 8: 297–309. doi: 10.1007/s10841-004-0296-9

47. Blanche KR, Ludwig JA, Cunningham SA. Proximity to rainforest enhances pollination and fruit set in orchards. J Appl Ecol 2006; 43: 1182–1187. doi: 10.1111/j.1365-2664.2006.01230.x

48. Gutierrez-Lamus DL. Composition and abundance of Anura in two forest types (natural and planted) in the eastern Cordillera of Colombia. Caldasia 2004; 26: 245–264.

49. Oliveira DE, Carrijo TF, Brandão D. Species composition of termites (Isoptera) in different Cerrado vegetation physiognomies. Sociobiology 2013; 60: 190–197. doi: 10.13102/sociobiology.v60i2.190-197

50. Gomes LGL, Oostra V, Nijman V, Cleef AM, Kappelle M. Tolerance of frugivorous birds to habitat disturbance in a tropical cloud forest. Biol Conserv 2008; 141: 860–871. doi: 10.1016/j.biocon.2008.01.007

51. Blake RJ, Westbury DB, Woodcock BA, Sutton P, Potts SG. Enhancing habitat to help the plight of the bumblebee. Pest Manag Sci 2011; 67: 377–379. doi: 10.1002/ps.2136

52. Légaré J-P, Hébert C, Ruel J-C. Alternative silvicultural practices in irregular boreal forests: response of beetle assemblages. Silva Fenn 2011; 45: 937–956.

53. Richardson BA, Richardson MJ, Soto-Adames FN. Separating the effects of forest type and elevation on the diversity of litter invertebrate communities in a humid tropical forest in Puerto Rico. J Anim Ecol 2005; 74: 926–936. doi: 10.1111/j.1365-2656.2005.00990.x

54. Eigenbrod F, Hecnar SJ, Fahrig L. Accessible habitat: an improved measure of the effects of habitat loss and roads on wildlife populations. Landsc Ecol 2008; 23: 159–168. doi: 10.1007/s10980-007-9174-7

55. Vassilev K, Pedashenko H, Nikolov SC, Apostolova I, Dengler J. Effect of land abandonment on the vegetation of upland semi-natural grasslands in the Western Balkan Mts., Bulgaria. Plant Biosyst 2011; 145: 654–665. doi: 10.1080/11263504.2011.601337

56. Farwig N, Bailey D, Bochud E, Herrmann JD, Kindler E, Reusser N, et al. Isolation from forest reduces pollination, seed predation and insect scavenging in Swiss farmland. Landsc Ecol 2009; 24: 919–927. doi: 10.1007/s10980-009-9376-2

57. Louhaichi M, Salkini AK, Petersen SL. Effect of small ruminant grazing on the plant community characteristics of semiarid Mediterranean ecosystems. Int J Agric Biol 2009; 11: 681–689.

58. Gray CL, Slade EM, Mann DJ, Lewis OT. Do riparian reserves support dung beetle biodiversity and ecosystem services in oil palm-dominated tropical landscapes? Ecol Evol 2014; 4: 1049–1060. doi: 10.1002/ece3.1003

59. Ishitani M, Kotze DJ, Niemela J. Changes in carabid beetle assemblages across an urban-rural gradient in Japan. Ecography 2003; 26: 481–489. doi: 10.1034/j.1600-0587.2003.03436.x

60. Dolia J, Devy MS, Aravind NA, Kumar A. Adult butterfly communities in coffee plantations around a protected area in the Western Ghats, India. Anim Conserv 2008; 11: 26–34. doi: 10.1111/j.1469-1795.2007.00143.x

61. Kurz DJ, Nowakowski AJ, Tingley MW, Donnelly MA, Wilcove DS. Forest-land use complementarity modifies community structure of a tropical herpetofauna. Biol Conserv 2014; 170: 246–255. doi: 10.1016/j.biocon.2013.12.027

62. Sung YH, Karraker NE, Hau BCH. Terrestrial herpetofaunal assemblages in secondary forests and exotic Lophostemon confertus plantations in South China. For Ecol Manage 2012; 270: 71–77. doi: 10.1016/j.foreco.2012.01.011

63. Rousseau L, Fonte SJ, Tellez O, van der Hoek R, Lavelle P. Soil macrofauna as indicators of soil quality and land use impacts in smallholder agroecosystems of western Nicaragua. Ecol Indic 2013; 27: 71–82. doi: 10.1016/j.ecolind.2012.11.020

64. Wiafe ED, Amfo-Otu R. Forest duiker (*Cephalophus* spp.) abundance and hunting activities in the Kakum conservation area, Ghana. J Ecol Nat Environ 2012; 4: 114–118. doi: 10.5897/jene11.144

65. de Souza VM, de Souza B, Morato EF. Effect of the forest succession on the anurans (Amphibia: Anura) of the Reserve Catuaba and its periphery, Acre, southwestern Amazonia. Rev Bras Zool 2008; 25: 49–57.

66. Barrico L, Azul AM, Morais MC, Coutinho AP, Freitas H, Castro P. Biodiversity in urban ecosystems: plants and macromycetes as indicators for conservation planning in the city of Coimbra (Portugal). Landsc Urban Plan 2012; 106: 88–102. doi: 10.1016/j.landurbplan.2012.02.011

67. Chapman KA, Reich PB. Land use and habitat gradients determine bird community diversity and abundance in suburban, rural and reserve landscapes of Minnesota, USA. Biol Conserv 2007; 135: 527–541. doi: 10.1016/j.biocon.2006.10.050

68. Diekötter T, Walther-Hellwig K, Conradi M, Suter M, Frankl R. Effects of landscape elements on the distribution of the rare bumblebee species *Bombus muscorum* in an agricultural landscape. Biodivers Conserv 2006; 15: 57–68. doi: 10.1007/s10531-004-2932-9

69. Norfolk O, Eichhorn MP, Gilbert F. Traditional agricultural gardens conserve wild plants and functional richness in arid South Sinai. Basic Appl Ecol 2013; 14: 659–669. doi: 10.1016/j.baae.2013.10.004

70. Munyekenye FB, Mwangi EM, Gichuki NN. Bird species richness and abundance in different forest types at Kakamega Forest, western Kenya. Ostrich 2008; 79: 37–42. doi: 10.2989/ostrich.2008.79.1.4.361

71. Noriega JA, Realpe E, Fagua G. Diversidad de escarabajos coprofagos (Coleoptera: Scarabaeidae) en un bosque de galeria con tres estadios de alteracion Univ Sci 2007; 12: 51–63.

72. Hoffmann A, Zeller U. Influence of variations in land use intensity on species diversity and abundance of small mammals in the Nama Karoo, Namibia. Belgian J Zool 2005; 135: 91–96.

73. Cabra-García J, Bermúdez-Rivas C, Osorio AM, Chacón P. Cross-taxon congruence of alpha and beta diversity among five leaf litter arthropod groups in Colombia. Biodivers Conserv 2012; 21: 1493–1508. doi: 10.1007/s10531-012-0259-5

74. Zimmerman G, Bell FW, Woodcock J, Palmer A, Paloniemi J. Response of breeding songbirds to vegetation management in conifer plantations established in boreal mixedwoods. For Chron 2011; 87: 217–224.

75. Goulson D, Lepais O, O’Connor S, Osborne JL, Sanderson RA, Cussans J, et al. Effects of land use at a landscape scale on bumblebee nest density and survival. J Appl Ecol 2010; 47: 1207–1215. doi: 10.1111/j.1365-2664.2010.01872.x

76. Peres CA, Nascimento HS. Impact of game hunting by the Kayapo of south-eastern Amazonia: implications for wildlife conservation in tropical forest indigenous reserves. Biodivers Conserv 2006; 15: 2627–2653. doi: 10.1007/s10531-005-5406-9

77. Marin-Spiotta E, Ostertag R, Silver WL. Long-term patterns in tropical reforestation: plant community composition and aboveground biomass accumulation. Ecol Appl 2007; 17: 828–839. doi: 10.1890/06-1268

78. Nicolas V, Barriere P, Tapiero A, Colyn M. Shrew species diversity and abundance in Ziama Biosphere Reserve, Guinea: comparison among primary forest, degraded forest and restoration plots. Biodivers Conserv 2009; 18: 2043–2061. doi: 10.1007/s10531-008-9572-4

79. Sridhar H, Raman TRS, Mudappa D. Mammal persistence and abundance in tropical rainforest remnants in the southern Western Ghats, India. Curr Sci 2008; 94: 748–757.

80. Vergara PM, Simonetti JA. Avian responses to fragmentation of the Maulino Forest in central Chile. Oryx 2004; 38: 383–388. doi: 10.1017/s0030605304000742

81. Brunet J, Valtinat K, Mayr ML, Felton A, Lindbladh M, Bruun HH. Understory succession in post-agricultural oak forests: habitat fragmentation affects forest specialists and generalists differently. For Ecol Manage 2011; 262: 1863–1871. doi: 10.1016/j.foreco.2011.08.007

82. Baeten L, Velghe D, Vanhellemont M, De Frenne P, Hermy M, Verheyen K. Early trajectories of spontaneous vegetation recovery after intensive agricultural land use. Restor Ecol 2010; 18: 379–386. doi: 10.1111/j.1526-100X.2009.00627.x

83. Verboven HAF, Brys R, Hermy M. Sex in the city: reproductive success of *Digitalis purpurea* in a gradient from urban to rural sites. Landsc Urban Plan 2012; 106: 158–164. doi: 10.1016/j.landurbplan.2012.02.015

84. Haarmeyer DH, Schmiedel U, Dengler J, Bosing BM. How does grazing intensity affect different vegetation types in arid Succulent Karoo, South Africa? Implications for conservation management. Biol Conserv 2010; 143: 588–596. doi: 10.1016/j.biocon.2009.11.008

85. Freire GD, Motta PC. Effects of experimental fire regimes on the abundance and diversity of cursorial arachnids of Brazilian savannah (Cerrado biome). J Arachnol 2011; 39: 263–272. doi: 10.1636/cp10-85.1

86. Scott KA, Setterfield SA, Douglas MM, Andersen AN. Fire tolerance of perennial grass tussocks in a savanna woodland. Austral Ecol 2010; 35: 858–861. doi: 10.1111/j.1442-9993.2009.02091.x

87. Gheler-Costa C, Vettorazzi CA, Pardini R, Verdade LM. The distribution and abundance of small mammals in agroecosystems of southeastern Brazil. Mammalia 2012; 76: 185–191. doi: 10.1515/mammalia-2011-0109

88. Frizzo TLM, Vasconcelos HL. The potential role of scattered trees for ant conservation in an agriculturally dominated Neotropical landscape. Biotropica 2013; 45: 644–651. doi: 10.1111/btp.12045

89. Scott DM, Brown D, Mahood S, Denton B, Silburn A, Rakotondraparany F. The impacts of forest clearance on lizard, small mammal and bird communities in the arid spiny forest, southern Madagascar. Biol Conserv 2006; 127: 72–87. doi: 10.1016/j.biocon.2005.07.014

90. Borges PA V, Lobo JM, de Azevedo EB, Gaspar CS, Melo C, Nunes L V. Invasibility and species richness of island endemic arthropods: a general model of endemic vs. exotic species. J Biogeogr 2006; 33: 169–187. doi: 10.1111/j.1365-2699.2005.01324.x

91. Shuler RE, Roulston TH, Farris GE. Farming practices influence wild pollinator populations on squash and pumpkin. J Econ Entomol 2005; 98: 790–795. doi: 10.1603/0022-0493-98.3.790

92. Liu YH, Axmacher JC, Wang CL, Li LT, Yu ZR. Ground beetle (Coleoptera: Carabidae) assemblages of restored semi-natural habitats and intensively cultivated fields in Northern China. Restor Ecol 2012; 20: 234–239. doi: 10.1111/j.1526-100X.2010.00755.x

93. Su ZM, Zhang RZ, Qiu JX. Decline in the diversity of willow trunk-dwelling weevils (Coleoptera: Curculionoidea) as a result of urban expansion in Beijing, China. J Insect Conserv 2011; 15: 367–377. doi: 10.1007/s10841-010-9310-6

94. Santana J, Porto M, Gordinho L, Reino L, Beja P. Long-term responses of Mediterranean birds to forest fuel management. J Appl Ecol 2012; 49: 632–643. doi: 10.1111/j.1365-2664.2012.02141.x

95. Reis YT, Cancello EM. Termite (Insecta, Isoptera) richness in primary and secondary Atlantic Forest in southeastern Bahia. Iheringia Ser Zool 2007; 97: 229–234.

96. Paritsis J, Aizen MA. Effects of exotic conifer plantations on the biodiversity of understory plants, epigeal beetles and birds in *Nothofagus dombeyi* forests. For Ecol Manage 2008; 255: 1575–1583. doi: 10.1016/j.foreco.2007.11.015

97. Domínguez E, Bahamonde N, Muñoz-Escobar C. Efectos de la extracción de turba sobre la composición y estructura de una turbera de Sphagnum explotada y abandonada hace 20 años, Chile. An Inst Patagon 2012; 40: 37–45. doi: 10.4067/s0718-686x2012000200003

98. Gendreau-Berthiaume B, Kneeshaw DD, Harvey BD. Effects of partial cutting and partial disturbance by wind and insects on stand composition, structure and growth in boreal mixedwoods. Forestry 2012; 85: 551–565. doi: 10.1093/forestry/cps051

99. Martin PS, Gheler-Costa C, Lopes PC, Rosalino LM, Verdade LM. Terrestrial non-volant small mammals in agro-silvicultural landscapes of Southeastern Brazil. For Ecol Manage 2012; 282: 185–195. doi: 10.1016/j.foreco.2012.07.002

100. Cerezo A, Conde MC, Poggio SL. Pasture area and landscape heterogeneity are key determinants of bird diversity in intensively managed farmland. Biodivers Conserv 2011; 20: 2649–2667. doi: 10.1007/s10531-011-0096-y

101. Ewers RM, Thorpe S, Didham RK. Synergistic interactions between edge and area effects in a heavily fragmented landscape. Ecology 2007; 88: 96–106. doi: 10.1890/0012-9658(2007)88[96:sibeaa]2.0.co;2

102. Page N V, Qureshi Q, Rawat GS, Kushalappa CG. Plant diversity in sacred forest fragments of Western Ghats: a comparative study of four life forms. Plant Ecol 2010; 206: 237–250. doi: 10.1007/s11258-009-9638-8

103. Jacobs CT, Scholtz CH, Escobar F, Davis AL V. How might intensification of farming influence dung beetle diversity (Coleoptera: Scarabaeidae) in Maputo Special Reserve (Mozambique)? J Insect Conserv 2010; 14: 389–399. doi: 10.1007/s10841-010-9270-x

104. Pons P, Wendenburg C. The impact of fire and forest conversion into savanna on the bird communities of West Madagascan dry forests. Anim Conserv 2005; 8: 183–193. doi: 10.1017/s1367943005001940

105. Navarro IL, Roman AK, Gomez FH, Perez HA. Seasonal variation in dung beetles (Coleoptera: Scarabaeidae: Scarabaeinae) from Serrania de Coraza, Sucre (Colombia). Rev Colomb Cienc Anim 2011; 3: 102–110.

106. Higuera D, Wolf JHD. Vascular epiphytes in dry oak forests show resilience to anthropogenic disturbance, Cordillera Oriental, Colombia. Caldasia 2010; 32: 161–174.

107. Helden AJ, Leather SR. Biodiversity on urban roundabouts ‒ Hemiptera, management and the species-area relationship. Basic Appl Ecol 2004; 5: 367–377. doi: 10.1016/j.baae.2004.06.004

108. Billeter R, Liira J, Bailey D, Bugter R, Arens P, Augenstein I, et al. Indicators for biodiversity in agricultural landscapes: a pan-European study. J Appl Ecol 2008; 45: 141–150. doi: 10.1111/j.1365-2664.2007.01393.x

109. Turner EC, Foster WA. The impact of forest conversion to oil palm on arthropod abundance and biomass in Sabah, Malaysia. J Trop Ecol 2009; 25: 23–30. doi: 10.1017/s0266467408005658

110. Buddle CM, Shorthouse DP. Effects of experimental harvesting on spider (Araneae) assemblages in boreal deciduous forests. Can Entomol 2008; 140: 437–452. doi: 10.4039/n07-LS01

111. Lo-Man-Hung NF, Gardner TA, Ribeiro-Júnior MA, Barlow J, Bonaldo AB. The value of primary, secondary, and plantation forests for Neotropical epigeic arachnids. J Arachnol 2008; 36: 394–401. doi: 10.1636/ct07-136.1

112. Joubert L, Esler KJ, Privett SDJ. The effect of ploughing and augmenting natural vegetation with commercial fynbos species on the biodiversity of Overberg Sandstone fynbos on the Agulhas Plain, South Africa. South Afr J Bot 2009; 75: 526–531. doi: 10.1016/j.sajb.2009.05.002

113. Winfree R, Griswold T, Kremen C. Effect of human disturbance on bee communities in a forested ecosystem. Conserv Biol 2007; 21: 213–223. doi: 10.1111/j.1523-1739.2006.00574.x

114. Clarke FM, Rostant L V, Racey PA. Life after logging: post-logging recovery of a neotropical bat community. J Appl Ecol 2005; 42: 409–420. doi: 10.1111/j.1365-2664.2005.01024.x

115. Jonsell M. Old park trees as habitat for saproxylic beetle species. Biodivers Conserv 2012; 21: 619–642. doi: 10.1007/s10531-011-0203-0

116. Nöske NM, Hilt N, Werner FA, Brehm G, Fiedler K, Sipman HJM, et al. Disturbance effects on diversity of epiphytes and moths in a montane forest in Ecuador. Basic Appl Ecol 2008; 9: 4–12. doi: 10.1016/j.baae.2007.06.014

117. Baral SK, Katzensteiner K. Diversity of vascular plant communities along a disturbance gradient in a central mid-hill community forest of Nepal. Banko Janakari 19: 3–7. doi: 10.3126/banko.v19i1.2176

118. Oke C. Land snail diversity in post extraction secondary forest reserves in Edo State, Nigeria. Afr J Ecol 2013; 51: 244–254. doi: 10.1111/aje.12029

119. Brearley FQ. Below-ground secondary succession in tropical forests of Borneo. J Trop Ecol 2011; 27: 413–420. doi: 10.1017/s0266467411000149

120. Carrijo TF, Brandao D, de Oliveira DE, Costa DA, Santos T. Effects of pasture implantation on the termite (Isoptera) fauna in the Central Brazilian Savanna (Cerrado). J Insect Conserv 2009; 13: 575–581. doi: 10.1007/s10841-008-9205-y

121. Kazerani F, S. K, Grichanov I. Diversity of the genus *Dolichopus* Latreille in three different habitats of East Azerbaijan Province, with new records for Iran. Arx Miscel·lània Zoològica. 2013; 11: 134–152.

122. Siebert SJ. Patterns of plant species richness of temperate and tropical grassland in South Africa. Plant Ecol Evol 2011; 144: 249–254. doi: 10.5091/plecevo.2011.501

123. Bartolommei P, Mortelliti A, Pezzo F, Puglisi L. Distribution of nocturnal birds (Strigiformes and Caprimulgidae) in relation to land-use types, extent and configuration in agricultural landscapes of Central Italy. Rend Lincei 2013; 24: 13–21. doi: 10.1007/s12210-012-0211-3

124. Chauvat M, Wolters V, Dauber J. Response of collembolan communities to land-use change and grassland succession. Ecography 2007; 30: 183–192. doi: 10.1111/j.2007.0906-7590.04888.x

125. O’Connor TG. Influence of land use on plant community composition and diversity in Highland Sourveld grassland in the southern Drakensberg, South Africa. J Appl Ecol 2005; 42: 975–988. doi: 10.1111/j.1365-2664.2005.01065.x

126. Mallari NAD, Collar NJ, Lee DC, McGowan PJK, Wilkinson R, Marsden SJ. Population densities of understorey birds across a habitat gradient in Palawan, Philippines: implications for conservation. Oryx 2011; 45: 234–242. doi: 10.1017/s0030605310001031

127. Pineda E, Halffter G. Species diversity and habitat fragmentation: frogs in a tropical montane landscape in Mexico. Biol Conserv 2004; 117: 499–508. doi: 10.1016/j.biocon.2003.08.009

128. Sheil D, Puri RK, Basuki I, van Heist M, Wan M, Liswanti N, et al. Exploring biological diversity, environment and local people’s perspectives in forest landscapes: methods for a multidisciplinary landscape assessment. Center for International Forestry Research; 2002.

129. Barratt BIP, Ferguson CM, Logan RAS, Barton D, Bell NL, Sarathchandra SU, et al. Biodiversity of indigenous tussock grassland sites in Otago, Canterbury and the central North Island of New Zealand I. The macro‐invertebrate fauna. J R Soc New Zeal 2005; 35: 287–301. doi: 10.1080/03014223.2005.9517785

130. Berry NJ, Phillips OL, Lewis SL, Hill JK, Edwards DP, Tawatao NB, et al. The high value of logged tropical forests: lessons from northern Borneo. Biodivers Conserv 2010; 19: 985–997. doi: 10.1007/s10531-010-9779-z

131. Noreika N. New records of rare species of Coleoptera found in Ukmergė district in 2004-2005. New Rare Lith Insect Species 2009; 21: 68–71.

132. Summerville KS. Managing the forest for more than the trees: effects of experimental timber harvest on forest Lepidoptera. Ecol Appl 2011; 21: 806–816. doi: 10.1890/10-0715.1

133. Delabie JHC, Cereghino R, Groc S, Dejean A, Gibernau M, Corbara B, et al. Ants as biological indicators of Wayana Amerindian land use in French Guiana. C R Biol 2009; 332: 673–684. doi: 10.1016/j.crvi.2009.01.006

134. Kati V, Zografou K, Tzirkalli E, Chitos T, Willemse L. Butterfly and grasshopper diversity patterns in humid Mediterranean grasslands: the roles of disturbance and environmental factors. J Insect Conserv 2012; 16: 807–818. doi: 10.1007/s10841-012-9467-2

135. Oke OC, Chokor JU. The effect of land use on snail species richness and diversity in the tropical rainforest of south-western Nigeria. Afr Sci 2009; 10: 95–108

136. Furlani D, Ficetola GF, Colombo G, Ugurlucan M, De Bernardi F. Deforestation and the structure of frog communities in the Humedale Terraba-Sierpe, Costa Rica. Zoolog Sci 2009; 26: 197–202. doi: 10.2108/zsj.26.197

137. Jolli V, Pandit MK. Monitoring pheasants (Phasianidae) in the western Himalayas to measure the impact of hydro-electric projects. Ring 2011; 33: 37–46. doi: 10.2478/v10050-011-0003-7

138. Kittle AM, Watson AC, Chanaka Kumara PH, Nimalka Sanjeewani HK. Status and distribution of the leopard in the central hills of Sri Lanka. Cat News 2012; 56: 28–31.

139. Edenius L, Mikusinski G, Bergh J. Can repeated fertilizer applications to young Norway spruce enhance avian diversity in intensively managed forests? Ambio 2011; 40: 521–527. doi: 10.1007/s13280-011-0137-5

140. Walker S, Wilson DJ, Norbury G, Monks A, Tanentzap AJ. Complementarity of indigenous flora in shrublands and grasslands in a New Zealand dryland landscape. N Z J Ecol 2014; 38: 230–241.

141. Krauss J, Bommarco R, Guardiola M, Heikkinen RK, Helm A, Kuussaari M, et al. Habitat fragmentation causes immediate and time-delayed biodiversity loss at different trophic levels. Ecol Lett 2010; 13: 597–605. doi: 10.1111/j.1461-0248.2010.01457.x

142. Politi N, Hunter Jr. M, Rivera L. Assessing the effects of selective logging on birds in Neotropical piedmont and cloud montane forests. Biodivers Conserv 2012; 21: 3131–3155. doi: 10.1007/s10531-012-0358-3

143. Lo-Man-Hung NF, Marichal R, Candiani DF, Carvalho LS, Indicatti RP, Bonaldo AB, et al. Impact of different land management on soil spiders (Arachnida: Araneae) in two Amazonian areas of Brazil and Colombia. J Arachnol 2011; 39: 296–302. doi: 10.1636/cp10-89.1

144. Milder JC, DeClerck FAJ, Sanfiorenzo A, Sanchez DM, Tobar DE, Zuckerberg B. Effects of farm and landscape management on bird and butterfly conservation in western Honduras. Ecosphere 2010; 1: art2. doi:10.1890/es10-00003.1

145. Andersen AN, Hoffmann BD. Conservation value of low fire frequency in tropical savannas: ants in monsoonal northern Australia. Austral Ecol 2011; 36: 497–503. doi: 10.1111/j.1442-9993.2010.02151.x

146. Hilje B, Aide TM. Recovery of amphibian species richness and composition in a chronosequence of secondary forests, northeastern Costa Rica. Biol Conserv 2012; 146: 170–176. doi: 10.1016/j.biocon.2011.12.007

147. Garmendia A, Arroyo-Rodriguez V, Estrada A, Naranjo EJ, Stoner KE. Landscape and patch attributes impacting medium- and large-sized terrestrial mammals in a fragmented rain forest. J Trop Ecol 2013; 29: 331–344. doi: 10.1017/s0266467413000370

148. D’Aniello B, Stanislao I, Bonelli S, Balletto E. Haying and grazing effects on the butterfly communities of two Mediterranean-area grasslands. Biodivers Conserv 2011; 20: 1731–1744. doi: 10.1007/s10531-011-0058-4

149. Paradis S, Work TT. Partial cutting does not maintain spider assemblages within the observed range of natural variability in Eastern Canadian black spruce forests. For Ecol Manage 2011; 262: 2079–2093. doi: 10.1016/j.foreco.2011.08.032

150. Zaitsev AS, Wolters V, Waldhardt R, Dauber J. Long-term succession of oribatid mites after conversion of croplands to grasslands. Appl Soil Ecol 2006; 34: 230–239. doi: 10.1016/j.apsoil.2006.01.005

151. Golodets C, Kigel J, Sternberg M. Recovery of plant species composition and ecosystem function after cessation of grazing in a Mediterranean grassland. Plant Soil 2010; 329: 365–378. doi: 10.1007/s11104-009-0164-1

152. Shahabuddin G, Kumar R. Effects of extractive disturbance on bird assemblages, vegetation structure and floristics in tropical scrub forest, Sariska Tiger Reserve, India. For Ecol Manage 2007; 246: 175–185. doi: 10.1016/j.foreco.2007.03.061

153. Ström L, Hylander K, Dynesius M. Different long-term and short-term responses of land snails to clear-cutting of boreal stream-side forests. Biol Conserv 2009; 142: 1580–1587. doi: 10.1016/j.biocon.2009.02.028

154. Norton DA, Espie PR, Murray W, Murray J. Influence of pastoral management on plant biodiversity in a depleted short tussock grassland, Mackenzie Basin. N Z J Ecol 2006; 30: 335–344. doi: 10092/26

155. Samnegård U, Persson AS, Smith HG. Gardens benefit bees and enhance pollination in intensively managed farmland. Biol Conserv 2011; 144: 2602–2606. doi: 10.1016/j.biocon.2011.07.008

156. Savage J, Wheeler TA, Moores AMA, Taillefer AG. Effects of habitat size, vegetation cover, and surrounding land use on Diptera diversity in temperate Nearctic bogs. Wetlands 2011; 31: 125–134. doi: 10.1007/s13157-010-0133-8

157. Römbke J, Schmidt P, Höfer H. The earthworm fauna of regenerating forests and anthropogenic habitats in the coastal region of Paraná. Pesqui Agropecu Bras 2009; 44: 1040–1049. doi: 10.1590/s0100-204x2009000800037

158. Cockle KL, Leonard ML, Bodrati AA. Presence and abundance of birds in an Atlantic forest reserve and adjacent plantation of shade-grown yerba mate, in Paraguay. Biodivers Conserv 2005; 14: 3265–3288. doi: 10.1007/s10531-004-0446-0

159. Kutt AS, Vanderduys EP, O’Reagain P. Spatial and temporal effects of grazing management and rainfall on the vertebrate fauna of a tropical savanna. Rangel J 2012; 34: 173–182. doi: 10.1071/rj11049

160. Barratt BIP, Worner SP, Affeld K, Ferguson CM, Barton DM, Bell NL, et al. Biodiversity of indigenous tussock grassland sites in Otago, Canterbury and the Central North Island of New Zealand VI. Coleoptera biodiversity, community structure, exotic species invasion, and the effect of disturbance by agricultural development. J R Soc New Zeal 2012; 42: 217–239. doi: 10.1080/03036758.2011.559664

161. Vázquez DP, Simberloff D. Ecological specialization and susceptibility to disturbance: conjectures and refutations. Am Nat 2002; 159: 606–623. doi: 10.1086/339991

162. Vu L Van, Vu CQ. Diversity pattern of butterfly communities (Lepidoptera, Papilionoidae) in different habitat types in a tropical rain forest of Southern Vietnam. ISRN Zool 2011; 2011: 1–8. doi: 10.5402/2011/818545

163. de Thoisy B, Richard-Hansen C, Goguillon B, Joubert P, Obstancias J, Winterton P, et al. Rapid evaluation of threats to biodiversity: human footprint score and large vertebrate species responses in French Guiana. Biodivers Conserv 2010; 19: 1567–1584. doi: 10.1007/s10531-010-9787-z

164. Giordani P, Incerti G, Rizzi G, Ginaldi F, Viglione S, Rellini I, et al. Land use intensity drives the local variation of lichen diversity in Mediterranean ecosystems sensitive to desertification. Bibl Lichenol 2010; 105: 139–148.

165. Kutt AS, Woinarski JCZ. The effects of grazing and fire on vegetation and the vertebrate assemblage in a tropical savanna woodland in north-eastern Australia. J Trop Ecol 2007; 23: 95–106. doi: 10.1017/s0266467406003579

166. Maeto K, Sato S. Impacts of forestry on ant species richness and composition in warm-temperate forests of Japan. For Ecol Manage 2004; 187: 213–223. doi: 10.1016/s0378-1127(03)00333-5

167. Borges SH. Bird assemblages in secondary forests developing after slash-and-burn agriculture in the Brazilian Amazon. J Trop Ecol 2007; 23: 469–477. doi: 10.1017/s0266467407004105

168. Ge BM, Li ZX, Zhang DZ, Zhang HB, Liu ZT, Zhou CL, et al. Communities of soil macrofauna in green spaces of an urbanizing city at east China. Rev Chil Hist Nat 2012; 85: 219–226.

169. Struebig MJ, Kingston T, Zubaid A, Mohd-Adnan A, Rossiter SJ. Conservation value of forest fragments to Palaeotropical bats. Biol Conserv 2008; 141: 2112–2126. doi: 10.1016/j.biocon.2008.06.009

170. Urbina-Cardona JN, Olivares-Perez M, Reynoso VH. Herpetofauna diversity and microenvironment correlates across a pasture-edge-interior ecotone in tropical rainforest fragments in the Los Tuxtlas Biosphere Reserve of Veracruz, Mexico. Biol Conserv 2006; 132: 61–75. doi: 10.1016/j.biocon.2006.03.014

171. Báldi A, Batáry P, Erdős S. Effects of grazing intensity on bird assemblages and populations of Hungarian grasslands. Agric Ecosyst Environ 2005; 108: 251–263. doi: 10.1016/j.agee.2005.02.006

172. Threlfall CG, Law B, Banks PB. Sensitivity of insectivorous bats to urbanization: implications for suburban conservation planning. Biol Conserv 2012; 146: 41–52. doi: 10.1016/j.biocon.2011.11.026

173. Meyer B, Gaebele V, Steffan-Dewenter ID. Patch size and landscape effects on pollinators and seed set of the horseshoe vetch, *Hippocrepis comosa*, in an agricultural landscape of central Europe. Entomol Gen 2007; 30: 173–185.

174. O’Farrell PJ, Donaldson JS, Hoffman MT, Mader AD. Small mammal diversity and density on the Bokkeveld escarpment, South Africa ‒ implications for conservation and livestock predation. Afr Zool 2008; 43: 117–124. doi: 10.3377/1562-7020(2008)43[117:SMDADO]2.0.CO;2

175. Woodcock BA, Potts SG, Pilgrim E, Ramsay AJ, Tscheulin T, Parkinson A, et al. The potential of grass field margin management for enhancing beetle diversity in intensive livestock farms. J Appl Ecol 2007; 44: 60–69.

176. Fredriksson GM, Danielsen LS, Swenson JE. Impacts of El Nino related drought and forest fires on sun bear fruit resources in lowland dipterocarp forest of East Borneo. Biodivers Conserv 2007; 16: 1823-1838. doi: 10.1007/s10531-006-9075-0

177. Kumar R, Shahabuddin G. Effects of biomass extraction on vegetation structure, diversity and composition of forests in Sariska Tiger Reserve, India. Environ Conserv 2005; 32: 248–259

178. Marshall EJP, West TM, Kleijn D. Impacts of an agri-environment field margin prescription on the flora and fauna of arable farmland in different landscapes. Agric Ecosyst Environ 2006; 113: 36–44.

179. Rubio A V, Simonetti JA. Lizard assemblages in a fragmented landscape of central Chile. Eur J Wildl Res 2011; 57: 195–199. doi: 10.1007/s10344-010-0434-5

180. Azhar B, Lindenmayer DB, Wood J, Fischer J, Manning A, Mcelhinny C, et al. The influence of agricultural system, stand structural complexity and landscape context on foraging birds in oil palm landscapes. Ibis 2013; 155: 297-312. doi: 10.1111/ibi.12025

181. Ofori-Boateng C, Oduro W, Hillers A, Norris K, Oppong SK, Adum GB, et al. Differences in the effects of selective logging on amphibian assemblages in three west African forest types. Biotropica 2013; 45: 94–101. doi: 10.1111/j.1744-7429.2012.00887.x

182. D’Cruze N, Kumar S. Effects of anthropogenic activities on lizard communities in northern Madagascar. Anim Conserv 2011; 14: 542–552.

183. Hu C, Cao ZP. Nematode community structure under compost and chemical fertilizer management practice, in the north China plain. Exp Agric 2008; 44: 485–496. doi: 10.1017/s0014479708006716

184. Jalilova G, de Groot J, Vacik H. Evaluating the effects of habitats on birds in the walnut fruit forests: a case study from Kyrgyzstan. Biodiversity 2013; 14: 97–110. doi: 10.1080/14888386.2013.789399

185. Hernández L, Delgado L, Meier W, Duran C. Empobrecimiento de bosques fragmentados en el norte de la Gran Sabana, Venezuela. Interciencia 2012; 37: 891–898.

186. Dumont B, Farruggia A, Garel JP, Bachelard P, Boitier E, Frain M. How does grazing intensity influence the diversity of plants and insects in a species-rich upland grassland on basalt soils? Grass Forage Sci 2009; 64: 92–105. doi: 10.1111/j.1365-2494.2008.00674.x

187. Cleary DFR, Mooers AO, Eichhorn KAO, van Tol J, de Jong R, Menken SBJ. Diversity and community composition of butterflies and odonates in an ENSO-induced fire affected habitat mosaic: a case study from East Kalimantan, Indonesia. Oikos 2004; 105: 426–446. doi: 10.1111/j.0030-1299.2004.12219.x

188. Yoshikura S, Yasui S, Kamijo T. Comparative study of forest-dwelling bats’ abundances and species richness between old-growth forests and conifer plantations in Nikko National Park, central Japan. Mammal Study 2011; 36: 189–198. doi: 10.3106/041.036.0402

189. McCarthy JL, McCarthy KP, Fuller TK, McCarthy TM. Assessing variation in wildlife biodiversity in the Tien Shan Mountains of Kyrgyzstan using ancillary camera-trap photos. Mt Res Dev 2010; 30: 295–301. doi: 10.1659/mrd-journal-d-09-00080.1

190. Kessler M, Kessler PJA, Gradstein SR, Bach K, Schmull M, Pitopang R. Tree diversity in primary forest and different land use systems in Central Sulawesi, Indonesia. Biodivers Conserv 2005; 14: 547–560. doi: 10.1007/s10531-004-3914-7

191. Safian S, Csontos G, Winkler D. Butterfly community recovery in degraded rainforest habitats in the Upper Guinean Forest Zone (Kakum forest, Ghana). J Insect Conserv 2011; 15: 351–359. doi: 10.1007/s10841-010-9343-x

192. Hietz P. Conservation of vascular epiphyte diversity in Mexican coffee plantations. Conserv Biol 2005; 19: 391–399. doi: 10.1111/j.1523-1739.2005.00145.x

193. Bernard H, Fjeldsa J, Mohamed M. A case study on the effects of disturbance and conversion of tropical lowland rain forest on the non-volant small mammals in north Borneo: management implications. Mammal Study 2009; 34: 85–96. doi: 10.3106/041.034.0204

194. Arbeláez-Cortés E, Rodríguez-Correa HA, Restrepo-Chica M. Mixed bird flocks: patterns of activity and species composition in a region of the Central Andes of Colombia. Rev Mex Biodivers 2011; 82: 639–651.

195. Sosa RA, Benz VA, Galea JM, Poggio Herrero IV. Efecto del grado de disturbio sobre el ensamble de aves en la reserva provincial Parque Luro, La Pampa, Argentina. Rev la Asoc Argentina Ecol Paisajes 2010; 1: 101–110.

196. Buczkowski G. Extreme life history plasticity and the evolution of invasive characteristics in a native ant. Biol Invasions 2010; 12: 3343–3349. doi: 10.1007/s10530-010-9727-6

197. Oertli S, Muller A, Dorn S. Ecological and seasonal patterns in the diversity of a species-rich bee assemblage (Hymenoptera: Apoidea: Apiformes). Eur J Entomol 2005; 102: 53–63. doi: 10.1016/j.biocon.2005.05.014

198. Baur B, Cremene C, Groza G, Rakosy L, Schileyko AA, Baur A, et al. Effects of abandonment of subalpine hay meadows on plant and invertebrate diversity in Transylvania, Romania. Biol Conserv 2006; 132: 261–273. doi: 10.1016/j.biocon.2006.04.018

199. Faruk A, Belabut D, Ahmad N, Knell RJ, Garner TWJ. Effects of oil-palm plantations on diversity of tropical anurans. Conserv Biol 2013; 27: 615–624. doi: 10.1111/cobi.12062

200. Connop S, Hill T, Steer J, Shaw P. Microsatellite analysis reveals the spatial dynamics of *Bombus humilis* and *Bombus sylvarum*. Insect Conserv Divers 2011; 4: 212–221. doi: 10.1111/j.1752-4598.2010.00116.x

201. Jung TS, Powell T. Spatial distribution of meadow jumping mice (*Zapus hudsonius*) in logged boreal forest of northwestern Canada. Mamm Biol 2011; 76: 678–682. doi: 10.1016/j.mambio.2011.08.002

202. Endo W, Peres CA, Salas E, Mori S, Sanchez-Vega JL, Shepard GH, et al. Game vertebrate densities in hunted and nonhunted forest sites in Manu National Park, Peru. Biotropica 2010; 42: 251–261. doi: 10.1111/j.1744-7429.2009.00546.x

203. Virgilio M, Backeljau T, Emeleme R, Juakali JL, De Meyer M. A quantitative comparison of frugivorous tephritids (Diptera: Tephritidae) in tropical forests and rural areas of the Democratic Republic of Congo. Bull Entomol Res 2011; 101: 591–597. doi: 10.1017/s0007485311000216

204. Kőrösi Á, Batáry P, Orosz A, Rédei D, Báldi A. Effects of grazing, vegetation structure and landscape complexity on grassland leafhoppers (Hemiptera: Auchenorrhyncha) and true bugs (Hemiptera: Heteroptera) in Hungary. Insect Conserv Divers 2012; 5: 57–66. doi: 10.1111/j.1752-4598.2011.00153.x

205. Quintero C, Morales CL, Aizen MA. Effects of anthropogenic habitat disturbance on local pollinator diversity and species turnover across a precipitation gradient. Biodivers Conserv 2010; 19: 257–274. doi: 10.1007/s10531-009-9720-5

206. da Silva PG. Espécies de Scarabaeinae (Coleoptera: Scarabaeidae) de fragmentos florestais com diferentes níveis de alteração em Santa Maria, Rio Grande do Sul. MSc Thesis, Universidade Federal de Santa Maria. 2011. http://repositorio.ufsm.br/bitstream/handle/1/5272/SILVA,%20PEDRO%20GIOVANI%20DA.pdf

207. Castro-Luna AA, Sosa VJ, Castillo-Campos G. Bat diversity and abundance associated with the degree of secondary succession in a tropical forest mosaic in south-eastern Mexico. Anim Conserv 2007; 10: 219–228. doi: 10.1111/j.1469-1795.2007.00097.x

208. Zaitsev AS, Chauvat M, Pflug A, Wolters V. Oribatid mite diversity and community dynamics in a spruce chronosequence. Soil Biol Biochem 2002; 34: 1919–1927. doi: 10.1016/s0038-0717(02)00208-0

209. Nakashima Y, Inoue E, Akomo-Okoue EF. Population density and habitat preferences of forest duikers in Moukalaba-Doudou National Park, Gabon. Afr Zool 2013; 48: 395–399. doi: 10.3377/004.048.0212

210. Bóçon R. Riqueza e abundância de aves em três estágios sucessionais da floresta ombrófila densa submontana, Antonina, Paraná. PhD Thesis, Universidade Federal do Paraná. 2010. https://acervodigital.ufpr.br/handle/1884/26214

211. Fukuda D, Tisen OB, Momose K, Sakai S. Bat diversity in the vegetation mosaic around a lowland dipterocarp forest of Borneo. Raffles Bull Zool 2009; 57: 213–221.

212. Quaranta M, Ambroselli S, Barro P, Bella S, Carini A, Celli G, et al. Wild bees in agroecosystems and semi-natural landscapes. 1997-2000 collection period in Italy. Bull Insectology 2004; 57: 11–62.

213. Dawson J, Turner C, Pileng O, Farmer A, McGary C, Walsh C, et al. Bird communities of the lower Waria Valley, Morobe Province, Papua New Guinea: a comparison between habitat types. Trop Conserv Sci 2011; 4: 317–348.

214. Blanche R, Cunningham SA. Rain forest provides pollinating beetles for atemoya crops. J Econ Entomol 2005; 98: 1193–1201.

215. Yan X-L, Bao W-K. Structure and species composition of ground bryophyte community of high-altitude young silvicultural cutovers in Rangtang County, China: evaluation on effects of clear-cutting and silvicultural management. Chinese J Plant Ecol 2008; 32: 815–824. doi: 10.3773/j.issn.1005-264x.2008.04.010

216. Moreno-Mateos D, Rey Benayas JM, Perez-Camacho L, de la Montana E, Rebollo S, Cayuela L. Effects of land use on nocturnal birds in a Mediterranean agricultural landscape. Acta Ornithol 2011; 46: 173–182. doi: 10.3161/000164511x625946

217. Breedt JAD, Dreber N, Kellner K. Post-wildfire regeneration of rangeland productivity and functionality ‒ observations across three semi-arid vegetation types in South Africa. Afr J Range Forage Sci 2013; 30: 161–167. doi: 10.2989/10220119.2013.816367

218. Benedick S, Hill JK, Mustaffa N, Chey VK, Maryati M, Searle JB, et al. Impacts of rain forest fragmentation on butterflies in northern Borneo: species richness, turnover and the value of small fragments. J Appl Ecol 2006; 43: 967–977. doi: 10.1111/j.1365-2664.2006.01209.x

219. Arroyo J, Iturrondobeitia JC, Rad C, Gonzalez-Carcedo S. Oribatid mite (Acari) community structure in steppic habitats of Burgos Province, central northern Spain. J Nat Hist 2005; 39: 3453–3470. doi: 10.1080/00222930500240346

220. Franzén M, Nilsson SG. How can we preserve and restore species richness of pollinating insects on agricultural land? Ecography 2008; 31: 698–708. doi: 10.1111/j.1600-0587.2008.05110.x

221. López-Quintero CA, Straatsma G, Franco-Molano AE, Boekhout T. Macrofungal diversity in Colombian Amazon forests varies with regions and regimes of disturbance. Biodivers Conserv 2012; 2221–2243. doi: 10.1007/s10531-012-0280-8

222. Pincheira-Ulbrich J, Rau JR, Smith-Ramirez C. Vascular epiphytes and climbing plants diversity in an agroforestal landscape in southern Chile: a comparison among native forest fragments. Bol La Soc Argentina Bot 2012; 47: 411–426.

223. McShea WJ, Stewart C, Peterson L, Erb P, Stuebing R, Giman B. The importance of secondary forest blocks for terrestrial mammals within an *Acacia*/secondary forest matrix in Sarawak, Malaysia. Biol Conserv 2009; 142: 3108–3119. doi: 10.1016/j.biocon.2009.08.009

224. Luja VH, Herrando-Perez S, Gonzalez-Solis D, Luiselli L. Secondary rain forests are not havens for reptile species in tropical Mexico. Biotropica 2008; 40: 747–757. doi: 10.1111/j.1744-7429.2008.00439.x

225. Barlow J, Overal WL, Araujo IS, Gardner TA, Peres CA. The value of primary, secondary and plantation forests for fruit-feeding butterflies in the Brazilian Amazon. J Appl Ecol 2007; 44: 1001–1012. doi: 10.1111/j.1365-2664.2007.01347.x

226. Hanley ME. Unpublished data of bee diversity in UK croplands and urban habitats. 2011.

227. Schilthuizen M, Liew TS, Bin Elahan B, Lackman-Ancrenaz I. Effects of karst forest degradation on pulmonate and prosobranch land snail communities in Sabah, Malaysian Borneo. Conserv Biol 2005; 19: 949–954. doi: 10.1111/j.1523-1739.2005.00209.x

228. Summerville KS, Crist TO. Effects of timber harvest on forest Lepidoptera: community, guild and species responses. Ecol Appl 2002; 12: 820–835. doi: 10.1890/1051-0761(2002)012[0820:eothof]2.0.co;2

229. Muchane MN, Karanja D, Wambugu GM, Mutahi JM, Masiga CW, Mugoya C, et al. Land use practices and their implications on soil macro-fauna in Maasai Mara ecosystem. Int J Biodivers Conserv 2012; 4: 500–514. doi: 10.5897/ijbc12.030

230. Liow LH, Sodhi NS, Elmqvist T. Bee diversity along a disturbance gradient in tropical lowland forests of south-east Asia. J Appl Ecol 2001; 38: 180–192. doi: 10.1046/j.1365-2664.2001.00582.x

231. Latta SC, Tinoco BA, Astudillo PX, Graham CH. Patterns and magnitude of temporal change in avian communities in the Ecuadorian Andes. Condor 2011; 113: 24–40. doi: 10.1525/cond.2011.090252

232. Suarez-Rubio M, Thomlinson JR. Landscape and patch-level factors influence bird communities in an urbanized tropical island. Biol Conserv 2009; 142: 1311–1321. doi: 10.1016/j.biocon.2008.12.035

233. Gould RK, Pejchar L, Bothwell SG, Brosi B, Wolny S, Mendenhall CD, et al. Forest restoration and parasitoid wasp communities in montane Hawai’i. PLoS One 2013; 8: e59356. doi: 10.1371/journal.pone.0059356

234. Yan XL, Bao WK, Pang XY, Zhang NX, Chen JQ. Regeneration strategies influence ground bryophyte composition and diversity after forest clearcutting. Ann For Sci 2013; 70: 845–861. doi: 10.1007/s13595-013-0323-7

235. Schüepp C, Rittiner S, Entling MH. High bee and wasp diversity in a heterogeneous tropical farming system compared to protected forest. PLoS One 2012; 7: e52109. doi: 10.1371/journal.pone.0052109

236. Moir ML, Brennan KEC, Koch JM, Majer JD, Fletcher MJ. Restoration of a forest ecosystem: the effects of vegetation and dispersal capabilities on the reassembly of plant-dwelling arthropods. For Ecol Manage 2005; 217: 294–306. doi: 10.1016/j.foreco.2005.06.012

237. Numa C, Verdu JR, Rueda C, Galante E. Comparing dung beetle species assemblages between protected areas and adjacent pasturelands in a Mediterranean savanna landscape. Rangel Ecol Manag 2012; 65: 137–143. doi: 10.2111/rem-d-10-00050.1

238. Azpiroz AB, Blake JG. Avian assemblages in altered and natural grasslands in the northern Campos of Uruguay. Condor 2009; 111: 21–35. doi: 10.1525/cond.2009.080111

239. McFrederick QS, LeBuhn G. Are urban parks refuges for bumble bees *Bombus* spp. (Hymenoptera: Apidae)? Biol Conserv 2006; 129: 372–382. doi: 10.1016/j.biocon.2005.11.004

240. Lentini PE, Martin TG, Gibbons P, Fischer J, Cunningham SA. Supporting wild pollinators in a temperate agricultural landscape: maintaining mosaics of natural features and production. Biol Conserv 2012; 149: 84–92. doi: 10.1016/j.biocon.2012.02.004

241. Naithani A, Bhatt D. Bird community structure in natural and urbanized habitats along an altitudinal gradient in Pauri district (Garhwal Himalaya) of Uttarakhand state, India. Biologia 2012; 67: 800–808. doi: 10.2478/s11756-012-0068-z

242. Julier HE, Roulston TH. Wild bee abundance and pollination service in cultivated pumpkins: farm management, nesting behavior and landscape effects. J Econ Entomol 2009; 102: 563–573. doi: 10.1603/029.102.0214

243. Kohler F, Verhulst J, van Klink R, Kleijn D. At what spatial scale do high-quality habitats enhance the diversity of forbs and pollinators in intensively farmed landscapes? J Appl Ecol 2008; 45: 753–762. doi: 10.1111/j.1365-2664.2007.01394.x

244. Hylander K, Nilsson C, Gothner T. Effects of buffer-strip retention and clearcutting on land snails in boreal riparian forests. Conserv Biol 2004; 18: 1052–1062. doi: 10.1111/j.1523-1739.2004.00199.x

245. Banks JE, Sandvik P, Keesecker L. Beetle (Coleoptera) and spider (Araneae) diversity in a mosaic of farmland, edge, and tropical forest habitats in western Costa Rica. Pan-Pac Entomol 2007; 83: 152–160. doi: 10.3956/0031-0603-83.2.152

246. Reynolds C, Symes CT. Grassland bird response to vegetation structural heterogeneity and clearing of invasive bramble. Afr Zool 2013; 48: 228–239. doi: 10.3377/004.048.0217

247. Malone L, Aulsford J, Howlett B, Scott-Dupree C, Bardol N, Donovan B. Observations on bee species visiting white clover in New Zealand pastures. J Apic Res 2010; 49: 284–286. doi:10.3896/ibra.1.49.3.09

248. Noreika N, Kotze DJ. Forest edge contrasts have a predictable effect on the spatial distribution of carabid beetles in urban forests. J Insect 2012; 16: 867–881. doi: 10.1007/s10841-012-9474-3

249. Litchwark SA. Honeybee declines in a changing landscape: interactive effects of honeybee declines and land-use intensification on pollinator communities. MSc Thesis, University of Canterbury. 2013. https://ir.canterbury.ac.nz/handle/10092/9064

250. Craig MD, Grigg AH, Hobbs RJ, Hardy GESJ. Does coarse woody debris density and volume influence the terrestrial vertebrate community in restored bauxite mines? For Ecol Manage 2014; 318: 142–150. doi: 10.1016/j.foreco.2014.01.011

251. Goulson D, Lye GC, Darvill B. Diet breadth, coexistence and rarity in bumblebees. Biodivers Conserv 2008; 17: 3269–3288. doi: 10.1007/s10531-008-9428-y

252. Pethiyagoda Jr. RS, Manamendra-Arachchi K. Endangered anurans in a novel forest in the highlands of Sri Lanka. Wildl Res 2012; 39: 641–648. doi: 10.1071/wr12079

253. MacSwiney MCG, Vilchis PL, Clarke FM, Racey PA. The importance of cenotes in conserving bat assemblages in the Yucatan, Mexico. Biol Conserv 2007; 136: 499–509. doi: 10.1016/j.biocon.2006.12.021

254. Lucas-Borja ME, Bastida F, Moreno JL, Nicolas C, Andres M, Lopez FR, et al. The effects of human trampling on the microbiological properties of soil and vegetation in Mediterranean mountain areas. L Degrad Dev 2011; 22: 383–394. doi: 10.1002/ldr.1014

255. Verdasca MJ, Leitao AS, Santana J, Porto M, Dias S, Beja P. Forest fuel management as a conservation tool for early successional species under agricultural abandonment: the case of Mediterranean butterflies. Biol Conserv 2012; 146: 14–23. doi: 10.1016/j.biocon.2011.10.031

256. Craig MD, Grigg AH, Garkaklis MJ, Hobbs RJ, Grant CD, Fleming PA, et al. Does habitat structure influence capture probabilities? A study of reptiles in a eucalypt forest. Wildl Res 2009; 36: 509–515. doi: 10.1071/wr09014

257. Pelegrin N, Bucher EH. Effects of habitat degradation on the lizard assemblage in the Arid Chaco, central Argentina. J Arid Environ 2012; 79: 13–19. doi: 10.1016/j.jaridenv.2011.11.004

258. Shafie NJ, Sah SAM, Latip NSA, Azman NM, Khairuddin NL. Diversity pattern of bats at two contrasting habitat types along Kerian River, Perak, Malaysia. Trop Life Sci Res 2011; 22: 13–22.

259. Li SN, Zou FS, Zhang Q, Sheldon FH. Species richness and guild composition in rubber plantations compared to secondary forest on Hainan Island, China. Agrofor Syst 2013; 87: 1117–1128. doi: 10.1007/s10457-013-9624-y

260. Armbrecht I, Perfecto I, Silverman E. Limitation of nesting resources for ants in Colombian forests and coffee plantations. Ecol Entomol 2006; 31: 403–410. doi: 10.1111/j.1365-2311.2006.00802.x

261. Cagle NL. Snake species distributions and temperate grasslands: a case study from the American tallgrass prairie. Biol Conserv 2008;141: 744–755. doi: 10.1016/j.biocon.2008.01.003

262. Alcayaga OE, Pizarro-Araya J, Alfaro FM, Cepeda-Pizarro J. Spiders (Arachnida, Araneae) associated to agroecosystems in the Elqui Valley (Coquimbo Region, Chile). Rev Colomb Entomol 2013; 39: 150–154.

263. Letcher SG, Chazdon RL. Rapid recovery of biomass, species richness, and species composition in a forest chronosequence in northeastern Costa Rica. Biotropica 2009; 41: 608–617. doi: 10.1111/j.1744-7429.2009.00517.x

264. Fernandez IC, Simonetti JA. Small mammal assemblages in fragmented shrublands of urban areas of Central Chile. Urban Ecosyst 2013; 16: 377–387. doi: 10.1007/s11252-012-0272-1

265. McNamara S, Erskine PD, Lamb D, Chantalangsy L, Boyle S. Primary tree species diversity in secondary fallow forests of Laos. For Ecol Manage 2012; 281: 93–99. doi: 10.1016/j.foreco.2012.06.004

266. Osgathorpe LM, Park K, Goulson D. The use of off-farm habitats by foraging bumblebees in agricultural landscapes: implications for conservation management. Apidologie 2012; 43: 113–127. doi: 10.1007/s13592-011-0083-z

267. Gove AD, Majer JD, Rico-Gray V. Methods for conservation outside of formal reserve systems: the case of ants in the seasonally dry tropics of Veracruz, Mexico. Biol Conserv 2005; 126: 328–338. doi: 10.1016/j.biocon.2005.06.008

268. Fowler RE. An investigation into bee assemblage change along an urban-rural gradient. PhD Thesis, University of Birmingham. 2014. http://etheses.bham.ac.uk/5823/

269. Power EF, Stout JC. Organic dairy farming: impacts on insect-flower interaction networks and pollination. J Appl Ecol 2011; 48: 561–569. doi: 10.1111/j.1365-2664.2010.01949.x

270. Fierro MM, Cruz-Lopez L, Sanchez D, Villanueva-Gutierrez R, Vandame R. Effect of biotic factors on the spatial distribution of stingless bees (Hymenoptera: Apidae, Meliponini) in fragmented Neotropical habitats. Neotrop Entomol 2012; 41: 95–104. doi: 10.1007/s13744-011-0009-5

271. Mudri-Stojnic S, Andric A, Jozan Z, Vujic A. Pollinator diversity (Hymenoptera and Diptera) in semi-natural habitats in Serbia during summer. Arch Biol Sci 2012; 64: 777–786. doi: 10.2298/abs1202777s

272. Raub F, Hoefer H, Scheuermann L, Brandl R. The conservation value of secondary forests in the southern Brazilian Mata Atlantica from a spider perspective. J Arachnol 2014; 42: 52–73. doi: 10.1636/p13-47.1

273. Adum GB, Eichhorn MP, Oduro W, Ofori-Boateng C, Rodel MO. Two-stage recovery of amphibian assemblages following selective logging of tropical forests. Conserv Biol 2013; 27: 354–363. doi: 10.1111/cobi.12006

274. Sam K, Koane B, Jeppy S, Novotny V. Effect of forest fragmentation on bird species richness in Papua New Guinea. J F Ornithol 2014; 85: 152–167. doi: 10.1111/jofo.12057

275. Carpenter D, Hammond PM, Sherlock E, Lidgett A, Leigh K, Eggleton P. Biodiversity of soil macrofauna in the New Forest: a benchmark study across a national park landscape. Biodivers Conserv 2012; 21: 3385–3410. doi: 10.1007/s10531-012-0369-0

276. Darvill B, Knight ME, Goulson D. Use of genetic markers to quantify bumblebee foraging range and nest density. Oikos 2004; 107: 471–478. doi: 10.1111/j.0030-1299.2004.13510.x

277. Weller B, Ganzhorn JU. Carabid beetle community composition, body size, and fluctuating asymmetry along an urban-rural gradient. Basic Appl Ecol 2004; 5: 193–201. doi: 10.1078/1439-1791-00220

278. Rey-Benayas JM, Galvan I, Carrascal LM. Differential effects of vegetation restoration in Mediterranean abandoned cropland by secondary succession and pine plantations on bird assemblages. For Ecol Manage 2010; 260: 87–95. doi: 10.1016/j.foreco.2010.04.004

279. Naoe S, Sakai S, Masaki T. Effect of forest shape on habitat selection of birds in a plantation-dominant landscape across seasons: comparison between continuous and strip forests. J For Res 2012; 17: 219–223. doi: 10.1007/s10310-011-0296-z

280. Dominguez-Haydar Y, Armbrecht I. Response of ants and their seed removal in rehabilitation areas and forests at El Cerrejon coal mine in Colombia. Restor Ecol 2010; 19: 178–184. doi: 10.1111/j.1526-100X.2010.00735.x

281. Silva FAB, Costa CMQ, Moura RC, Farias AI. Study of the dung beetle (Coleoptera: Scarabaeidae) community at two sites: Atlantic Forest and clear-cut, Pernambuco, Brazil. Environ Entomol 2010; 39: 359–367. doi: 10.1603/en09180

282. Malonza PK, Veith M. Amphibian community along elevational and habitat disturbance gradients in the Taita Hills, Kenya. Herpetotropicos 2012; 7: 7–16.

283. Fermon H, Waltert M, Vane-Wright RI, Muhlenberg M. Forest use and vertical stratification in fruit-feeding butterflies of Sulawesi, Indonesia: impacts for conservation. Biodivers Conserv 2005; 14: 333–350. doi: 10.1007/s10531-004-5054-9

284. Woinarski JCZ, Rankmore B, Hill B, Griffiths AD, Stewart A, Grace B. Fauna assemblages in regrowth vegetation in tropical open forests of the Northern Territory, Australia. Wildl Res 2009; 36: 675–690. doi: 10.1071/wr08128

285. Ribeiro DB, Freitas AVL. The effect of reduced-impact logging on fruit-feeding butterflies in Central Amazon, Brazil. J Insect Conserv 2012; 16: 733–744. doi: 10.1007/s10841-012-9458-3

286. de Sassi C, Lewis OT, Tylianakis JM. Plant-mediated and nonadditive effects of two global change drivers on an insect herbivore community. Ecology 2012; 93: 1892–1901. doi: 10.1890/11-1839.1

287. Romero-Duque LP, Jaramillo VJ, Perez-Jimenez A. Structure and diversity of secondary tropical dry forests in Mexico, differing in their prior land-use history. For Ecol Manage 2007; 253: 38–47. doi: 10.1016/j.foreco.2007.07.002

288. Nakamura A, Proctor H, Catterall CP. Using soil and litter arthropods to assess the state of rainforest restoration. Ecol Manag Restor 2003; 4: S20–S28. doi: 10.1046/j.1442-8903.4.s.3.x

289. Brandt JS, Wood EM, Pidgeon AM, Han L-X, Fang Z, Radeloff VC. Sacred forests are keystone structures for forest bird conservation in southwest China’s Himalayan Mountains. Biol Conserv 2013; 166: 34–42. doi: 10.1016/j.biocon.2013.06.014

290. Waite EM, Closs G, Van Heezik Y, Berry C, Dickinson K. Arboreal arthropod sampling methods for urban trees. J Insect Conserv 2012; 16: 931–939. doi: 10.1007/s10841-012-9480-5

291. Calviño-Cancela M, Rubido-Bará M, van Etten EJB. Do eucalypt plantations provide habitat for native forest biodiversity? For Ecol Manage 2012; 270: 153–162. doi: 10.1016/j.foreco.2012.01.019

292. Shannon G, Druce DJ, Page BR, Eckhardt HC, Grant R, Slotow R. The utilization of large savanna trees by elephant in southern Kruger National Park. J Trop Ecol 2008;24: 281–289. doi: 10.1017/s0266467408004951

293. Summerville KS, Conoan CJ, Steichen RM. Species traits as predictors of lepidopteran composition in restored and remnant tallgrass prairies. Ecol Appl 2006; 16: 891–900. doi: 10.1890/1051-0761(2006)016[0891:stapol]2.0.co;2

294. Hatfield RG, LeBuhn G. Patch and landscape factors shape community assemblage of bumble bees, *Bombus* spp. (Hymenoptera: Apidae), in montane meadows. Biol Conserv 2007; 139: 150–158. doi: 10.1016/j.biocon.2007.06.019

295. Özden Ö, Ciesla WM, Fuller WJ, Hodgson DJ. Butterfly diversity in Mediterranean islands and in Pentadaktylos *Pinus brutia* forests of Cyprus. Biodivers Conserv 2008; 17: 2821–2832. doi: 10.1007/s10531-008-9382-8

296. Schon NL, Mackay AD, Minor MA, Yeates GW, Hedley MJ. Soil fauna in grazed New Zealand hill country pastures at two management intensities. Appl Soil Ecol 2008; 40: 218–228. doi: 10.1016/j.apsoil.2008.04.007

297. Nielsen A, Steffan-Dewenter I, Westphal C, Messinger O, Potts SG, Roberts SPM, et al. Assessing bee species richness in two Mediterranean communities: importance of habitat type and sampling techniques. Ecol Res 2011; 26: 969–983. doi: 10.1007/s11284-011-0852-1

298. Malumbres-Olarte J, Barratt BIP, Vink CJ, Paterson AM, Cruickshank RH, Ferguson CM, et al. Big and aerial invaders: dominance of exotic spiders in burned New Zealand tussock grasslands. Biol Invasions 2014; 16: 2311–2322. doi: 10.1007/s10530-014-0666-5

299. Cameron SA, Lozier JD, Strange JP, Koch JB, Cordes N, Solter LF, et al. Patterns of widespread decline in North American bumble bees. Proc Natl Acad Sci USA 2011; 108: 662–667. doi: 10.1073/pnas.1014743108

300. Schon NL, Mackay AD, Yeates GW, Minor MA. Separating the effects of defoliation and dairy cow treading pressure on the abundance and diversity of soil invertebrates in pastures. Appl Soil Ecol 2010; 46: 209–221. doi: 10.1016/j.apsoil.2010.08.011

301. Lehouck V, Spanhove T, Colson L, Adringa-Davis A, Cordeiro NJ, Lens L. Habitat disturbance reduces seed dispersal of a forest interior tree in a fragmented African cloud forest. Oikos 2009; 118: 1023–1034. doi: 10.1111/j.1600-0706.2009.17300.x

302. Rodrigues MM, Uchoa MA, Ide S. Dung beetles (Coleoptera: Scarabaeoidea) in three landscapes in Mato Grosso do Sul, Brazil. Brazilian J Biol 2013; 73: 211–220.

303. Cunningham SA, Schellhorn NA, Marcora A, Batley M. Movement and phenology of bees in a subtropical Australian agricultural landscape. Austral Ecol 2013; 38: 456–464. doi: 10.1111/j.1442-9993.2012.02432.x

304. Marsh C. The birds of the Comoro Islands. PhD Thesis, Imperial College London. 2005.

305. Urbina-Cardona JN, Londoño-Murcia MC, García-Ávila DG. Spatio-temporal dymanics of snake diversity in four habitats with different degrees of anthropogenic disturbance in the Gorgona Island National Natural Park in the Colombian Pacific. Caldasia 2008; 30: 479–493.

306. Yan X, Bao W. Evaluation of species composition and development of bryophyte community during early natural recovery progress of high-altitude spruce cutovers. Biodivers Sci 2008; 16: 110–117. doi: 10.3724/sp.j.1003.2008.07349

307. Lantschner M V, Rusch V, Hayes JP. Habitat use by carnivores at different spatial scales in a plantation forest landscape in Patagonia, Argentina. For Ecol Manage 2012; 269: 271–278. doi: 10.1016/j.foreco.2011.12.045

308. Mayfield MM, Ackerly D, Daily GC. The diversity and conservation of plant reproductive and dispersal functional traits in human-dominated tropical landscapes. J Ecol 2006; 94: 522–536. doi: 10.1111/j.1365-2745.2006.01108.x

309. Alguacil M del M, Torrecillas E, Hernandez G, Roldan A. Changes in the diversity of soil arbuscular mycorrhizal fungi after cultivation for biofuel production in a Guantanamo (Cuba) tropical system. PLoS One 2012; 7: e34887. doi: 10.1371/journal.pone.0034887

310. Bicknell J, Peres CA. Vertebrate population responses to reduced-impact logging in a neotropical forest. For Ecol Manage 2010; 259: 2267–2275. doi:10.1016/j.foreco.2010.02.027

311. Craig MD, Hardy GESJ, Fontaine JB, Garkakalis MJ, Grigg AH, Grant CD, et al. Identifying unidirectional and dynamic habitat filters to faunal recolonisation in restored mine-pits. J Appl Ecol 2012; 49: 919–928. doi: 10.1111/j.1365-2664.2012.02152.x

312. Fayle TM, Turner EC, Snaddon JL, Chey VK, Chung AYC, Eggleton P, et al. Oil palm expansion into rain forest greatly reduces ant biodiversity in canopy, epiphytes and leaf-litter. Basic Appl Ecol 2010; 11: 337–345. doi: 10.1016/j.baae.2009.12.009

313. Sakchoowong W, Nomura S, Ogata K, Chanpaisaeng J. Diversity of pselaphine beetles (Coleoptera: Staphylinidae: Pselaphinae) in eastern Thailand. Entomol Sci 2008; 11: 301–313. doi: 10.1111/j.1479-8298.2008.00281.x

314. Stouffer PC, Johnson EI, Bierregaard Jr. RO, Lovejoy TE. Understory bird communities in Amazonian rainforest fragments: species turnover through 25 years post-isolation in recovering landscapes. PLoS One 2011; 6: e20543. doi: 10.1371/journal.pone.0020543

315. Noriega JA, Palacio JM, Monroy-G JD, Valencia E. Estructura de un ensamblaje de escarabajos coprofagos (Coleoptera: Scarabaeinae) en tres sitios con diferente uso del suelo en Antioquia, Colombia. Actual Biol 2012; 34: 43–54.

316. Ndang’ang’a PK, Njoroge JBM, Githiru M. Vegetation composition and structure influences bird species community assemblages in the highland agricultural landscape of Nyandarua, Kenya. Ostrich 2013; 84: 171–179. doi: 10.2989/00306525.2013.860929

317. Otavo SE, Parrado-Rosselli A, Noriega JA. Scarabaeoidea superfamily (Insecta: Coleoptera) as a bioindicator element of anthropogenic disturbance in an Amazon national park. Rev Biol Trop 2013; 61: 735–752.

318. Gottschalk MS, De Toni DC, Valente VLS, Hofmann PRP. Changes in Brazilian Drosophilidae (Diptera) assemblages across an urbanisation gradient. Neotrop Entomol 2007; 36: 848–862. doi: 10.1590/s1519-566x2007000600005

319. Meyer B, Jauker F, Steffan-Dewenter I. Contrasting resource-dependent responses of hoverfly richness and density to landscape structure. Basic Appl Ecol 2009; 10: 178–186. doi: 10.1016/j.baae.2008.01.001

320. Brito I, Goss MJ, de Carvalho M, Chatagnier O, van Tuinen D. Impact of tillage system on arbuscular mycorrhiza fungal communities in the soil under Mediterranean conditions. Soil Tillage Res 2012; 121: 63–67. doi: 10.1016/j.still.2012.01.012

321. Reid JL, Harris JBC, Zahawi RA. Avian habitat preference in tropical forest restoration in southern Costa Rica. Biotropica 2012; 44: 350–359. doi: 10.1111/j.1744-7429.2011.00814.x

322. Boutin C, Martin PA, Baril A. Arthropod diversity as affected by agricultural management (organic and conventional farming), plant species, and landscape context. Ecoscience 2009; 16: 492–501. doi: 10.2980/16-4-3250

323. Granjon L, Duplantier JM. Guinean biodiversity at the edge: rodents in forest patches of southern Mali. Mamm Biol 2011; 76: 583–591. doi: 10.1016/j.mambio.2011.06.003

324. Dures SG, Cumming GS. The confounding influence of homogenising invasive species in a globally endangered and largely urban biome: does habitat quality dominate avian biodiversity? Biol Conserv 2010; 143: 768–777. doi: 10.1016/j.biocon.2009.12.019

325. Vasconcelos HL, Pacheco R, Silva RC, Vasconcelos PB, Lopes CT, Costa AN, et al. Dynamics of the leaf-litter arthropod fauna following fire in a Neotropical woodland savanna. PLoS One 2009; 4: e7762. doi: 10.1371/journal.pone.0007762

326. Isaacs-Cubides PJ, Urbina-Cardona JN. Anthropogenic disturbance and edge effects on anuran assemblages inhabiting cloud forest fragments in Colombia. Nat Conserv 2011; 9: 39–46. doi: 10.4322/natcon.2011.004

327. Waite E, Closs GP, van Heezik Y, Dickinson KJM. Resource availability and foraging of Silvereyes (*Zosterops lateralis*) in urban trees. Emu 2013; 113: 26–32. doi: 10.1071/mu11093

328. Craig MD. Unpublished data of terrestrial vertebrates in South West Australia. 2014.

329. Buczkowski G, Richmond DS. The effect of urbanization on ant abundance and diversity: a temporal examination of factors affecting biodiversity. PLoS One 2012; 7: e41729. doi: 10.1371/journal.pone.0041729

330. Mico E, Garcia-Lopez A, Brustel H, Padilla A, Galante E. Explaining the saproxylic beetle diversity of a protected Mediterranean area. Biodivers Conserv 2013; 22: 889–904. doi: 10.1007/s10531-013-0456-x

331. Schüepp C, Herrmann JD, Herzog F, Schmidt-Entling MH. Differential effects of habitat isolation and landscape composition on wasps, bees, and their enemies. Oecologia 2011; 165: 713–721. doi: 10.1007/s00442-010-1746-6

332. Bates AJ, Sadler JP, Fairbrass AJ, Falk SJ, Hale JD, Matthews TJ. Changing bee and hoverfly pollinator assemblages along an urban-rural gradient. PLoS One 2011; 6: e23459. doi: 10.1371/journal.pone.0023459

333. Schumann K, Wittig R, Thiombiano A, Becker U, Hahn K. Impact of land-use type and harvesting on population structure of a non-timber forest product-providing tree in a semi-arid savanna, West Africa. Biol Conserv 2011; 144: 2369–2376. doi: 10.1016/j.biocon.2011.06.018

334. Elek Z, Lovei GL. Patterns in ground beetle (Coleoptera: Carabidae) assemblages along an urbanisation gradient in Denmark. Acta Oecologica 2007; 32: 104–111. doi: 10.1016/j.actao.2007.03.008

335. Williams CD, Sheahan J, Gormally MJ. Hydrology and management of turloughs (temporary lakes) affect marsh fly (Sciomyzidae: Diptera) communities. Insect Conserv Divers 2009; 2: 270–283. doi: 10.1111/j.1752-4598.2009.00064.x

336. Gu W-B, Zhen-Rong Y, Dun-Xiao H. Carabid community and its fluctuation in farmland of salinity transforming area in the North China Plain: a case study in Quzhou County, Hebei Province. Biodivers Sci 2004; 12: 262–268.

337. Magura T, Horvath R, Tothmeresz B. Effects of urbanization on ground-dwelling spiders in forest patches, in Hungary. Landsc Ecol 2010; 25: 621–629. doi: 10.1007/s10980-009-9445-6

338. Bösing BM, Haarmeyer DH, Denger J, Ganzhorn JU, Schmiedel U. Effects of livestock grazing and habitat characteristics on small mammal communities in the Knersvlakte, South Africa. J Arid Environ 2014; 104: 124–131. doi: 10.1016/j.jaridenv.2014.02.011

339. Alignier A, Deconchat M. Patterns of forest vegetation responses to edge effect as revealed by a continuous approach. Ann For Sci 2013; 70: 601–609. doi: 10.1007/s13595-013-0301-0

340. Lantschner M V, Rusch V, Peyrou C. Bird assemblages in pine plantations replacing native ecosystems in NW Patagonia. Biodivers Conserv 2008; 17: 969–989. doi: 10.1007/s10531-007-9243-x

341. Hanley ME. Unpublished data of bee diversity in UK croplands. 2005.

342. Rader R, Bartomeus I, Tylianakis JM, Laliberté E. The winners and losers of land use intensification: pollinator community disassembly is non-random and alters functional diversity. Divers Distrib 2014; 20: 908–917. doi: 10.1111/ddi.12221

343. Tylianakis JM, Klein A-M, Tscharntke T. Spatiotemporal variation in the diversity of Hymenoptera across a tropical habitat gradient. Ecology 2005; 86: 3296–3302. doi: 10.1890/05-0371

344. Barlow J, Gardner TA, Araujo IS, Ávila-Pires TC, Bonaldo AB, Costa JE, et al. Quantifying the biodiversity value of tropical primary, secondary, and plantation forests. Proc Natl Acad Sci USA 2007; 104: 18555–18560. doi: 10.1073/pnas.0703333104

345. Power EF, Kelly DL, Stout JC. Organic farming and landscape structure: effects on insect-pollinated plant diversity in intensively managed grasslands. PLoS One 2012; 7: e38073. doi: 10.1371/journal.pone.0038073

346. Neuschulz EL, Botzat A, Farwig N. Effects of forest modification on bird community composition and seed removal in a heterogeneous landscape in South Africa. Oikos 2011; 120: 1371–1379. doi: 10.1111/j.1600-0706.2011.19097.x

347. Wunderle JM, Henriques LMP, Willig MR. Short-term responses of birds to forest gaps and understory: an assessment of reduced-impact logging in a lowland Amazon Forest. Biotropica 2006; 38: 235–255.

348. Slade EM, Mann DJ, Lewis OT. Biodiversity and ecosystem function of tropical forest dung beetles under contrasting logging regimes. Biol Conserv 2011; 144: 166–174. doi: 10.1016/j.biocon.2010.08.011

349. Lachat T, Attignon S, Djego J, Goergen G, Nagel P, Sinsin B, et al. Arthropod diversity in Lama forest reserve (South Benin), a mosaic of natural, degraded and plantation forests. Biodivers Conserv 2006; 15: 3–23. doi: 10.1007/s10531-004-1234-6

350. Kapoor V. Effects of rainforest fragmentation and shade-coffee plantations on spider communities in the Western Ghats, India. J Insect Conserv 2008; 12: 53–68. doi: 10.1007/s10841-006-9062-5

351. Bragagnolo C, Nogueira AA., Pinto-da-Rocha R, Pardini R. Harvestmen in an Atlantic forest fragmented landscape: evaluating assemblage response to habitat quality and quantity. Biol Conserv 2007; 139: 389–400. doi: 10.1016/j.biocon.2007.07.008

352. Vergara CH, Badano EI. Pollinator diversity increases fruit production in Mexican coffee plantations: the importance of rustic management systems. Agric Ecosyst Environ 2009; 129: 117–123. doi: 10.1016/j.agee.2008.08.001

353. Sheldon FH, Styring A, Hosner PA. Bird species richness in a Bornean exotic tree plantation: a long-term perspective. Biol Conserv 2010; 143: 399–407. doi: 10.1016/j.biocon.2009.11.004

354. Kessler M, Abrahamczyk S, Bos M, Buchori D, Putra DD, Gradstein SR, et al. Alpha and beta diversity of plants and animals along a tropical land-use gradient. Ecol Appl 2009; 19: 2142–56.

355. Horgan FG. Invasion and retreat: shifting assemblages of dung beetles amidst changing agricultural landscapes in central Peru. Biodivers Conserv 2009; 18: 3519–3541. doi: 10.1007/s10531-009-9658-7

356. Hawes J, Motta C da S, Overal WL, Barlow J, Gardner TA, Peres CA. Diversity and composition of Amazonian moths in primary, secondary and plantation forests. J Trop Ecol 2009; 25: 281–300. doi: 10.1017/S0266467409006038

357. Filgueiras BKC, Iannuzzi L, Leal IR. Habitat fragmentation alters the structure of dung beetle communities in the Atlantic Forest. Biol Conserv 2011; 144: 362–369. doi: 10.1016/j.biocon.2010.09.013

358. Cáceres NC, Nápoli RP, Casella J, Hannibal W. Mammals in a fragmented savannah landscape in south-western Brazil. J Nat Hist 2010; 44: 491–512. doi: 10.1080/00222930903477768

359. Wells K, Kalko EK V., Lakim MB, Pfeiffer M. Effects of rain forest logging on species richness and assemblage composition of small mammals in Southeast Asia. J Biogeogr 2007; 34: 1087–1099. doi: 10.1111/j.1365-2699.2006.01677.x

360. Navarrete D, Halffter G. Dung beetle (Coleoptera: Scarabaeidae: Scarabaeinae) diversity in continuous forest, forest fragments and cattle pastures in a landscape of Chiapas, Mexico: the effects of anthropogenic changes. Biodivers Conserv 2008; 17: 2869–2898. doi: 10.1007/s10531-008-9402-8

361. Davis AL V, Philips TK. Effect of deforestation on a Southwest Ghana dung beetle assemblage (Coleoptera : Scarabaeidae) at the periphery of Ankasa conservation area. Environ Entomol 2005; 34: 1081–1088. doi: 10.1603/0046-225X(2005)034

362. Barlow J, Mestre LAM, Gardner TA, Peres CA. The value of primary, secondary and plantation forests for Amazonian birds. Biol Conserv 2007; 136: 212–231. doi: 10.1016/j.biocon.2006.11.021

363. Bouyer J, Sana Y, Samandoulgou Y, Cesar J, Guerrini L, Kabore-Zoungrana C, et al. Identification of ecological indicators for monitoring ecosystem health in the trans-boundary W Regional park: a pilot study. Biol Conserv 2007; 138: 73–88. doi: 10.1016/j.biocon.2007.04.001

364. Basset Y, Missa O, Alonso A, Miller SE, Curletti G, De Meyer M, et al. Changes in arthropod assemblages along a wide gradient of disturbance in Gabon. Conserv Biol 2008; 22: 1552–63. doi: 10.1111/j.1523-1739.2008.01017.x

365. Alcala EL, Alcala AC, Dolino CN. Amphibians and reptiles in tropical rainforest fragments on Negros Island, the Philippines. Environ Conserv 2004; 31: 254–261. doi: 10.1017/S0376892904001407

366. Gardner TA, Ribeiro-Júnior MA, Barlow J, Ávila-Pires TCS, Hoogmoed MS, Peres CA. The value of primary, secondary, and plantation forests for a Neotropical herpetofauna. Conserv Biol 2007; 21: 775–87. doi: 10.1111/j.1523-1739.2007.00659.x

367. Vallan D. Effects of anthropogenic environmental changes on amphibian diversity in the rain forests of eastern Madagascar. J Trop Ecol 2002; 18: 725–742. doi: 10.1017/S026646740200247X

368. Johnson MF, Gómez A, Pinedo-Vasquez M. Land use and mosquito diversity in the Peruvian Amazon. J Med Entomol 2008; 45: 1023–1030. doi: 10.1603/0022-2585(2008)45

369. Farwig N, Sajita N, Böhning-Gaese K. Conservation value of forest plantations for bird communities in western Kenya. For Ecol Manage 2008; 255: 3885–3892. doi: 10.1016/j.foreco.2008.03.042

370. Soh MCK, Sodhi NS, Lim SL-H. High sensitivity of montane bird communities to habitat disturbance in Peninsular Malaysia. Biol Conserv 2006; 129: 149–166. doi: 10.1016/j.biocon.2005.10.030

371. Parry L, Barlow J, Peres CA. Hunting for sustainability in tropical secondary forests. Conserv Biol 2009; 23: 1270–1280. doi: 10.1111/j.1523-1739.2009.01224.x

372. O’Dea N, Whittaker RJ. How resilient are Andean montane forest bird communities to habitat degradation? Biodivers Conserv 2007; 16: 1131–1159. doi: 10.1007/s10531-006-9095-9
